# Endothelial YAP/TAZ activation promotes atherosclerosis in a mouse model of Hutchinson-Gilford progeria syndrome

**DOI:** 10.1172/JCI173448

**Published:** 2024-10-01

**Authors:** Ana Barettino, Cristina González-Gómez, Pilar Gonzalo, María J. Andrés-Manzano, Carlos R. Guerrero, Francisco M. Espinosa, Rosa M. Carmona, Yaazan Blanco, Beatriz Dorado, Carlos Torroja, Fátima Sánchez-Cabo, Ana Quintas, Alberto Benguría, Ana Dopazo, Ricardo García, Ignacio Benedicto, Vicente Andrés

**Affiliations:** 1 Centro Nacional de Investigaciones Cardiovasculares Carlos III (CNIC), Madrid, Spain.; 2 CIBER de Enfermedades Cardiovasculares (CIBERCV), Madrid, Spain.; 3 Instituto de Ciencia de Materiales de Madrid (ICMM) and; 4 Centro de Investigaciones Biológicas Margarita Salas (CIB), Consejo Superior de Investigaciones Científicas (CSIC), Madrid, Spain.

**Keywords:** Aging, Vascular biology, Atherosclerosis, Cardiovascular disease, Endothelial cells

## Abstract

Hutchinson-Gilford progeria syndrome (HGPS) is an extremely rare disease caused by the expression of progerin, an aberrant protein produced by a point mutation in the *LMNA* gene. HGPS patients show accelerated aging and die prematurely mainly from complications of atherosclerosis such as myocardial infarction, heart failure, or stroke. However, the mechanisms underlying HGPS vascular pathology remain ill-defined. We used single-cell RNA sequencing to characterize the aorta in progerin-expressing *Lmna^G609G/G609G^* mice and wild-type controls, with a special focus on endothelial cells (ECs). HGPS ECs showed gene expression changes associated with extracellular matrix alterations, increased leukocyte extravasation, and activation of the yes-associated protein 1/transcriptional activator with PDZ-binding domain (YAP/TAZ) mechanosensing pathway, all validated by different techniques. Atomic force microscopy experiments demonstrated stiffer subendothelial extracellular matrix in progeroid aortae, and ultrasound assessment of live HGPS mice revealed disturbed aortic blood flow, both key inducers of the YAP/TAZ pathway in ECs. YAP/TAZ inhibition with verteporfin reduced leukocyte accumulation in the aortic intimal layer and decreased atherosclerosis burden in progeroid mice. Our findings identify endothelial YAP/TAZ signaling as a key mechanism of HGPS vascular disease and open a new avenue for the development of YAP/TAZ-targeting drugs to ameliorate progerin-induced atherosclerosis.

## Abstract 2

The YAP/TAZ mechanosensing pathway is activated in aortic endothelial cells from progeroid mice, and its pharmacological inhibition ameliorates intimal leukocyte accumulation and atherosclerosis burden.

## Graphical abstract

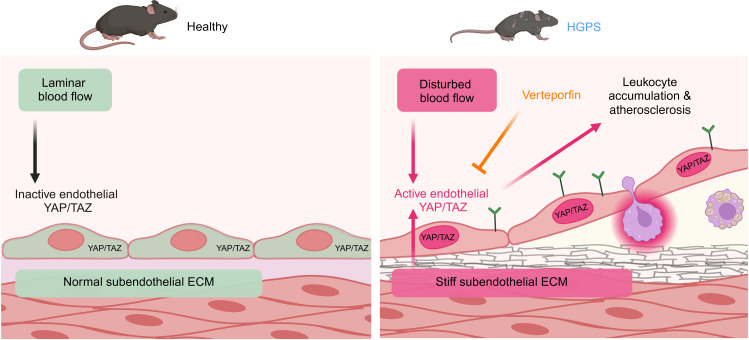

## Introduction

Hutchinson-Gilford progeria syndrome (HGPS) is an ultrarare genetic disease (prevalence of 1 in 20 million people) that causes accelerated aging and premature death at a mean age of 14.6 years, mainly due to heart failure, myocardial infarction, or stroke (1). HGPS is caused by a heterozygous de novo point mutation in the *LMNA* gene, which encodes the nuclear A-type lamins (2, 3). In normal cells, alternative splicing of *LMNA* transcripts results in 2 main protein variants, lamin A and lamin C. Most HGPS patients carry the synonymous c.1824C>T (p.Gly608Gly) *LMNA* mutation, which creates an aberrant splicing site in exon 11, resulting in deletion of 150 nucleotides and the synthesis of a truncated variant of lamin A called progerin. Progerin expression causes multiple structural and functional alterations in cells that affect signal transduction, gene transcription, and chromatin organization, among other processes, eventually causing cellular senescence and death (4).

HGPS patients seem normal at birth but progressively develop growth failure, alopecia, dermal and bone abnormalities, joint contractures, and lipodystrophy (1). The most clinically relevant features of HGPS patients are generalized atherosclerosis and cardiac dysfunction, which in most cases develop in the absence of “classical” cardiovascular risk factors. HGPS-associated atherosclerosis is accompanied by other pathological vascular changes such as severe depletion of medial vascular smooth muscle cells (VSMCs), excessive extracellular matrix (ECM) deposition, calcification, increased vessel stiffness, and impaired vascular tone (4). The HGPS vascular phenotype is likely a consequence of alterations to the heterogeneous cellular composition of the vessel wall and dysregulation of signaling pathways acting on diverse vascular cell types. However, the precise role of distinct cell subtypes in HGPS-associated vascular defects remains ill-defined.

Endothelial cells (ECs) play essential roles in cardiovascular homeostasis, modulating cell survival, angiogenesis, vascular tone, hemostasis, vessel permeability, and inflammation (5). ECs show pronounced heterogeneity between organs and even within the same tissue, and this variability is partly due to local molecular, cellular, and biophysical cues that tailor ECs to fulfill specific functions depending on their location (6). In the arteries, EC function and homeostasis are crucially regulated by alterations to mechanical factors such as ECM stiffness and fluid shear stress (7, 8). Although endothelial dysfunction is a well-known hallmark of atherosclerosis and vascular aging in the general population, the endothelial alterations in the arteries of HGPS mouse models and their impact on other vascular cell types remain poorly characterized. Unraveling the potential implication of ECs in HGPS-associated vascular pathology is therefore of great clinical interest, since it could uncover new therapeutic targets and pave the way to drug development.

Over the past decade, single-cell RNA sequencing (scRNA-Seq) has emerged as a powerful tool to characterize cellular heterogeneity in complex tissues and pathological conditions. Here, we used scRNA-Seq to identify cell type–specific transcriptome alterations in the aortae of progerin-expressing mice and to identify mechanisms that modulate the molecular crosstalk and functional networks among different vascular cell types, with special emphasis on the activation of the yes-associated protein 1/transcriptional activator with PDZ-binding domain (YAP/TAZ) mechanosensing pathway in ECs and its pathophysiological consequences.

## Results

### scRNA-Seq analysis of wild-type and progeroid mouse aorta.

**Figure 1:**
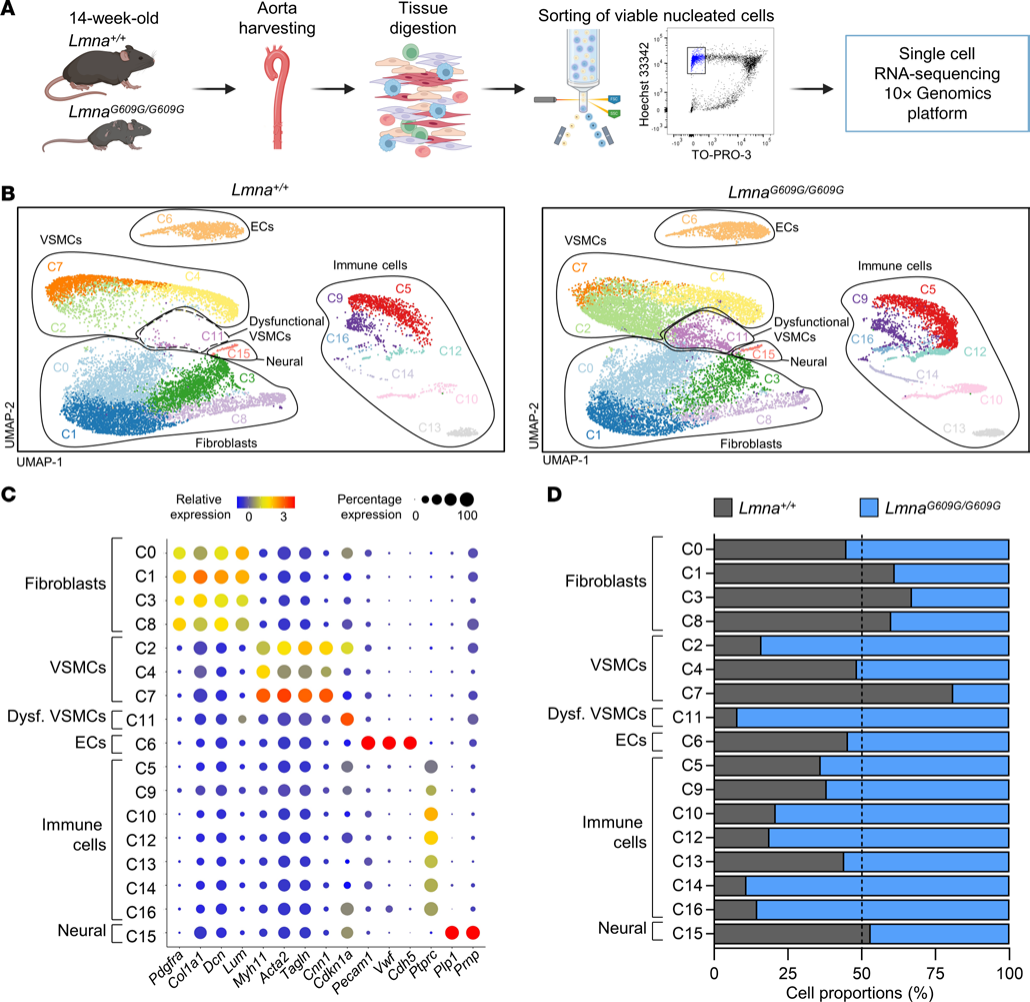
Single-cell RNA-Seq analysis of the aorta in *Lmna^+/+^* and *Lmna^G609G/G609G^* mice. (**A**) Experimental approach. (**B**) Uniform manifold approximation and projection (UMAP) representation of scRNA-Seq data, showing cell clusters and identified cell types. (**C**) Relative expression and percentage of expression of cell type–specific markers in each cluster. (**D**) Relative abundance of each cluster in control and progeroid mice. The dashed line indicates 50% proportion (i.e., same number of sequenced cells in both genotypes for each cluster). Dysf., dysfunctional.

We performed scRNA-Seq to characterize the transcriptional profile of individual cells in the aortae of 14-week-old homozygous *Lmna^G609G/G609G^* mice (9) and wild-type controls (*Lmna^+/+^*). *Lmna^G609G/G609G^* mice recapitulate many HGPS features (e.g., arterial VSMC depletion, ECM accumulation in the aortic media, and increased vessel stiffness) and die prematurely at an average age of about 15 weeks (4, 9). Unlike hypercholesterolemic *Apoe^–/–^ Lmna^G609G/G609G^* mice, *Lmna^G609G/G609G^* mice do not develop atherosclerosis, probably because of their very low levels of circulating pro-atherogenic lipoproteins (10). We chose this atherosclerosis-free model with the aim of finding upstream molecular cues that could be a cause, rather than a consequence, of HGPS-associated atherosclerosis. Aortae were enzymatically digested, and viable nucleated cells (TO-PRO-3^−^Hoechst 33342^+^) were isolated by cell sorting as previously described for other tissues (11) and sequenced using the Chromium 10x Genomics platform (Figure 1A). After removal of predicted doublets and low-quality cells, 34,152 cells were analyzed, with a median of 2,391 detected genes per cell (Supplemental Figure 1A; supplemental material available online with this article; https://doi.org/10.1172/JCI173448DS1). Unsupervised clustering based on gene expression generated 17 clusters (C0–C16) (Figure 1, B and C, and Supplemental Table 1), which were highly similar between biological replicates (Figure 1B, Supplemental Figure 1B, Supplemental Figure 2A, and Supplemental Table 1). Based on the expression of well-known specific markers, clusters were identified as fibroblasts (45.97%), VSMCs (28.21%), immune cells (18.27%), ECs (7.07%), and neural-related cells (0.48%) (Figure 1C). This analysis also detected a cluster of dysfunctional VSMCs highly abundant in *Lmna^G609G/G609G^* aortae but barely detectable in control aortae and characterized by decreased expression of VSMC markers and increased expression of the senescence marker *Cdkn1a* (see Supplemental Results and Supplemental Discussion). The relative abundance of the main cell types identified by scRNA-Seq was very similar to that obtained by flow cytometry analysis of aortic samples subjected to the same enzymatic dispersion protocol (Supplemental Figure 2, B and C) and revealed marked between-genotype differences in the proportion of cells within some clusters (Figure 1D). These results suggest pathological changes in the *Lmna^G609G/G609G^* aorta that modify the complex cellular composition of the vascular wall.

The focus of the present work is the study of ECs, which is described in the following sections. A detailed bioinformatics analysis of fibroblast, VSMC, and immune cell subtypes by unsupervised reclustering of each cell type is included in the online supplement (Supplemental Results, Supplemental Discussion, Supplemental Figures 3–7, and Supplemental Tables 2–13).

### Endothelial heterogeneity in mouse aorta.

**Figure 2:**
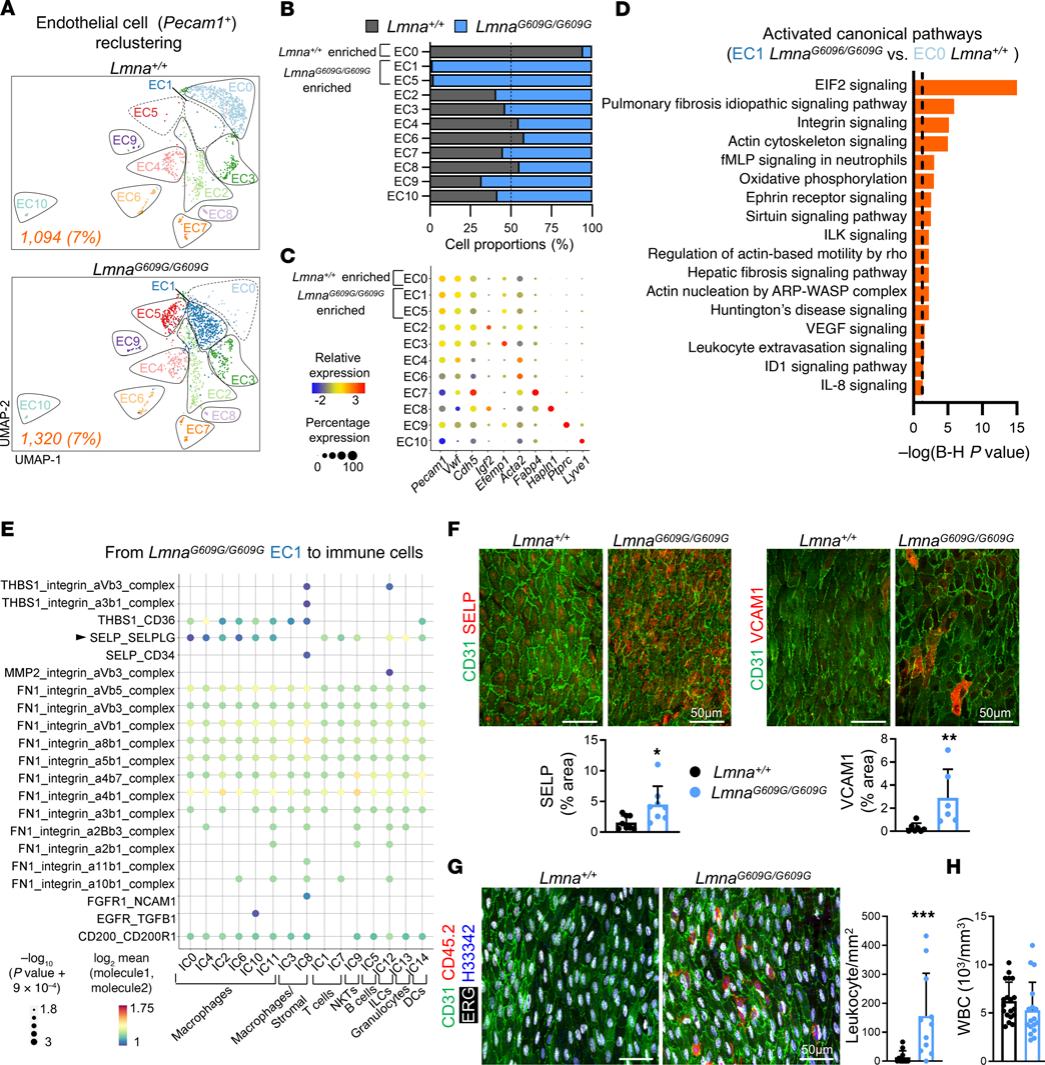
Characterization of aortic endothelial cells in *Lmna^+/+^* and *Lmna^G609G/G609G^* mice. (**A**) UMAP plot of reclustered ECs, showing distinct EC subpopulations. Absolute numbers and percentages of sequenced cells are indicated for each genotype (bottom left corner). (**B**) Relative abundance of EC subclusters in each genotype. The dashed line indicates 50% proportion (i.e., same number of sequenced cells in both genotypes for each subcluster). (**C**) Relative levels and percentage of expression of pan-endothelial markers (*Pecam1*, *Vwf*, and *Cdh5*) and selected genes with specifically increased expression in different EC subclusters. (**D**) Ingenuity Pathway Analysis of activated canonical pathways in *Lmna^G609G/G609G^*-enriched EC1 compared with *Lmna^+/+^*-enriched EC0. Pathways were filtered according to activation score (*z* score > 0) and significance (Benjamini-Hochberg *P* < 0.05). The dashed line indicates Benjamini-Hochberg *P* = 0.05. (**E**) CellPhoneDB prediction of selected ligand-receptor interactions between *Lmna^G609G/G609G^* EC1 and immune cells. SELP-SELPLG interaction is indicated with an arrowhead. (**F**) Representative en face immunofluorescence images of thoracic aortae showing ECs (CD31, green), SELP (left images, red), or VCAM1 (right images, red), and percentage of area positive for SELP (*n* = 8) or VCAM1 (*n* = 6–7). Mean value for each mouse was determined by averaging of values from 3 fields. (**G**) Representative en face immunofluorescence images of thoracic aortae showing ECs (CD31, green), EC nuclei (ERG, white), leukocytes (CD45.2, red), and nuclei (Hoechst 33342, blue) and quantification of intimal leukocytes. Mean values for each mouse (*n* = 11) were determined by averaging of the number of leukocytes present in 3 fields. (**H**) Circulating white blood cell counts (*n* = 17–19). Data are presented as mean + SD. Statistical analysis was performed by permutation test in **E** and Mann-Whitney test in **F**–**H**. Scale bars: 50 μm. *P < 0.05, **P < 0.01, ***P < 0.001. fMLP, *N*-formyl-methionyl-leucyl-phenylalanine; H33342, Hoechst 33342; ILCs, innate lymphod cells; ILK, integrin linked kinase; UMAP, uniform manifold approximation and projection; WBC, white blood cells.

Unsupervised reclustering of 2,414 *Pecam1* (CD31)-expressing ECs generated 11 subclusters (EC0–EC10) (Figure 2A, Supplemental Figure 8A, and Supplemental Table 14). These subclusters included specialized aortic EC subtypes present in both *Lmna^+/+^* and *Lmna^G609G/G609G^* mice and others that were highly specific for each genotype (Figure 2, A–C, and Supplemental Figure 8A). The EC5 subcluster was almost exclusive for *Lmna^G609G/G609G^* mice but was not analyzed further because it contained very few cells in one of the *Lmna^G609G/G609G^* biological replicates (Supplemental Figure 1E). To gain insight into aortic endothelial heterogeneity, we analyzed our data using gene expression scores to detect proliferating cells and to identify lymphatic, capillary, and arterial ECs (see Supplemental Methods). This analysis showed no enrichment of proliferating cells in any endothelial subcluster, and identified EC10 as lymphatic ECs (e.g., *Flt4*), EC7 as capillary ECs (e.g., *Cd36*, *Cd300lg*, *Kdr*, *Rgcc*, *Car4*) probably from the adventitia or contaminating perivascular adipose tissue (12), and the rest of the endothelial subclusters as arterial ECs (e.g., *Clu*, *Ace*, *Gja4*, *Htra1*), most likely luminal aortic ECs (Supplemental Figure 8, B and C). To validate and localize the most abundant arterial subclusters present in both *Lmna^+/+^* and *Lmna^G609G/G609G^* mice (Figure 2, A and B), we carried out en face immunostaining assays on aortic arch and thoracic aorta to identify EC2 (*Ly6a^hi^Aqp1^−^*), EC3 (*Aqp1^+^*), EC4 (*Acta2^hi^Lrp1^−^*), and EC6 (*Lrp1^+^*) as defined in our scRNA-Seq analysis (Supplemental Figure 8D). EC2 was significantly enriched in the proximity of thoracic aorta bifurcations and almost absent in the aortic arch, EC3 showed a clear preferential localization at the aortic arch and very scarce presence in the thoracic aorta, and EC4 and EC6 were enriched at regions of the thoracic aorta devoid of bifurcations, the former without reaching statistical significance (Supplemental Figures 9 and 10). These results are consistent with the presence of a highly heterogeneous population of mouse luminal aortic ECs, thus validating our scRNA-Seq data.

### Alterations in progeroid aortic ECs are consistent with endothelial stress, fibrosis, ECM cellular sensing, and leukocyte recruitment.

EC0 was the most abundant aortic endothelial subcluster in control *Lmna^+/+^* mice, but was almost absent from *Lmna^G609G/G609G^* animals; conversely, EC1 was highly abundant in *Lmna^G609G/G609G^* mice but barely detectable in control aortae (Figure 2, A and B). Bioinformatics analysis of differentially expressed genes (Supplemental Table 15) revealed significant activation of several pathways in *Lmna^G609G/G609G^*-specific EC1 versus *Lmna^+/+^*-specific EC0, including cellular stress–associated EIF2 signaling, VEGF and ephrin receptor signaling, fibrosis-related pathways, and ECM sensing mechanisms involving integrins, integrin-linked kinase signaling, and the actin cytoskeleton (Figure 2D and Supplemental Table 16). *Lmna^G609G/G609G^*-specific EC1 was also enriched in immune-related pathways such as IL-8 signaling and leukocyte extravasation.

These pathways are functionally interconnected, creating a complex network that governs EC function, vascular integrity, and response to injury or inflammation. Molecular and biomechanical cues provided by ECM factors and vessel fibrosis can induce outside-inside signaling mediated by integrins and integrin-linked kinase to promote cytoskeletal rearrangements, morphological alterations, and increased cellular permeability of ECs, all of which are also regulated by VEGF and ephrin receptor signaling. These pathways, together with the action of proinflammatory factors such as IL-8, may lead to endothelial activation, leukocyte extravasation, and inflammation that can in turn promote vascular fibrosis, therefore establishing a detrimental feedback loop.

These findings in ECs, together with the scRNA-Seq results from immune cells showing increased immune cell content in the *Lmna^G609G/G609G^* aorta (Supplemental Figure 6, A and B), strongly suggest that changes in EC gene expression are key contributors to leukocyte adhesion and extravasation in the HGPS mouse aorta. We therefore performed CellPhoneDB bioinformatics analysis to identify potential EC–immune cell interactions mediated by ligand-receptor complexes involved in leukocyte extravasation. We analyzed interactions in which the ligand and receptor were both located at the plasma membrane and where at least one of them was upregulated in *Lmna^G609G/G609G^*-specific EC1 relative to *Lmna^+/+^*-specific EC0. This analysis revealed potential interactions mediated by ECM factors (FN1), ECM regulators (MMP2), immunoregulatory factors (THBS1 and CD200), and integrins (Figure 2E), in good agreement with our analysis of signaling pathways altered in aortic ECs from progeroid mice (Figure 2D). Moreover, CellPhoneDB identified interactions between EC1 and macrophages, T cells, innate lymphoid cells, granulocytes, and dendritic cells involving the adhesion molecule P-selectin (encoded by *Selp*) and its primary ligand P-selectin glycoprotein ligand-1 (PSGL-1, encoded by *Selplg*) (Figure 2E). P-selectin is a plasma membrane protein expressed on activated ECs that plays an essential role in leukocyte recruitment to the inflamed vessel wall (13), and its expression was upregulated in *Lmna^G609G/G609G^* EC1 and EC3 (Supplemental Figure 11A and Supplemental Table 15). En face immunostaining assays on thoracic aorta showed increased expression of both P-selectin and VCAM-1, another marker of endothelial activation, in luminal ECs from *Lmna^G609G/G609G^* mice (Figure 2F).

The above findings suggest EC-mediated increased leukocyte recruitment in HGPS aortae. Consistent with this idea, en face immunofluorescence assays demonstrated significantly higher numbers of CD45^+^ERG^−^ leukocytes in the intimal layer of the thoracic aorta in progeroid *Lmna^G609G/G609G^* mice compared with wild-type controls (Figure 2G and Supplemental Figure 11B), despite similar numbers of total circulating leukocytes in both genotypes (Figure 2H). To further characterize the localization and kinetics of aortic intimal leukocyte accumulation in *Lmna^G609G/G609G^* mice, we carried out en face immunofluorescence assays on aortic arch, thoracic aorta, and abdominal aorta from 8- and 14-week-old animals. Whereas no difference in aortic leukocyte accumulation was distinguishable between control and progeroid mice at 8 weeks of age (Supplemental Figure 11C), all aortic regions showed significantly higher numbers of leukocytes in 14-week-old *Lmna^G609G/G609G^* mice (Supplemental Figure 11D). These results demonstrate that increased expression of endothelial activation markers in *Lmna^G609G/G609G^* mice correlates with age-dependent and generalized leukocyte accumulation in the aortic intima.

### Activation of the YAP/TAZ mechanosensing pathway in progeroid aortic ECs.

**Figure 3:**
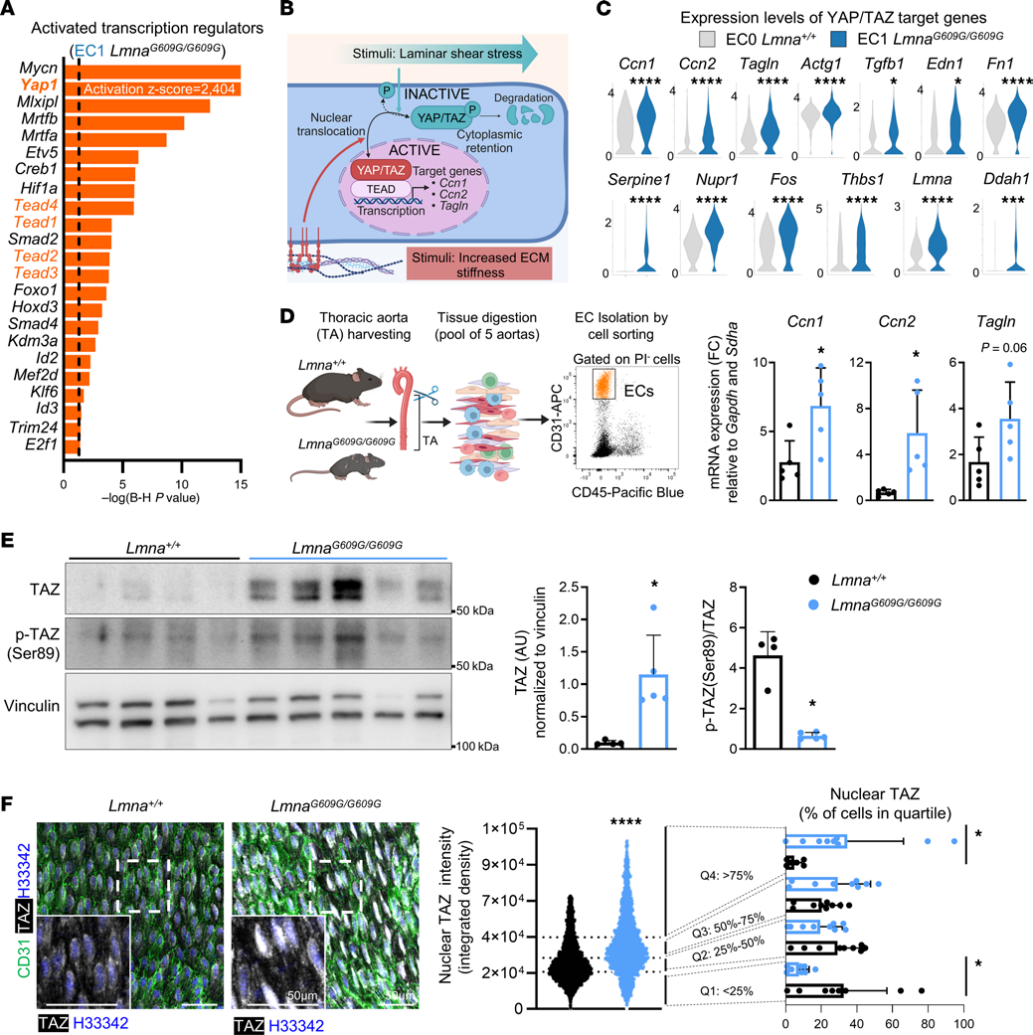
YAP/TAZ signaling is activated in aortic endothelial cells from *Lmna^G609G/G609G^* mice. (**A**) Activated upstream transcriptional regulators in *Lmna^G609G/G609G^*-enriched EC1, predicted by Ingenuity Pathway Analysis of scRNA-Seq data (*z* score > 2, Benjamini-Hochberg *P* < 0.05). The dashed line indicates Benjamini-Hochberg *P* = 0.05. (**B**) Schematic representation of YAP/TAZ pathway regulation. (**C**) scRNA-Seq–determined expression of YAP/TAZ target genes in *Lmna^+/+^*-enriched EC0 and *Lmna^G609G/G609G^*-enriched EC1. (**D**) Protocol outline and gating strategy for the isolation of viable aortic ECs from *Lmna^+/+^* and *Lmna^G609G/G609G^* mice by cell sorting (PI^–^CD31^+^CD45^–^ cells). Graphs show the RT-qPCR–determined expression of canonical YAP/TAZ target genes (*n* = 5). Each dot represents a pool of ECs from 5 thoracic aortae. (**E**) Western blots showing total TAZ, phosphorylated (inactive) TAZ [p-TAZ(Ser89)], and vinculin levels in thoracic aortae from *Lmna^+/+^* and *Lmna^G609G/G609G^* mice (*n* = 4–5). Graphs show the quantification of total TAZ expression normalized to vinculin and the p-TAZ(Ser89)/total TAZ ratio. (**F**) Representative en face immunofluorescence images of thoracic aortae showing ECs (CD31, green), TAZ (white), and nuclei (Hoechst 33342, blue). The violin plot represents nuclear TAZ intensity values in thoracic ECs (2,762–3,074 cells per genotype), and the bar graph shows the percentage of cells in each quartile per mouse (*n* = 9). Boxed areas shown at higher magnification. Data are shown as mean + SD. Statistical analysis was performed by MAST test in **C**, unpaired 2-tailed Student’s *t* test in **D** and **F** (right, Q2–Q4), and Mann-Whitney test in **E** and **F** (left, right Q1). Outliers assessed by ROUT test in **F** were excluded for analysis. Scale bars: 50 μm. *P < 0.05, ***P < 0.001, ****P < 0.0001. FC, fold change; PI, propidium iodide; Q, quartile.

To understand the regulatory cascades that drive gene expression changes in progeroid aortic ECs, we performed a bioinformatics analysis to identify potential transcriptional regulators activated in the *Lmna^G609G/G609G^*-specific EC1 subcluster. Predicted upstream regulators in these cells included the mechanosensitive transcription regulators YAP1, also known as YAP, and transcriptional enhanced associate domain (TEAD) 1–4 (Figure 3A and Supplemental Table 17). YAP and its homolog TAZ are regulated by several physical cues, including ECM stiffness and shear stress. In the absence of activating stimuli, phosphorylated YAP/TAZ remain sequestered in the cytoplasm and eventually undergo ubiquitin-mediated proteasomal degradation. YAP/TAZ activation involves their dephosphorylation and translocation to the nucleus, where they interact with TEAD transcription factors to regulate gene expression (14) (Figure 3B).

Analysis of the EC scRNA-Seq data revealed the upregulation of multiple canonical YAP/TAZ target genes in the *Lmna^G609G/G609G^*-specific EC1 cluster, including *Ccn1* (encoding CYR61), *Ccn2* (encoding CTGF), and *Tagln* (Figure 3C), and these findings were confirmed by reverse transcription-quantitative PCR (RT-qPCR) assays in aortic ECs isolated by cell sorting (Figure 3D). Moreover, immunofluorescence analysis of aortic cross sections showed higher expression of the YAP/TAZ targets CCN2 and FN1 in *Lmna^G609G/G609G^* mice than in controls, both in the region closer to the luminal ECs (first 10 μm) and in the rest of the medial aorta (Supplemental Figure 12, A and B). We also examined TAZ protein content because ECs are known to predominantly express TAZ over YAP (15), as reflected in our scRNA-Seq data (Supplemental Figure 12C). Western blot assays revealed higher total TAZ levels and a reduced proportion of inactive, phosphorylated TAZ [p-TAZ(Ser89)] in progeroid thoracic aortae (Figure 3E), which also exhibited increased TAZ levels in EC nuclei (Figure 3F). Collectively, our bioinformatics analysis and ex vivo experiments underscore YAP/TAZ pathway activation in aortic ECs from *Lmna^G609G/G609G^* mice.

### Increased stiffness of the subendothelial ECM and collagen accumulation in progeroid mouse aorta.

**Figure 4:**
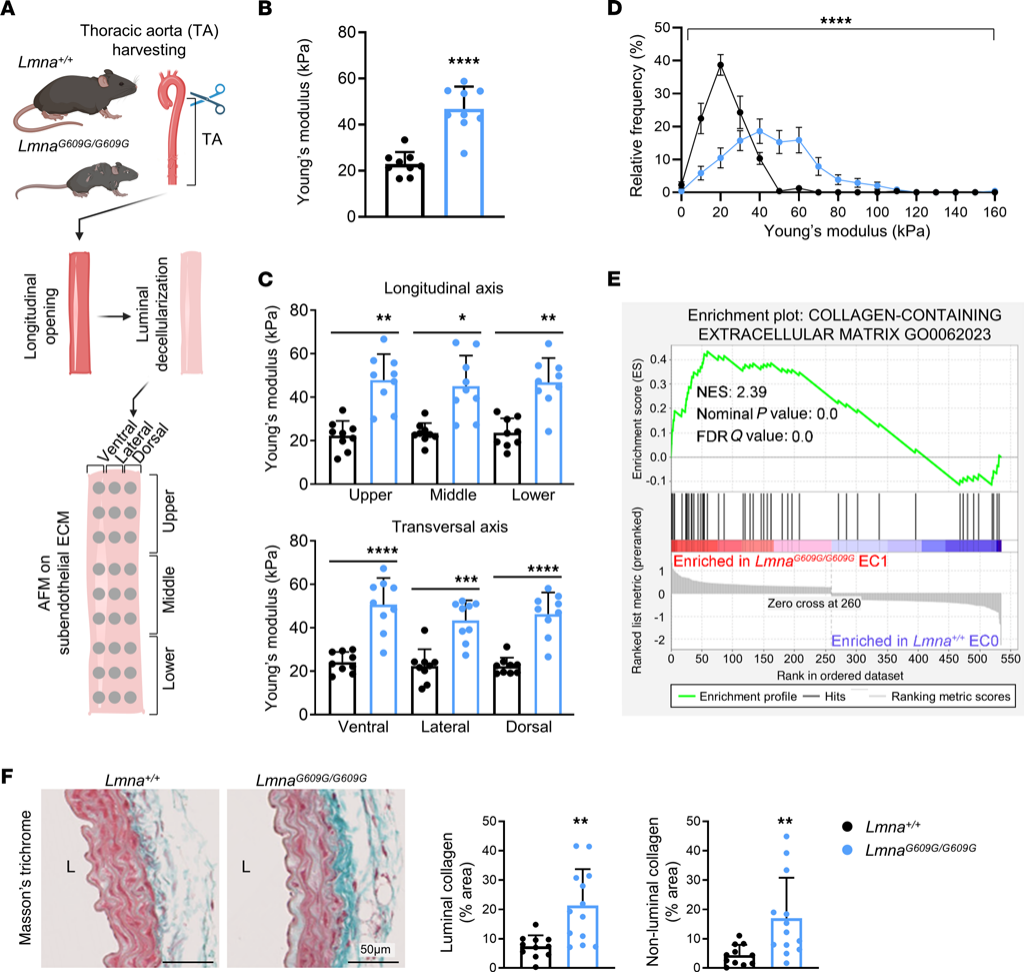
Increased stiffness and collagen accumulation in the aortic subendothelial ECM from *Lmna^G609G/G609G^* mice. (**A**) Workflow for atomic force microscopy analysis of subendothelial ECM in decellularized thoracic aortae. (**B**) Quantification of the Young’s modulus determined by atomic force microscopy (*n* = 9) to estimate aortic subendothelial ECM stiffness (average of the different analyzed regions). (**C**) Zone-dependent analysis of Young’s modulus (*n* = 9). (**D**) Frequency distribution of Young’s modulus values (*n* = 9). (**E**) Gene set enrichment analysis (GSEA) of scRNA-Seq data performed on *Lmna^G609G/G609G^*-enriched EC1 compared with *Lmna^+/+^*-enriched EC0. (**F**) Masson’s trichrome staining of thoracic aorta. Graphs show the quantification of collagen area percentage in the luminal region of the aorta (first 10 μm from the lumen) and in the remaining medial aorta (non-luminal) (*n* = 11–13). Data are shown as mean + SD. Statistical analysis was performed using unpaired 2-tailed Student’s *t* test in **B** and **F**, Mann-Whitney test in **F**, 1-way ANOVA or Kruskal-Wallis tests in **C**, 2-way ANOVA in **D**, and GSEA-calculated nominal *P* value and FDR in **E**. Scale bars: 50 μm. *P < 0.05, **P < 0.01, ***P < 0.001, ****P < 0.0001. AFM, atomic force microscopy; FDR, false discovery rate; L, lumen; NES, normalized enrichment score.

We next sought to investigate potential causal factors for endothelial YAP/TAZ activation in the progeroid aorta. It is well established that increased ECM stiffness induces the YAP/TAZ pathway (16), and previous ex vivo myography studies revealed markedly increased arterial stiffness in *Lmna^G609G/G609G^* mice (17, 18). However, myography quantifies the combined stiffness of the intimal, medial, and adventitial arterial layers. To directly assess the stiffness of the aortic subendothelial ECM, we performed atomic force microscopy experiments on the luminal side of mouse thoracic aortae after removing ECs by chemical decellularization (Figure 4A and Supplemental Figure 13). These studies revealed increased subendothelial ECM stiffness in all analyzed zones in *Lmna^G609G/G609G^* mouse aorta, as shown by a Young’s modulus significantly higher than in controls (Figure 4, B and C). Moreover, a wider stiffness range in decellularized *Lmna^G609G/G609G^* aorta indicated increased heterogeneity in the mechanical properties of the aortic subendothelial ECM in progeroid mice (Figure 4D).

Previous studies have shown increased accumulation of collagen and/or collagen–cross-linking enzymes in arteries from HGPS patients (19) and the *Lmna^G609G/G609G^* HGPS mouse model (17, 18). Moreover, wire myography studies identified collagen accumulation as an underlying cause of aortic stiffness in progeroid mice (17). We therefore hypothesized that the increased stiffness of the subendothelial ECM in HGPS mice might be caused in part by excessive collagen accumulation beneath aortic ECs. Supporting this idea, the scRNA-Seq data showed enriched expression of genes related to collagen-containing ECM in *Lmna^G609G/G609G^*-specific EC1 compared with *Lmna^+/+^*-specific EC0 (Figure 4E). Moreover, reanalysis of aortic cross sections from a previous study by our group (17) showed increased collagen accumulation in *Lmna^G609G/G609G^* mice compared with controls, both in the region closer to the luminal ECs (first 10 μm) and in the rest of the medial aorta (Figure 4F). Together, these findings demonstrate increased stiffness and collagen accumulation in the progeroid aortic subendothelial ECM.

### Disturbed hemodynamics in the progeroid mouse aorta.

**Figure 5:**
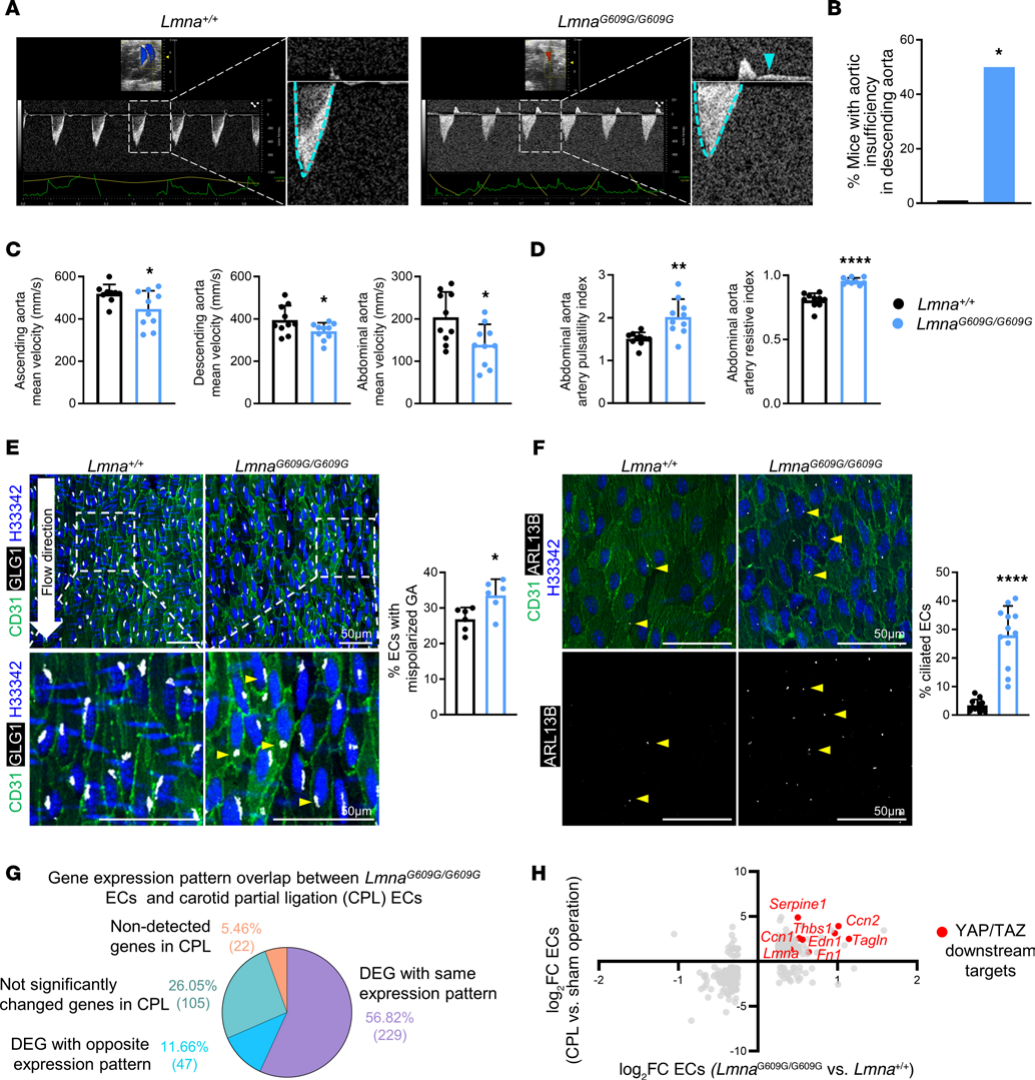
Blood flow alterations in the aorta of *Lmna^G609G/G609G^* mice. (**A**) Representative ultrasound images of descending aorta. Magnified views show pulse-wave Doppler mode graphs, with mean velocity in systole indicated with blue dashed lines (below baseline); in progeroid mice, retrograde flow in diastole is detected as a plateau above baseline (blue arrowhead). (**B**) Percentage of mice with aortic insufficiency (retrograde flow) in the descending aorta: 0 of 10 *Lmna^+/+^* mice; 5 of 10 *Lmna^G609G/G609G^* mice. (**C**) Blood flow mean velocity values in the indicated aortic regions (*n* = 9–10). (**D**) Pulsatility index and resistive index in abdominal aorta (*n* = 10). (**E**) Representative en face immunofluorescence images of thoracic aortae, showing ECs (CD31, green), Golgi apparatus (GLG1, white), and nuclei (Hoechst 33342, blue), and percentage of ECs with mispolarized Golgi apparatus (downstream orientation relative to nuclei, arrowheads) (*n* = 6). Mean values for each mouse were determined by averaging of values from 3 fields. (**F**) Representative en face immunofluorescence images of thoracic aortae, showing ECs (CD31, green), primary cilia (ARL13B, white), and nuclei (Hoechst 33342, blue), and percentage of ciliated ECs (arrowheads) (*n* = 12). Mean values for each mouse were determined by averaging of values from 3 fields. (**G** and **H**) Overlap between gene expression changes in progeroid aortic ECs (C6 cluster in Figure 1, *Lmna^G609G/G609G^* vs. *Lmna^+/+^* mice) and wild-type carotid ECs exposed to disturbed blood flow (carotid partial ligation vs. sham, results reported in ref. 25). Red dots in **H** highlight YAP/TAZ downstream targets significantly upregulated in both conditions. Data in **C**–**F** are shown as mean + SD. Statistical analysis was performed by 2-sided Fisher’s exact test (**B**) and unpaired 2-tailed Student’s *t* test (**C**–**F**). Outliers assessed by ROUT test in **C** and **D** were excluded for analysis. Scale bars: 50 μm. *P < 0.05, **P < 0.01, ****P < 0.0001. CPL, carotid partial ligation; DEG, differentially expressed genes; FC, fold change; GA, Golgi apparatus.

High shear stress in laminar flow areas inhibits the YAP/TAZ pathway, whereas disturbed, oscillatory flow induces YAP/TAZ nuclear localization and activation of downstream signaling (20, 21). Ultrasound assessment revealed retrograde flow in the *Lmna^G609G/G609G^* descending aorta (Figure 5, A and B) and aortic valves (Supplemental Figure 14, A and B), indicating aortic insufficiency. These findings correlated with structural alterations in the aortic valves from progeroid mice, namely loss of cellularity and increased collagen deposition (Supplemental Figure 14C). *Lmna^G609G/G609G^* mice also exhibited reduced mean flow velocity in the ascending, descending, and abdominal aorta (Figure 5, A and C). The artery pulsatility index and resistive index were significantly increased in *Lmna^G609G/G609G^* abdominal aorta (Figure 5D), confirming increased arterial stiffness in progeria.

We next studied the subcellular position of the endothelial Golgi apparatus (GA), which polarizes against flow direction in arterial regions subjected to normal laminar flow and mispolarizes in response to disturbed blood flow (22, 23). En face immunofluorescence assays detected an increased percentage of ECs with a mispolarized GA in the progeroid mouse thoracic aorta (Figure 5E). Progeroid thoracic aortae also contained a higher proportion of ECs bearing primary cilia (Figure 5F); these microtubule-based organelles are usually absent from areas of high shear stress such as the thoracic aorta and are found in areas of disturbed blood flow, such as the aortic arch (24). We also analyzed previously reported bulk RNA-Seq data from carotid artery ECs abnormally exposed in vivo to oscillatory and low flow upon carotid artery partial ligation (CPL) (25). Comparison of CPL-induced endothelial transcriptome alterations with the gene expression changes in progeroid aortic ECs from our scRNA-Seq analysis identified 229 genes (~57%) showing the same pattern of differential expression in both data sets versus the corresponding controls, and this common gene set included multiple YAP/TAZ target genes (Figure 5, G and H). In summary, HGPS mouse aorta showed oscillatory and low blood flow, both of which correlated with the phenotypic and transcriptional alterations induced in ECs by disturbed hemodynamics.

### EC-specific progerin expression is insufficient to trigger YAP/TAZ activation and leukocyte recruitment.

**Figure 6:**
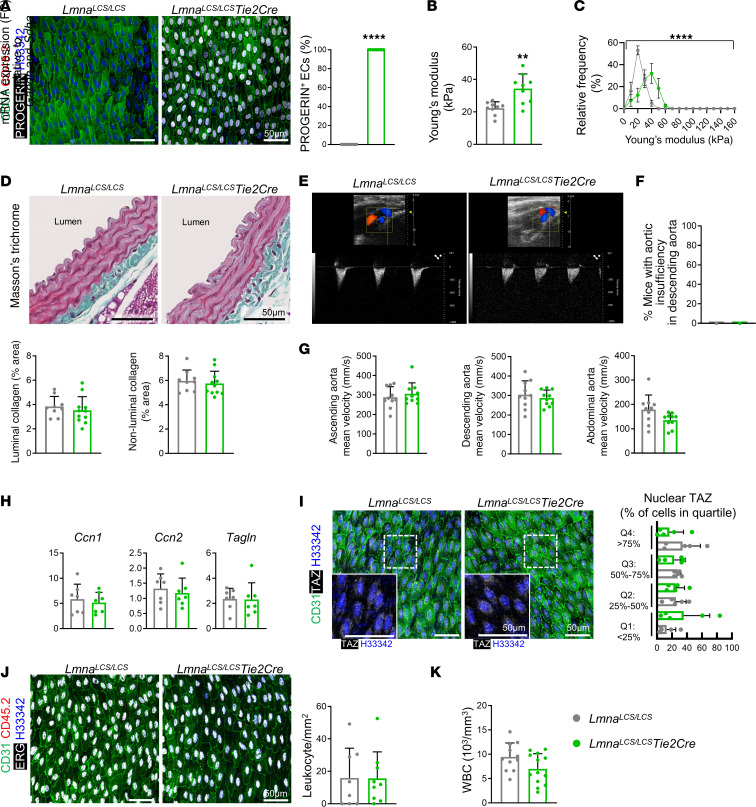
EC-specific progerin expression is not sufficient to trigger YAP/TAZ activation and leukocyte recruitment. (**A**) Representative images of en face immunofluorescence staining in thoracic aorta showing ECs (CD31, green), progerin (white), leukocytes (CD45.2, red), and nuclei (Hoechst 33342, blue), and percentage of progerin^+^ ECs. Mean value per mouse (*n* = 8–9) was determined by averaging of cells from 1 field. (**B**) Young’s modulus determined by atomic force microscopy (*n* = 9). (**C**) Frequency distribution of Young’s modulus (*n* = 9). (**D**) Masson’s trichrome staining of thoracic aorta to quantify percentage of collagen area in the luminal region (first 10 μm from the lumen) and in the remaining medial aorta (non-luminal) (*n* = 9–11). (**E**) Representative ultrasound images of descending aorta. (**F**) Percentage of mice with aortic insufficiency (retrograde flow) in descending aorta (0 of 10 in *Lmna^LCS/LCS^* and *Lmna^LCS/LCS^ Tie2Cre*). (**G**) Blood flow mean velocity at indicated aortic regions (*n* = 10). (**H**) Expression of YAP/TAZ target genes in thoracic aorta ECs quantified by RT-qPCR (*n* = 7). (**I**) Representative en face immunofluorescence images of thoracic aortae showing ECs (CD31, green), TAZ (white), and nuclei (Hoechst 33342, blue). Boxed areas shown at higher magnification. Graph showing the percentage of cells in each quartile per mouse (*n* = 5). (**J**) Representative images of en face immunofluorescence staining showing ECs (CD31, green), EC nuclei (ERG, white), leukocytes (CD45.2, red), and nuclei (Hoechst 33342, blue) in thoracic aorta, and quantification of CD45^+^ cells. Mean value per mouse (*n* = 8–9) was determined by averaging of cells from 9 fields from 3 regions. (**K**) Circulating white blood cell counts (*n* = 11–13). Data in **A**, **B**, **D**, and **G**–**K** are represented as mean + SD. Statistical analysis was performed by Mann-Whitney test (**A** and **G** [ascending aorta]), 2-tailed Student’s *t* test (**B**, **D**, **G** [descending, abdominal aorta], and **H**–**K**), 2-way ANOVA (**C**), and 2-sided Fisher’s exact test (**F**). Scale bars: 50 μm. **P < 0.01, ****P < 0.0001. WBC, white blood cells.

We next evaluated whether progerin expression in ECs is sufficient to induce YAP/TAZ-activating biomechanical stimuli and to trigger endothelial YAP/TAZ activation and leukocyte recruitment into the aortic intima. We used the conditional *Lmna^LCS/LCS^ Tie2Cre* mouse model, in which expression of Cre recombinase is driven by the *Tie2* promoter (also known as *Tek*), resulting in constitutive progerin expression only in ECs and some leukocyte populations (26). Both *Lmna^LCS/LCS^ Tie2Cre* mice and *Lmna^LCS/LCS^* controls (9) ubiquitously lack lamin A and express lamin C, whereas *Lmna^LCS/LCS^ Tie2Cre* mice also express progerin predominantly in ECs. We confirmed efficient and specific endothelial progerin expression in aortae of *Lmna^LCS/LCS^ Tie2Cre* mice by en face and cross-section immunofluorescence assays (Figure 6A and Supplemental Figure 15A).

Atomic force microscopy assays on decellularized aortae (Supplemental Figure 15, B and C) revealed elevated subendothelial ECM stiffness in *Lmna^LCS/LCS^ Tie2Cre* mice compared with *Lmna^LCS/LCS^* controls (Figure 6, B and C), although not as pronounced as in *Lmna^G609G/G609G^* mice with ubiquitous progerin expression (Figure 4B), and the aortic collagen content was similar in both genotypes (Figure 6D). Ultrasound assessment revealed no retrograde flow in the descending aorta and aortic valves in *Lmna^LCS/LCS^ Tie2Cre* mice (Figure 6, E and F, and Supplemental Figure 15D), which also showed normal mean flow velocity in the ascending, descending, and abdominal aorta (Figure 6G). RT-qPCR assays in aortic ECs isolated by cell sorting revealed no differences in the expression of canonical YAP/TAZ targets when comparing *Lmna^LCS/LCS^* and *Lmna^LCS/LCS^ Tie2Cre* cells (Figure 6H). Likewise, immunofluorescence analysis of aortic cross sections revealed no between-genotype differences in the levels of the YAP/TAZ target FN1 (Supplemental Figure 15E). Consistent with these findings, *Lmna^LCS/LCS^ Tie2Cre* mice did not show increased nuclear TAZ levels in luminal aortic ECs, as analyzed by en face immunofluorescence assays (Figure 6I), and they did not exhibit increased accumulation of CD45^+^ERG^−^ leukocytes in the aortic intima (Figure 6J) and had normal circulating leukocyte counts (Figure 6K). Based on these data, we conclude that endothelial YAP/TAZ activation and intimal leukocyte accumulation in the aorta of HGPS mice do not solely rely on endothelial progerin expression and may be indirect, resulting from mechanical cues and/or molecular factors affecting ECs that are induced by progerin expression in non-endothelial cell types.

### YAP/TAZ inhibition reduces endothelial activation and the accumulation of aortic intimal leukocytes in progeroid mice.

**Figure 7:**
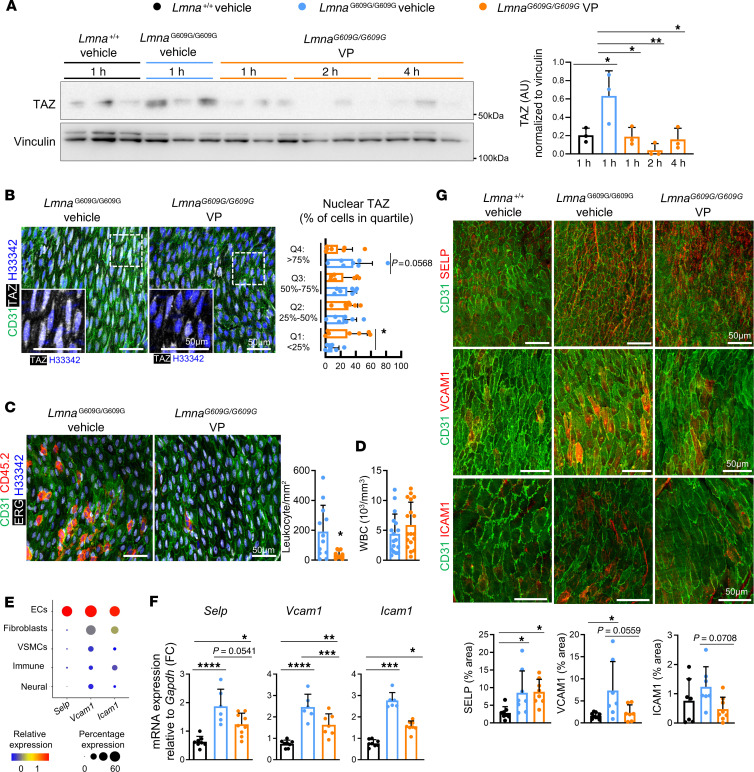
YAP/TAZ inhibition with verteporfin reduces the accumulation of aortic intimal leukocytes in progeroid mice. (**A**) Western blot analysis of thoracic aorta lysates, showing TAZ upregulation in vehicle-treated *Lmna^G6096/G609G^* versus *Lmna^+/+^* mice, and its inhibition after verteporfin treatment (*n* = 3). Vinculin was used as loading control. (**B**) Representative en face immunofluorescence images of thoracic aortae showing ECs (CD31, green), TAZ (white), and nuclei (Hoechst 33342, blue), and quantification of percentage of cells in each quartile per mouse (*n* = 7). Boxed areas shown at higher magnification. (**C**) Representative en face immunofluorescence images of thoracic aortae showing ECs (CD31, green), EC nuclei (ERG, white), leukocytes (CD45.2, red), and nuclei (Hoechst 33342, blue), and quantification of intimal leukocytes. Mean values for individual mice (*n* = 10–12) were determined by averaging of cells from 9 fields from 3 aortic regions. (**D**) Circulating white blood cell counts (*n* = 17–18). (**E**) scRNA-Seq–determined relative expression of *Selp*, *Vcam1*, and *Icam1* in different aortic cell types. (**F**) *Selp*, *Vcam1*, and *Icam1* expression in aortic arches determined by RT-qPCR (*n* = 6–9). (**G**) Representative en face immunofluorescence images of aortae showing ECs (CD31, green) and SELP (top panel, aortic arch, red), VCAM1 (middle panel, thoracic aorta, red), or ICAM1 (bottom panel, thoracic aorta, red). The graph shows the percentage of SELP^+^ (*n* = 8), VCAM1^+^ (*n* = 7–9), and ICAM1^+^ (*n* = 7–8) area. Mean values for individual mice were determined by averaging of values from 3 fields. Data are presented as mean + SD. Statistical analysis was performed using 1-way ANOVA (**A**, **F** [*Selp* and *Vcam1*], and **G**), 1-tailed Student’s *t* test (**B** [Q1, Q2, Q4]), Mann-Whitney test (**B** [Q3]), 2-tailed Student’s *t* test (**C** and **D**), and Kruskal-Wallis test (**F** [*Icam1*]). Outliers assessed by ROUT test in **F** (*Icam1*) were excluded for analysis. Scale bars: 50 μm. *P < 0.05, **P < 0.01, ***P < 0.001, ****P < 0.0001. FC, fold change; VP, verteporfin; WBC, white blood cells.

To explore the therapeutic potential of YAP/TAZ pharmacological inhibition for HGPS-associated vascular disease, we performed preclinical studies with verteporfin, an FDA-approved drug that blocks YAP/TAZ–TEAD complex formation and decreases YAP/TAZ protein levels (27, 28). Verteporfin-treated *Lmna^G609G/G609G^* mice showed significant reductions in aortic TAZ protein expression (Figure 7A) accompanied by a transient, non-significant elevation of the proportion of inactive, phosphorylated TAZ [p-TAZ(Ser89)] (Supplemental Figure 16A). Importantly, verteporfin treatment reduced both nuclear TAZ levels in luminal aortic ECs (Figure 7B) and the number of CD45^+^ERG^−^ leukocytes in the intimal aortic layer (Figure 7C) of *Lmna^G609G/G609G^* mice, without changes in circulating white blood cell counts (Figure 7D). To identify potential mechanisms mediating leukocyte reduction in the aorta of verteporfin-treated HGPS mice, we examined our scRNA-Seq data and previously published studies to search for candidate genes involved in leukocyte extravasation that were upregulated in aortic *Lmna^G609G/G609G^* ECs compared with wild-type controls, and tested whether verteporfin could reduce their expression. The scRNA-Seq analysis revealed EC-specific expression of P-selectin (encoded by *Selp*), VCAM-1 (encoded by *Vcam1*), and ICAM-1 (encoded by *Icam1*) (Figure 7E), well-known markers of endothelial activation that mediate leukocyte-EC adhesion in different inflammatory contexts (13, 29), and previous studies reported VCAM-1 and ICAM-1 upregulation in response to YAP/TAZ activation (20, 30). In agreement with the scRNA-Seq and immunofluorescence experiments indicating aortic endothelial activation in progeroid mice (Figure 2, D–F), RT-qPCR assays showed *Selp*, *Vcam1*, and *Icam1* upregulation in the aortic arch of vehicle-treated *Lmna^G609G/G609G^* mice (Figure 7F). Verteporfin treatment significantly reduced *Vcam1* expression, and the same tendency was observed for *Selp* and *Icam1* (Figure 7F). A similar pattern of verteporfin-mediated decrease in *Vcam1* and *Icam1* expression was detected in aortic luminal ECs from progeroid mice by en face immunofluorescence assays (Figure 7G). Overall, these results indicate that verteporfin reduces the expression of endothelial activation markers in the aorta of *Lmna^G609G/G609G^* mice, which could partially mediate the reduction in intimal leukocyte accumulation observed upon verteporfin treatment.

We next investigated whether verteporfin-mediated decrease in endothelial activation could be due to an amelioration of the aortic structural and blood flow alterations observed in *Lmna^G609G/G609G^* mice. Verteporfin treatment of progeroid mice had no significant effect on the aortic collagen content (Supplemental Figure 16B), on the retrograde blood flow measured at both the descending aorta and the aortic valves (Supplemental Figure 16, C–E), or on blood flow velocity at the ascending, descending, and abdominal aorta (Supplemental Figure 16F). Likewise, verteporfin-treated *Lmna^G609G/G609G^* mice did not show a reduced percentage of aortic luminal ECs with mispolarized GA and cilia (Supplemental Figure 16, G and H), indicating that endothelial features related to disturbed blood flow were not corrected after verteporfin treatment. We therefore speculated that verteporfin-mediated decrease in EC activation was due to a direct inhibitory effect of verteporfin on endothelial YAP/TAZ. To test this hypothesis, we set up an in vitro system to test the effect of verteporfin on human aortic ECs (HAECs) cultured at low density, which was previously reported to increase TAZ levels and to induce the YAP/TAZ pathway (16). As expected, sparse HAECs showed increased nuclear TAZ levels compared with high-density cultures, and verteporfin treatment reduced nuclear TAZ accumulation and decreased the expression of the YAP/TAZ targets *CCN1* and *CCN2* (Supplemental Figure 17, A and B). Moreover, in agreement with our in vivo results (Figure 7F), treatment with verteporfin significantly reduced *SELP*, *VCAM1*, and *ICAM1* expression in sparse HAEC cultures (Supplemental Figure 17B). Because the expression of endothelial activation markers is known to be induced by NF-κB in response to proinflammatory factors (5), we tested the effect of verteporfin and the NF-κB inhibitor Ro106-9920 (31) on HAECs after TNF-α stimulation (Supplemental Figure 17C). *SELP* expression in HAECs was neither induced by TNF-α nor reduced by Ro106-9920 after TNF-α stimulation, whereas verteporfin-treated HAECs showed significantly lower *SELP* expression even in the presence of TNF-α (Supplemental Figure 17D). Conversely, TNF-α strongly induced *VCAM1* and *ICAM1* expression, whose upregulation was inhibited by Ro106-9920 and unaffected by verteporfin treatment (Supplemental Figure 17D). These results show that, at least in our experimental setting, verteporfin treatment reduces endothelial activation in the absence of potent proinflammatory stimuli by directly inhibiting the YAP/TAZ pathway in ECs. Upon acute inflammation, however, NF-κB is the main driver of endothelial activation, and YAP/TAZ inhibition by verteporfin does not reduce NF-κB–mediated gene expression.

### YAP/TAZ inhibition attenuates the atherosclerosis burden in progeroid mice.

**Figure 8:**
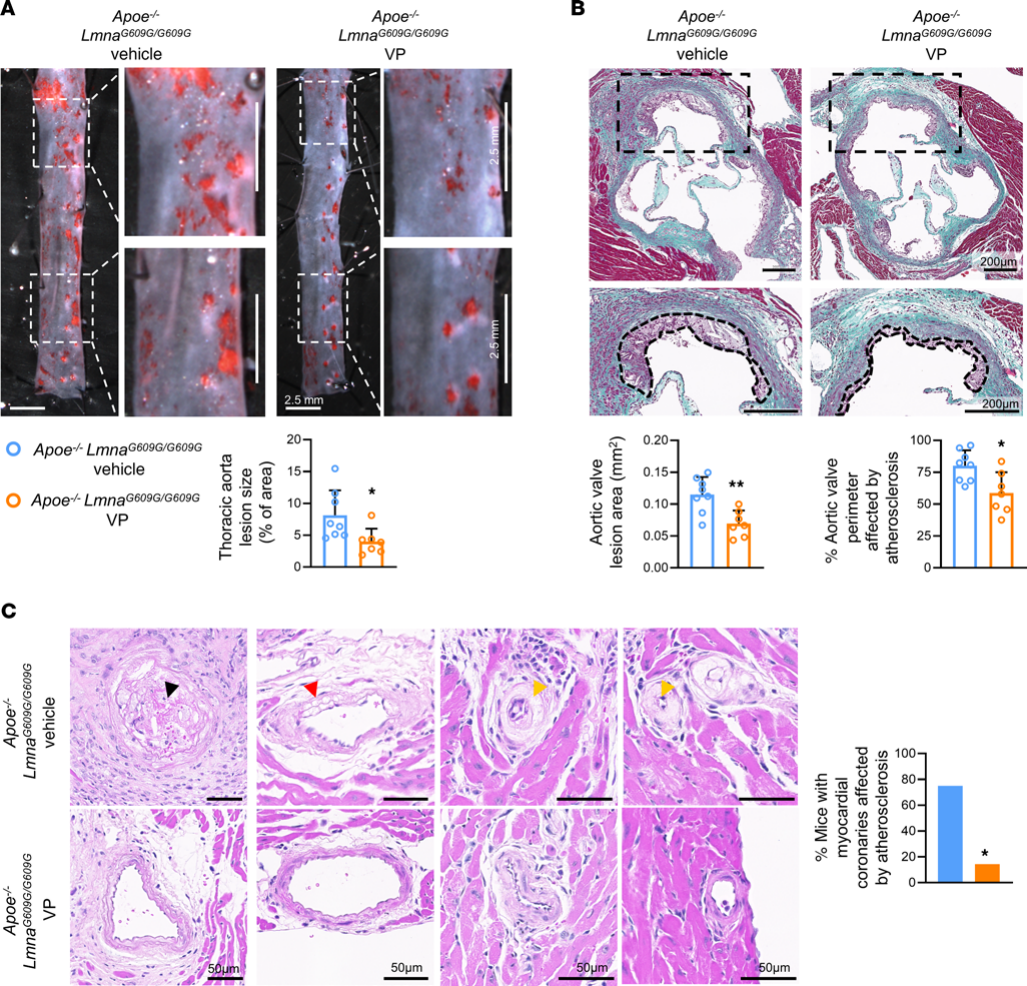
YAP/TAZ inhibition with verteporfin attenuates atherosclerosis burden in progeroid mice. (**A**) Representative images of en face Oil Red O staining of thoracic aortae from vehicle- or verteporfin-treated *Apoe^–/–^ Lmna^G6096/G609G^* mice; the graph shows quantification of atherosclerotic lesion size (*n* = 7–8). Boxed areas are shown at higher magnification. (**B**) Masson’s trichrome staining of the aortic root from vehicle- or verteporfin-treated *Apoe^–/–^ Lmna^G6096/G609G^* mice; the graphs show quantification of plaque area and the percentage of the aortic valve perimeter affected by atherosclerosis (*n* = 7–8). Boxed areas are shown at higher magnification in the bottom images, where atherosclerotic plaques are indicated by dashed lines. Each point represents the mean of 2 aortic root regions per mouse. (**C**) Representative images of H&E staining of myocardial coronary arterioles from vehicle- or verteporfin-treated *Apoe^–/–^ Lmna^G6096/G609G^* mice, and quantification of the percentage of mice presenting at least 1 myocardial vessel (diameter >50 μm) with signs of atherosclerotic disease. Black, red, and yellow arrowheads show examples of atherosclerotic plaque, medial lipid accumulation, and intimal hypertrophy, respectively. *Apoe^–/–^ Lmna^G609G/G609G^* vehicle mice, 6 of 8; *Apoe^–/–^ Lmna^G609G/G609G^* VP mice, 1 of 7. Statistical analysis was carried out using 2-tailed Student’s *t* test (**A** and **B**) and 2-sided Fisher’s exact test (**C**). Scale bars: 2.5 mm (**A**), 200 μm (**B**), and 50 μm (**C**). *P < 0.05, **P < 0.01. VP, verteporfin.

To test the effects of verteporfin on HGPS-associated atherosclerosis, we used fat-fed atheroprone *Apoe^–/–^ Lmna^G609G/G609G^* mice, in which atherosclerosis is aggravated in comparison with progerin-free *Apoe^–/–^* controls expressing wild-type lamin A (10). Compared with the vehicle control group, verteporfin-treated *Apoe^–/–^ Lmna^G609G/G609G^* mice did not exhibit differences in body weight, aortic collagen content, serum cholesterol levels, or circulating white blood cell counts (Supplemental Figure 18, A–D). Verteporfin treatment, however, significantly reduced atherosclerosis burden in the thoracic aorta (Figure 8A) and aortic valves (Figure 8B), as quantified after Oil Red O and Masson’s trichrome staining, respectively. Moreover, as previously reported in HGPS minipigs (32), myocardial coronary arterioles of *Apoe^–/–^ Lmna^G609G/G609G^* mice showed signs of atherosclerotic disease, including plaque buildup, medial lipid accumulation, and intimal thickening, and verteporfin treatment significantly reduced the percentage of mice presenting at least one of these alterations in myocardial vessels with a diameter greater than 50 μm (Figure 8C). Likewise, atherosclerotic alterations in coronary arteries at their origin in the aortic root, a highly atheroprone site, also tended to be reduced after verteporfin treatment (Supplemental Figure 18E). Collectively, these results demonstrate that YAP/TAZ pathway inhibition by verteporfin ameliorates the atherosclerosis burden in HGPS mice.

## Discussion

Our scRNA-Seq analysis provides a comprehensive transcriptional characterization of multiple cell types from the adventitial, medial, and intimal aortic layers of progerin-expressing *Lmna^G609G/G609G^* mice and wild-type controls, allowing us to identify the activation of YAP/TAZ signaling in ECs as a key mechanism of HGPS-related vascular disease. We provide a Supplemental Discussion section including the analysis of fibroblasts, VSMCs, and immune cells. The discussion below is focused on the results obtained from aortic ECs, the main topic of the present study.

The markedly altered gene expression profile of aortic ECs in *Lmna^G609G/G609G^* mice includes activation of pathways associated with leukocyte extravasation, consistent with the increased accumulation of immune cells in the intimal layer of the progeroid mouse aorta. Our scRNA-Seq analysis revealed that *Lmna^G609G/G609G^* mice show signs of endothelial activation in endothelial subclusters from different aortic regions. For example, *Selp* was significantly upregulated in *Aqp1*^−^ EC1 and *Aqp1*^+^ EC3, the latter specifically enriched at the aortic arch, and showed the same tendency in *Ly6a^hi^Aqp1^−^* EC2 and *Lrp1*^+^ EC6, enriched in different regions of the thoracic aorta. Another interesting example is *Sox4*, which has been shown to be induced in aortic ECs from atheroprone mice and in ECs under oscillatory flow (33), and was significantly upregulated in EC1, EC2, and EC3 from progeroid mice (Supplemental Table 15). These observations, together with our gene expression and immunofluorescence assays validating endothelial activation at both the aortic arch and the thoracic aorta, and our data showing intimal leukocyte accumulation in all the analyzed aortic regions (aortic arch, thoracic aorta, and abdominal aorta), indicate that *Lmna^G609G/G609G^* mice develop vascular inflammation throughout the whole aorta.

Upregulation of the inflammatory response has been reported previously in progerin-expressing ECs (34–38); however, those studies were not performed in aortic ECs and did not address the functional impact of the upregulation of proinflammatory signaling on the vasculature in vivo. In the present study, we took a step forward by establishing a link between EC alterations and increased leukocyte recruitment in HGPS aortae and identifying the activation of the mechanosensing transcription regulator YAP/TAZ in progeroid aortic ECs. YAP/TAZ is activated by increased substrate stiffness and disturbed flow, which induce inflammatory responses and atherosclerosis development in non-HGPS mice and cultured cells (7, 25, 39, 40). Moreover, suppression of arterial stiffness results in reduced leukocyte infiltration and attenuated atherosclerosis (41). Our results reveal an association between endothelial YAP/TAZ activation and increased stiffness and altered subendothelial ECM composition in HGPS aortae, together with elevated expression of fibrosis-related genes in aortic ECs.

The accompanying oscillatory blood flow in the descending aorta of HGPS mice is consistent with the aortic valve and mitral regurgitation present in some HGPS patients (42). Previous in vitro studies reported intrinsically impaired mechanotransduction in progerin-expressing ECs (34, 43–46) due to perturbed nucleocytoskeletal coupling, a defective shear stress response, and dysregulation of mechanoresponsive myocardin-related transcription factor A (MRTFA) (47). In agreement with these observations, bioinformatics analysis of our scRNA-Seq data predicted MRTFA as a potential upstream regulator of gene expression changes in HGPS ECs. Therefore, it is conceivable that a combination of EC extrinsic cues (altered mechanical stimuli) and intrinsic cues (impaired mechanotransduction) triggers endothelial activation and contributes to progeria-associated vascular pathology. However, aortic endothelial YAP/TAZ activation and downstream signaling, as well as intimal leukocyte recruitment, were not induced in *Lmna^LCS/LCS^ Tie2Cre* mice with progerin expression mainly restricted to ECs. Interestingly, aortae from *Lmna^LCS/LCS^ Tie2Cre* mice did not show increased collagen and FN1 accumulation or blood flow alterations, but exhibited increased subendothelial stiffness. This indicates that the elevated subendothelial stiffness we observed in *Lmna^G609G/G609G^* mice with ubiquitous progerin expression may not be sufficient to cause YAP/TAZ activation in aortic ECs, although we cannot rule out its possible contribution together with other pathological stimuli.

We conclude that endothelial YAP/TAZ activation and intimal leukocyte accumulation in the progeroid mouse aorta are not induced by progerin-mediated intrinsic effects on ECs, but rather are mainly triggered by environmental cues such as disturbed blood flow, ECM factors, and other molecules induced by progerin expression in non-endothelial cells. Indeed, we have recently shown TGF-β–mediated endothelial-to-mesenchymal transition of aortic ECs in *Apoe^–/–^ Lmna^G609G/G609G^* mice with ubiquitous progerin expression but not in *Apoe^–/–^ Lmna^LCS/LCS^ Cdh5-CreERT2* mice with EC-specific progerin expression (48). Our results therefore strongly suggest that progerin-expressing non-endothelial cell types such as VSMCs (10, 17, 49–52) are key mediators of endothelial and vascular alterations in HGPS.

In non-HGPS ECs, YAP/TAZ signaling is activated in pathophysiological processes, including leukocyte infiltration and atherogenesis (20, 21). Moreover, EC-specific YAP overexpression or expression of constitutively active YAP/TAZ mutants aggravates atherosclerosis in *Apoe^–/–^* mice, and YAP/TAZ activation in cultured human ECs triggers inflammation and increases the expression of adhesion molecules and leukocyte-interacting receptors (20, 21, 30). Conversely, several YAP/TAZ inhibitory strategies have proven effective at decreasing atherosclerosis burden in *Apoe^–/–^* mice, including morpholino oligonucleotides that systemically blunt YAP/TAZ activity, systemic TAZ knockdown with short hairpin RNAs, and EC-specific CRISPR/Cas9–mediated YAP reduction (20, 21). In our analysis, YAP/TAZ in the aortae of progeroid mice was efficiently inhibited by FDA-approved verteporfin, and this treatment was associated with attenuation of aortic leukocyte recruitment and atherosclerosis development. Because verteporfin treatment had no significant effects on the aortic collagen content, the retrograde blood flow, and blood flow velocity, we propose that its beneficial effect on progeroid mice could be at least partially mediated by a direct effect on ECs resulting in the downregulation of leukocyte adhesion molecules, which was supported by our in vitro experiments. A limitation of our approach based on systemic verteporfin delivery is that YAP/TAZ activity is important for physiological functions such as stem cell self-renewal and the maintenance of stem cell phenotype (53), which may limit its translational potential. A reasonable next stage in testing the viability of using verteporfin to treat HGPS would be to design and evaluate strategies to inhibit YAP/TAZ specifically in ECs using EC-targeted delivery systems such as lipid carriers (54) or monocyte membrane-coated nanoparticles (55). Nevertheless, our findings highlight the potential of YAP/TAZ inhibition for the treatment of HGPS-associated atherosclerosis.

## Methods

A detailed description of all experimental and analytical methods is provided as Supplemental Methods.

### Sex as a biological variable.

Our study examined male and female animals, and sex was not considered as a biological variable.

### Mice.

Studies including mice with ubiquitous progerin expression were conducted with 14-week-old *Lmna^G609G/G609G^* mice (9) and age-matched wild-type *Lmna^+/+^* littermates as controls, including also 8-week-old mice of both genotypes where indicated. Atherosclerosis studies were performed with 14-week-old atheroprone progeroid *Apoe^–/–^ Lmna^G609G/G609G^* mice with ubiquitous progerin expression (10). Studies including mice with *Tie2* promoter–driven progerin expression in ECs were carried out with 14-week-old *Lmna^LCS/LCS^ Tie2Cre* mice (obtained by breeding of *Lmna^LCS/LCS^* mice [ref. 9] with the *Tie2Cre* model [ref. 26]) and age-matched *Lmna^LCS/LCS^* littermates as controls. All mouse experiments included balanced numbers of males and females.

### Single-cell RNA-Seq.

Aortae (including aortic arch and thoracic aorta) from *Lmna^+/+^* and *Lmna^G609G/G609G^* mice were harvested, cleaned of perivascular fat, and opened longitudinally. Viable single-cell suspensions were obtained using a previously described protocol with minor modifications (11). We analyzed 2 replicate samples per genotype, and each sample contained pooled cells from the aortae of 2 male and 2 female animals to avoid possible sex-related bias. Cells were loaded onto a Chromium Next GEM Chip G (10x Genomics), and libraries were created using the Next GEM Single Cell 3′ Library preparation kit v3.1 (10x Genomics) and indexed using the Chromium i7 Multiplex kit (10x Genomics). Libraries were paired-end-sequenced in a HiSeq 4000 system (Illumina), and single-cell transcriptomes were obtained using the 10x Genomics Cell Ranger 3.1.0 pipeline and analyzed with the Scater and Seurat R packages. We analyzed a total of 34,152 cells after removing predicted doublets and low-quality cells.

### En face aorta immunostaining and quantitative image analysis.

En face immunofluorescence assays of aortic tissue were performed according to a previously described protocol (56).

### Atomic force microscopy on decellularized aortae.

Thoracic aorta samples were decellularized using a previously described protocol for in vitro applications (57). Atomic force microscopy experiments on subendothelial ECM were performed with a Nanowizard III system (JPK Instruments) mounted on an inverted optical microscope (AXIO Observer D1, Carl Zeiss).

### Mouse ultrasound assessment.

Transthoracic ultrasound assessment was performed using a high-frequency ultrasound system (Vevo 2100, Visualsonics Inc.) equipped with a 30 MHz linear MS400 probe.

### YAP/TAZ inhibition in vivo.

HGPS mice were randomized to receive intraperitoneal injections of either 100 mg/kg verteporfin (SML0534, MilliporeSigma) or 10% DMSO in PBS as vehicle control. The verteporfin dose and administration protocol were selected based on previously published protocols (27, 28). In pilot experiments to test verteporfin efficacy, mice received a single dose of 100 mg/kg verteporfin or vehicle, and aortae were collected 1, 2, or 4 hours after injection. For experiments in *Lmna^G609G/G609G^* mice, animals were injected with 100 mg/kg verteporfin or vehicle every second day over a period of 8 days. For atherosclerosis studies, fat-fed *Apoe^–/–^ Lmna^G609G/G609G^* mice were injected with 100 mg/kg verteporfin or vehicle 3 days per week on alternate weeks for a total of 7 weeks (13 injections).

### YAP/TAZ and NF-κB inhibition in HAEC cultures.

HAECs (ATCC CRL-4052) were used for in vitro studies. Cells were treated with media containing 2 μM Ro106-9920 (557550, Merck), 1 μM verteporfin (SML0534, MilliporeSigma), or vehicle, and stimulated with 5 ng/mL TNF-α (210-TA-005, R&D Systems) or vehicle.

### Statistics.

Quantitative data are presented as the mean + SD unless otherwise stated. All statistical analyses were performed with Prism 8 and 9 (GraphPad). The Shapiro-Wilk test was used to study data distribution. For parametric data with 2 groups, statistical differences were assessed with the unpaired 2-tailed Student’s *t* test, applying Welch’s correction if the groups had unequal variances. For parametric data with 2 groups and a directional hypothesis, we used 1-tailed Student’s *t* test. For non-normally distributed data in experiments with 2 groups, the Mann-Whitney test was used. For parametric data with more than 2 groups and normally distributed populations, we applied 1-way ANOVA followed by post hoc Tukey’s test, with logarithmic transformation of the data when the groups had unequal variances. For non-parametric data with more than 2 groups, we used the Kruskal-Wallis test followed by post hoc Dunn’s test. Outliers were assessed by robust regression and outlier removal (ROUT) test (*Q* = 1%) and excluded for analysis as indicated in the figure legends. For scRNA-Seq data, the MAST method was used. For CellPhoneDB analysis, the permutation test was used. For histogram analysis, we used 2-way ANOVA. For the analysis of contingency data, we used 2-sided Fisher’s exact test. For body weight evolution, we used the mixed-effects model with the Geisser-Greenhouse correction. The significance level was defined as *P < 0.05, **P < 0.01, ***P < 0.001, ****P < 0.0001.

### Study approval.

All animal procedures followed EU Directive 2010/63EU and Recommendation 2007/526/EC, enacted in Spain under Real Decretos 53/2013 and 191/2013. Animal protocol was approved by the Animal Welfare Committee of Centro Nacional de Investigaciones Cardiovasculares Carlos III (Madrid) and the Research Ethics Committee of the Universidad Autónoma de Madrid (Madrid) and authorized by the Animal Protection Area of the Comunidad Autónoma de Madrid (PROEX 105.0/22).

### Data availability.

All data supporting the findings of this study are available within the article and its supplemental information files or upon reasonable request. All scRNA-Seq data were deposited at BioStudies-ArrayExpress (E-MTAB-13678), and the code used for scRNA-Seq analysis was deposited in GitHub (https://github.com/LAB-VA-CNIC/sc-lmnaG609-aorta). Values for all data points in graphs are reported in the Supporting Data Values file.

## Author contributions

A Barettino, IB, and VA designed research studies. A Barettino, CGG, MJAM, PG, CRG, FME, RMC, YB, BD, AQ, A Benguría, and IB conducted experiments and acquired data. A Barettino, CRG, CT, FSC, AD, RG, IB, and VA analyzed data. CT and FSC provided the code for scRNA-Seq analysis. FSC, AD, RG, IB, and VA supervised the experimental and analytical work. A Barettino, IB, and VA wrote the manuscript. IB and VA coordinated the study and acquired funding.

## Supplementary Material

Supplemental data

Unedited blot and gel images

Supplemental table 1

Supplemental table 2

Supplemental table 3

Supplemental table 4

Supplemental table 5

Supplemental table 6

Supplemental table 7

Supplemental table 8

Supplemental table 9

Supplemental table 10

Supplemental table 11

Supplemental table 12

Supplemental table 13

Supplemental table 14

Supplemental table 15

Supplemental table 16

Supplemental table 17

Supplemental table 18

Supporting data values

## Version 1 10/01/2024

In-Press Preview

## Version 2 11/15/2024

Electronic publication

## Version 3 11/25/2024

updated supplemental tables 1,2,9,11,12,14
