# Supplementary material for: Endothelial YAP/TAZ activation promotes atherosclerosis in a mouse model of Hutchinson-Gilford progeria syndrome
Source: J Clin Invest. 2024 Oct 1;134(22):e173448. doi: 10.1172/JCI173448 (PMC11563688; doi:10.1172/JCI173448)
Supplement: Supplemental data [file jci-134-173448-s009.pdf]

SUPPLEMENTAL MATERIAL

Endothelial YAP/TAZ activation promotes atherosclerosis in a mouse model of Hutchinson-Gilford progeria syndrome

Ana Barettino[1,2], Cristina González-Gómez[1,2], Pilar Gonzalo[1,2], María J. Andrés-Manzano[1,2], Carlos R. Guerrero[3], Francisco M. Espinosa[3], Rosa M. Carmona[1], Yaazan Blanco[1], Beatriz Dorado[1,2], Carlos Torroja[1], Fátima Sánchez-Cabo[1], Ana Quintas[1], Alberto Benguría[1], Ana Dopazo[1], Ricardo García[3], Ignacio Benedicto[1,4,*], Vicente Andrés[1,2,*]

[1] Centro Nacional de Investigaciones Cardiovasculares Carlos III (CNIC), Madrid, Spain.
[2] CIBER de Enfermedades Cardiovasculares (CIBERCV), Spain.
[3] Instituto de Ciencia de Materiales de Madrid (ICMM), Consejo Superior de Investigaciones Científicas (CSIC), Madrid, Spain.
[4] Centro de Investigaciones Biológicas Margarita Salas (CIB), Consejo Superior de Investigaciones Científicas (CSIC), Madrid, Spain.

* Co-corresponding authors with equal contribution

Ignacio Benedicto
CIB-CSIC, Ramiro de Maeztu 9, 28040 Madrid (Spain)
Phone: +34 91 837 31 12
E-mail: ignacio.benedicto@cib.csic.es

Vicente Andrés
CNIC, Melchor Fernández Almagro 3, 28029 Madrid (Spain)
Phone: +34 91 453 12 00 (Ext. 1502)
E-mail: vandres@cnic.es

CONTENT

Supplemental Materials and Methods
Supplemental Results
Supplemental Discussion
Supplemental References 58-88
Includes all the references that are cited only in this Supplement. References 1-57 can be found in the main text.
Supplemental Figures S1–S18
Legends for Supplemental Tables 1–18

SUPPLEMENTAL MATERIALS AND METHODS

Mice. Mice were housed in a specific pathogen-free facility in individually ventilated cages with 12 h light/12 h dark cycles at a temperature of 22 ± 2 °C and 50% relative humidity (range 45%–60%). Mice had *ad libitum* access to water and food (D184, SAFE, and Rod18-A, LASQCdiet). All experiments included a balanced number of males and female to avoid possible sex-related bias, and all mice were sacrificed for analysis at 14 weeks of age, unless otherwise stated (8-week-old mice in Supplemental Figure S11C). *Lmna*$^{G609G/G609G}$ mice with ubiquitous progerin expression were previously described (9) and age-matched wild-type *Lmna*$^{+/+}$ littermates were used as controls. Atherosclerosis studies were performed using progeroid atheroprone *Apoe*$^{-/-}$*Lmna*$^{G609G/G609G}$ mice (10), which were fed a high-fat diet (10.7% total fat and 0.75% cholesterol; S9167-E011, Ssniff) for 7 weeks starting at 7 weeks of age and were then sacrificed for further analysis. *Lmna*$^{LCS/LCS}$*Tie2Cre* mice with EC-specific progerin expression were obtained by breeding *Lmna*$^{LCS/LCS}$ mice (9) with *Tie2Cre* mice (26), and age-matched *Lmna*$^{LCS/LCS}$ littermates were used as controls. All experimental mice used in this study were on the C57BL/6J genetic background, and all analyses were carried out by operators blinded to genotype. Mice were euthanized by CO_2 inhalation unless noted otherwise. All animal procedures followed EU Directive 2010/63EU and Recommendation 2007/526/EC, enacted in Spain under Real Decretos 53/2013 and 191/2013. Animal protocols were approved by the local ethics committees and the Animal Protection Area of the Comunidad Autónoma de Madrid (PROEX 105.0/22).

Single-cell RNAseq of mouse aorta

Single cell suspension preparation. *Lmna*$^{+/+}$ and *Lmna*$^{G609G/G609G}$ mice were euthanized and perfused transcardially with 10 ml cold phosphate-buffered saline (PBS) to wash blood from the aorta. Aortas (including aortic arch and thoracic aorta) were harvested, cleaned of perivascular fat, and opened longitudinally in cold DMEM (Sigma) supplemented with antibiotics (100 U ml^{-1} penicillin, 0.1 mg ml^{-1} streptomycin, Sigma), 0.292 mg ml^{-1} L-glutamine (HyClone), and 0.1 mM sodium pyruvate (SH30239.01, HyClone). Each sample contained pooled cells from four aortas per genotype, including two males and two females in each pool to avoid possible sex-related bias, and two replicate samples per genotype were analyzed. Viable single-cell suspensions were obtained using a previously described protocol with minor modifications (11). Aortas were digested at 37°C for 15 min in DMEM supplemented with collagenase A (6.25 mg ml^{-1}, Roche), dispase II (6.25 mg ml^{-1}, Roche), DNase (62.5 µg ml^{-1}; Roche), and elastase (1.717 U ml^{-1}, Sigma), and then at 37°C for 5 min in 0.25% trypsin-EDTA (Gibco). Cell suspensions were pelleted and resuspended in sorting buffer (PBS containing 1% FBS, 2 mM EDTA, 5 mM glucose, and 10 mM HEPES). Hoechst 33342 (10 µg ml^{-1}, B2261, Sigma,) was included in digestion and sorting buffers to label cell nuclei. Cells were filtered through a 70-µm cell strainer and stained with TO-PRO-3 (1 µM; Invitrogen), and viable nucleated cells (TO-PRO-3^{-} Hoechst 33342^{+}) were isolated by cell sorting (FACSAria Cell Sorter, BD Biosciences) and collected in sorting buffer.

Sequencing. Sorted cells were pelleted and resuspended in sorting buffer at approximately 1,400 cells µl^{-1}. Cells were counted, and the viability of the cell suspensions was assessed using a Countess II cell counter (Invitrogen). For each condition, 1,200 cells µl^{-1} were loaded onto a Chromium Next GEM Chip G (10x Genomics). Libraries were created using the Next GEM Single cell 3'Library preparation

kit v3.1 (10x Genomics), and the samples were indexed using the Chromium i7 Multiplex kit (10x Genomics). Library quality was checked with an Agilent Bioanalyzer high-sensitivity DNA chip. Libraries were paired-end sequenced using a HiSeq 4000 system sequencer (Illumina) and processed with RTA v1.18.66.3. FastQ files for each sample were obtained using the cellranger demux pipeline (10x Genomics).

Transcriptome quantification. Single-cell transcriptomes were quantified using 10x Genomics Cell Ranger 3.1.0 pipeline (detailed information available at https://support.10xgenomics.com/single-cell-gene-expression/software/pipelines/latest/what-is-cell-ranger) with the mm10 mouse reference genome and ensemble gene build v84 (mm10-2.1.0 CellRanger reference). Briefly, information from read 1 (R1) of a sequenced library fragment was used to identify the captured molecule via unique molecular identifiers (UMIs) and assign it to a cell barcode. Read 2 (R2) was mapped to the genome using the exon/intron aware aligner STAR (58) and assigned to a gene. All R2 reads that mapped to the same gene and had the same barcode and UMI were collapsed into one count. One count (UMI) represents one mRNA molecule captured from that gene in a particular cell (barcode). Cells were then identified above background noise by analyzing the distribution of UMI counts per cell barcode in a two-step algorithm based on the EmptyDrops algorithm (59).

Single cell clustering. The cell transcriptome quantification matrix was analyzed with R (detailed information at www.r-project.org) using the Scater (60) and Seurat (61) packages as main tools and ggplot (62) and ComplexHeatmap (63) packages for visualizations. In all methods, default parameters were used except for those described in the following steps. Cell doublets were identified using the detectDoublets method (https://zenodo.org/record/6349517#.Y7v0WBXMKUk), which clusters cells and trains a doublet classification model with virtual doublets generated by combining cells from different clusters. The model is used to classify cells and detect doublets. Doublets and low-quality cells were removed through a series of filters. We selected for analysis only those cells that fulfilled the following criteria: between 1,000 and 60,000 total counts, >500 genes detected, >0.1 cell fraction (total counts/median), <65% cell complexity (% counts on top 50 genes), and no doublet. Counts of cells passing these filters (34,152 cells) were log-normalized (log (1 + countsx10^4/total counts)). To reduce the computational footprint, all clustering steps were performed over the dimensional space of the main principal components (PCs) in a PCA of the 2,000 most variable genes in the experiment, which were selected using the variant stabilization transformation method (vst). This method standardizes variability across different levels of expression, so that variability from low expressed genes contributes in the same way as that of highly expressed genes. The PCA was performed over a scaled matrix of the 2,000 variable genes in the 34,152 cells. The first 10 PCs were selected according to their level of variability by the Elbow plot method. Cells were clustered using the Louvain algorithm at different resolutions to detect communities on a Nearest Neighbour graph calculated in the 10-PC dimensional space. The clustering obtained at resolution 0.4 was selected for subsequent steps after a manual inspection of clustering at different resolutions and gene expression distribution across these clusters. For a 2D representation of this clustering, a UMAP dimensionality reduction (https://arxiv.org/abs/1802.03426) was performed in the same 10-PC dimensional space. Markers for each cluster were obtained by statistically comparing the mean expression of genes in a particular cluster against the mean for the other clusters using the MAST method (64), which is a parametric model based on a zero inflated negative binomial distribution. The same

MAST model was used to evaluate differences between conditions in each cluster or between particular clusters.

Cell type subclustering. To obtain a better definition of cell types, each cell type category (fibroblasts and VSMCs, immune cells, and ECs) was clustered individually using the same protocol described above, with the following parameters:

Fibroblasts and VSMCs: selection of the 500 most variable genes, 10 PCs from the PCA, and a resolution of 0.3 for cluster identification.

Immune cells: selection of the 500 most variable genes, 20 PCs from the PCA, and a resolution of 0.4 for cluster identification.

ECs: selection of the 1000 most variable genes, 20 PCs from the PCA, and a resolution of 0.3 for cluster identification.

Bioinformatics analysis. Enrichment analysis of Gene Ontology (GO) biological processes (Benjamini-Hochberg adjusted P-value <0.05) was carried out with DAVID software (65,66). Ingenuity Pathway Analysis (IPA) software (Qiagen) was used to predict potential activation or inhibition of canonical pathways and upstream regulators (Benjamini-Hochberg adjusted P-value<0.05). To identify potential hierarchical relationships between clusters, we performed trajectory analyses using the Slingshot R package (67) with default parametrization. Venn diagrams were built using the Bioinformatics & Evolutionary Genomics (VIB/UGent) tool (http://bioinformatics.psb.ugent.be/webtools/Venn/). The CellPhoneDB interaction analysis pipeline (68) was used to predict ligand-receptor interactions among different cell types. Briefly, for each interaction pair in CellPhoneDB, a mean expression of its partners (ligand and receptor) was calculated in each cluster-cluster pair. Ligand-receptor pairs that contained a partner expressed in <10% of the cells in any cluster of a given pair were not processed further. To assess the significance of the remaining interactions, CellPhoneDB uses a permutation test by randomly shuffling cell cluster annotations 1,000 times to generate a null distribution of means. A p-value is calculated based on the proportion of means from the null distribution that are equal or higher than the mean of the tested interacting pair. Gene set scores to identify proliferating cells and lymphatic, capillary and arterial ECs (Supplemental Table 18) were generated using the AddModuleScore function from Seurat R package. Gene set scores for lymphatic, capillary, and arterial ECs were created based on published scRNA-seq data from the mouse EC atlas (69) by selecting genes specifically enriched in each EC type in ≥5 tissues. Gene Set Enrichment Analysis (GSEA) (70) was used to analyze the expression of genes related to collagen-containing extracellular matrix (GO:0062023) in aortic ECs.

Hematology and biochemical analysis. Blood was collected from mice by cardiac puncture. For hematological tests, samples were collected in Microvette 100 EDTA tubes (Sarstedt) and analyzed with a PENTRA 80 hematology analyzer (Horiba). For serum cholesterol analysis, mice were fasted overnight and blood was collected in plastic tubes, incubated at room temperature (RT) to allow clotting, and centrifuged at 1,900×g and 4°C for 10 min. Serum was centrifuged at 4°C for 10 additional min at maximum speed, harvested, and stored at −80 °C until analysis. Hemolysed sera were excluded from testing. When the sample volume was insufficient for the analysis, sera from 2 mice of the same genotype and sex were pooled.

Flow cytometry quantification of mouse aortic cell types. Aortas were enzymatically digested as in the scRNA-seq experiments, using previously described protocols with minor modifications (11). Briefly, mice were euthanized and carefully perfused through the left ventricle with 10 ml cold PBS. Aortas were harvested, cleaned of perivascular fat, and opened longitudinally in a plastic dish placed on ice and containing cold DMEM (Sigma) supplemented with antibiotics (100 U ml^{-1} penicillin, 0.1 mg ml^{-1} streptomycin, Sigma), 0.292 mg ml^{-1} L-glutamine (HyClone), and 0.1 mM sodium pyruvate (SH30239.01, HyClone). The aortas were digested at 37°C for 15 min in DMEM supplemented with collagenase A (6.25 mg ml^{-1}, Roche), dispase II (6.25 mg ml^{-1}, Roche), elastase (1.717 U ml^{-1}, Sigma), and DNase (62.5 µg ml^{-1}; Roche). Cell suspensions were pelleted, resuspended in sorting buffer including a CD16/CD32 antibody to block Fc receptors (1:200, cat. 101302, Biolegend), and filtered through a 70-µm cell strainer. Cells for flow cytometry assays were stained with Sytoxgreen (1 µM, Invitrogen), CD31-Alexa647 (1:200, 102416, Biolegend), CD45-Pacific Blue (1:200, 103126, Biolegend), PDGFRα-PE-Cy7 (CD140a, 1:200, 135911, Biolegend), and PDGFRβ-PE (CD140b, 1:200, 136005, Biolegend). Mouse aortic cells were analyzed using a FACSAria Cell Sorter (BD Biosciences), with gating set to select the following aortic populations from viable cells (Sytox green^{-}): fibroblasts (CD31^{-}CD45^{-}PDGFRα^{+}PDGFRβ^{+}), VSMCs (CD31^{-}CD45^{-}PDGFRα^{-}PDGFRβ^{+}), immune cells (CD31^{-}CD45^{+}PDGFRα^{-}PDGFRβ^{-}), and ECs (CD31^{+}CD45^{-}PDGFRα^{-}PDGFRβ^{-}). To quantify the abundance of these cell types, the aortas of two mice per genotype were pooled, and unstained samples were always included as controls.

Aorta *en face* immunostaining and quantitative image analysis. *En face* immunofluorescence assays of aortic tissue were done as previously described (56). Briefly, mice were anesthetized with ketamine (225 mg kg^{-1}, Ketamidor, Richter Pharma) and xylazine (15 mg kg^{-1}, Nerfasin, Fatro). Anesthetized mice were perfused through the left ventricle for 3 minutes either with PBS (SELP, VCAM1, and ICAM1 staining) or with 10 ml of PBS containing 1 U ml^{-1} heparin (primary cilia staining). Animals were perfused for 5 minutes with 4% paraformaldehyde (PFA) in PBS. Aortas were harvested, cleaned of perivascular fat under a stereomicroscope (SZX3, Olympus), and opened longitudinally following the outer curvature. Aortas were transferred to a plastic dish coated with Sylgard 184 (Sigma) or silicone and pinned flat and lumen-side up with steel minutien pins. Samples were fixed for 15 min at RT with 4% PFA in PBS to detect primary cilia, SELP, VCAM1, ICAM1, AQP1, SCA1, LRP1, and SMA, or overnight at 4ºC for other stainings. Aortas were washed three times with PBS, cut into 4-mm pieces, and the pieces transferred to 2-ml microfuge tubes. For free-floating immunostaining, tissues were incubated for 1 h at RT with gentle agitation in blocking/permeabilization buffer [PBS supplemented with 0.1% Triton X-100, 2% bovine serum albumin (BSA), and 1% normal goat serum (005-000-001, Jackson ImmunoResearch) for primary cilia staining; with 0.3% Triton X-100 and 5% normal donkey serum (017-000-001, Jackson ImmunoResearch) for AQP1 and SCA1 staining; or with 0.3% Triton X-100, 5% BSA, and 5% normal goat serum for other stainings]. Primary and secondary antibodies were diluted in blocking/permeabilizing solution for primary cilia staining, PBS supplemented with 0.3% Triton X-100 and 2.5% normal donkey serum for AQP1 and SCA1 staining, or in PBS supplemented with 0.3% Triton X-100, 5% BSA, and 2.5% normal goat serum for other stainings. The primary antibodies used targeted the following antigens: CD31 (1:100, MAB1398Z, Merck),

CD31 (1:50, DIA-310, Dianova), CD31 (1:100, ab28364, Abcam), AQP1(1:500, AB2219, Sigma-Aldrich), SCA1(1:100, AF1226, R&D), LRP1 (1:200, ab92544, Abcam), SELP (1:100, 553742, BD Bioscience), VCAM1 (1:50, 14-1061-85, eBioscience), ERG (1:100, ab196149, Abcam), CD45.2 (1:200, 553771, BD Biosciences), TAZ (1:100, HPA007415, Sigma), the Golgi apparatus marker GLG1 (1:100, ab103439, Abcam), the primary cilium marker ARL13B (71) (1:1000, 17711-1-AP, ProteinTech Labs), ICAM1 (1:100, 14-0541-82, eBioscience) and and lamin A (also detecting progerin) (1:100, sc-20680, Santa Cruz). Because homozygous *Lmna$^{LCS/LCS}$* mice and derivatives do not express lamin A and progerin expression is induced after Cre-mediated recombination (9), the sc-20680 antibody is non-reactive in *Lmna$^{LCS/LCS}$* tissues and detects progerin in ECs from *Lmna$^{LCS/LCS}$Tie2Cre* mice (Figure 6 and Supplemental Figure S15). After overnight incubation with primary antibodies, samples were incubated for 2 h at RT with gentle agitation with the appropriate fluorochromes or fluorochrome-conjugated secondary antibodies or streptavidin, including Hoechst 33342 (10 µM, B2261, Sigma), Cy3-labeled mouse anti-SMA (1:200, C6198, Sigma), goat anti-Armenian hamster IgG H&L Alexa Fluor488 (1:400, ab173003, Abcam), goat anti-rabbit IgG H&L Alexa Fluor 488 (1:400, A11034, Thermo Fisher Scientific),goat anti-rabbit IgG H&L Alexa Fluor 647 (1:400, A21245, Thermo Fisher Scientific), goat anti-rat IgG H&L Alexa Fluor 488 (1:400, A11006, Thermo Fisher Scientific), goat anti-rat IgG H&L Alexa Fluor 568 (1:400, A11077, Thermo Fisher Scientific), goat anti-rat IgG H&L Alexa Fluor 647 (1:400, 112-605-167, Jackson ImmunoResearch), donkey anti-rabbit IgG H&L Alexa Fluor 568 (1:400, A10042, Thermo Fisher Scientific), donkey anti-goat IgG H&L Alexa Fluor 633 (1:400, A21082, Thermo Fisher Scientific), and/or streptavidin Cy3 (1:200, 016-160-084, Jackson ImmunoResearch). After incubation, samples were mounted luminal side up in glycerol under a stereomicroscope (SZX3, Olympus). Images were acquired with an LSM 700 Carl Zeiss confocal microscope equipped either with a LD LCI Plan-Apochromat 25x/0.8 Imm Korr DIC M27 objective or with a LCI Plan-Neofluar 63x/1,3 Imm Korr DIC M27 objective (Zeiss), or using a Leica gated STED-3X- WLL SP8 confocal microscope equipped with a HC PL Apo CS2 93x/1.3 GLY (Leica) glycerol objective. Three-dimensional confocal images were reconstructed using Imaris Cell Imaging Software 9.1.2 (Oxford Instruments). Image quantification was done using ImageJ Fiji software (72). AQP1 and LRP1 enrichment was quantified as mean intensity fluorescence from a sum projection. SMA enrichment was quantified as mean intensity fluorescence from a sum projection in LRP1-negative areas. SCA1^{high}AQP1^{-} ECs were assessed by counting the number of ECs with positive staining for SCA and negative for AQP1, relative to total number of ECs. To quantify LRP1 fluorescence intensity, areas with immune cells expressing high levels of LRP1 were excluded from the analysis. In all quantifications of AQP1^{+}, LRP1^{+}, SMA^{high}, and/or SCA1^{high} ECs, areas within bifurcations were excluded from the analysis. SELP^{+}, VCAM1^{+} and ICAM1^{+} area percentage was quantified on maximal intensity projection. Leukocytes were quantified in randomly selected regions as the number of nucleated CD45.2^{+} cells that were negative for the EC marker ERG. Nuclear TAZ fluorescence intensity (integrated density) was quantified from a sum projection based on EC nuclear staining with Hoechst 33342. Values of nuclear TAZ in all cells from all animals were plotted, and the enrichment of cells in each quartile was determined for each animal. Progerin expression in aortic ECs was assessed by counting the number of ECs with positive staining for progerin in the nuclei relative to total number of ECs. EC polarization was quantified by drawing vector lines with the arrow tool from the center of mass of the EC nucleus to the center of mass of the GA (stained with GLG1) in the same cell. The angle was measured between this vector and the blood flow direction (defined as 0), and the

percentage of ECs containing a GA positioned 0º (±90º) downstream of the nucleus was calculated. The percentage of ciliated cells was calculated from the ratio of ECs positive for ARL13B to total ECs. All analyses were performed by a researcher blinded to genotype in 1-3 pieces of aorta per animal, imaging three 25x fields in each piece. Folded or damaged areas were not analyzed. Mean values were used for statistical analysis.

Immunostaining and quantitative image analysis of aortic sections. Mice were anesthetized and perfused through the left ventricle with 4% PFA in PBS for 5 min, and aortic arch or thoracic aortas were harvested, cleaned of fatty tissue, and fixed overnight. Tissue was then incubated in 30% sucrose in PBS overnight at 4°C and included in Tissue-Tek OCT compound (Sakura), or dehydrated to xylene and embedded in paraffin. Aortic tissue was cut in 8-µm sections (OCT samples) or 4-5 µm sections (paraffin samples). Paraffin cross-sections were deparaffinized, rehydrated, and washed in PBS. Antigen retrieval was performed by boiling the sections for 20 minutes in 10 mM sodium citrate buffer (pH 6). Sections, were blocked and permeabilized for 1 h at RT in PBS supplemented with 0.3% Triton X-100, 5% BSA, and 5% normal goat serum. Primary and secondary antibodies were diluted in PBS supplemented with 0.3% Triton X-100, 5% BSA, and 2.5% normal goat serum. The primary antibodies used targeted CD31 (1:100, MAB1398Z, Merck; or 1:50, DIA-310, Dianova), FN1 (1:500, AB2033, Sigma), CTGF (1:200, ab6992, Abcam), and lamin A (which also detects progerin) (1:100, sc-20680, Santa Cruz). After overnight incubation at 4ºC with primary antibodies, samples were incubated for 2 h at RT with appropriate fluorochromes and/or fluorochrome-conjugated secondary antibodies: Hoechst 33342 (10 µM, B2261, Sigma), goat anti-Armenian hamster IgG H&L Alexa Fluor488 (1:400, ab173003, Abcam), and/or goat anti-rabbit IgG H&L Alexa Fluor 647 (1:400, A21245, Thermo Fisher Scientific) together with anti–smooth muscle α-actin-Cy3 (SMA-Cy3, 1:200, C6198, Sigma). Samples were mounted in Fluoromount-G imaging medium (eBioscience). For each animal, sections from 2-4 aortic regions were analyzed, and mean values were used for statistical analysis. Confocal images were analyzed by an observer blinded to genotype using customized ImageJ Fiji macros. Protein expression was quantified on maximal intensity projections as the percentage area positive for the staining in the first 10 µm of the aorta starting from the lumen (luminal) and in the rest of the medial layer (non-luminal).

In vitro studies with human aortic ECs (HAECs)*.* TeloHAECs (ATCC CRL-4052) were cultured in Endothelial Cell Growth Basal Medium-2 (EBM-2, 00190860, Lonza) supplemented with the EGM-2 SingleQuots pack (CC-4176, Lonza). For immunostaining analysis, cells were plated in 1% gelatin-coated glass coverslips in 24-well plates at high (120,000 cells/well) or low density (20,000 cells/well), cultured overnight, and treated for 24 h with 2% DMSO or 1 µM verteporfin (SML0534, Sigma). Verteporfin was used immediately after resuspension and cultures were protected from light at all times. Cells were washed with PBS, fixed for 10 min with 4% PFA, and washed three times with PBS. Cells were then blocked and permeabilized for 30 min at RT in PBS supplemented with 0.3% Triton X-100, 5% BSA, and 5% normal goat serum. Cells were stained overnight at 4ºC with a primary antibody targeting TAZ (1:100, HPA007415, Sigma) diluted in PBS supplemented with 5% BSA and 2.5% normal goat serum, and incubated for 1 h at RT with Hoechst 33342 (10 µM, B2261, Sigma), goat anti-rabbit IgG H&L Alexa Fluor 647 (1:400, A21245, Thermo Fisher Scientific), and phalloidin Alexa Fluor 488 (1:300, A12379, Thermo Fisher Scientific) diluted in PBS

supplemented with 5% BSA and 2.5% normal goat serum, and mounted in Fluoromount-G imaging medium (eBioscience). Nuclear TAZ fluorescence intensity (integrated density) was quantified from a sum projection based on nuclear staining with Hoechst 33342. For gene expression analysis, cells were plated in 6-well plates coated with 0.5% gelatin at low density (100,000 cells/well), cultured overnight, and incubated for 24 h in media containing 2% DMSO, 2 µM Ro106-9920 (557550, Merck), or 1 µM verteporfin (SML0534, Sigma). The last 6 h cells were stimulated with 0.1% BSA or 5 ng ml^{-1} TNFα (#210-TA-005, R&D), followed by RNA isolation and RT-qPCR analysis (see below). Verteporfin, Ro106-9920, and TNFα concentrations were selected based on efficacy and cell viability determined by pilot experiments.

Isolation of mouse aortic ECs by cell sorting. Aortas were digested as described for scRNA-seq assays but omitting elastase from the digestion buffer. Each sample contained pooled aortas from 5 animals per genotype. Cell suspensions were pelleted, resuspended in sorting buffer including anti- CD16/CD32 antibody to block Fc receptors (1:200, cat. 101302, Biolegend), and filtered through a 70-µm cell strainer. Cell suspensions were stained with propidium iodide (1 µM, Sigma), anti-CD31-Alexa647 (1:200, 102416, Biolegend), and anti-CD45-Pacific Blue (1:200, 103126, Biolegend). Viable ECs (propidium iodide^{-}CD45^{-}CD31^{+}) for RNA extraction were sorted directly into TRI Reagent Solution (AM9738, Invitrogen) using a FACSAria Cell Sorter (BD Biosciences).

mRNA isolation, reverse transcription, and real-time qPCR. Aortic arch tissue was homogenized in TRI Reagent Solution (AM9738, Invitrogen) using a TissueLyser (Qiagen). For aortic arch samples and cultured HAECs, total RNA was extracted using the RNeasy Mini Kit (74106, Qiagen) following the manufacturer's instructions. For mouse aortic ECs, cells were sorted and directly lysed upon collection in 750 µl TRI Reagent Solution (AM9738, Invitrogen). RNA was extracted following the manufacturer's instructions until phase separation, when the aqueous phases were diluted 1:1 with 70% ethanol and loaded into RNeasy Mini Kit columns (74106, Qiagen) to continue with the RNeasy Mini Kit protocol. cDNA was prepared with the High-Capacity cDNA Reverse Transcription Kit (Applied Biosystems). Real-time qPCR was carried out in a Bio Rad-CFX384 Real-Time PCR System (Bio Rad) using *Power*SYBR Green PCR Master Mix (Thermo Fisher Scientific) and the following thermocycling protocol: 2 min at 50°C, 10 min at 95ºC, and 40 cycles of 15 s at 95°C and 1 min at 60°C. The following primers were used for mouse samples: *Ccn1* Forward (F): 5′-ACCGCTCTGAAAGGGATCTG-3′, *Ccn1* Reverse (R): 5′-CGGCGCCATCAATACATGTG-3′, *Ccn2* F: 5′-AGGGCCTCTTCTGCGATTTC-3′, *Ccn2* R: 5′-CATCCAGGCAAGTGCATTGG-3′, *Tagln* F: 5′-CTTTGGGCAGTTTGGCTGTG-3′, *Tagln* R: 5′-CCTCTGTTGCTGCCCATTTG-3′, *Selp* F: 5′-GGCAAGTGGAATGATGAACC-3′, *Selp* R: 5′-CCAATGGTCTCGATGCACTC-3′, *Vcam1* F: 5′-CCCGTCATTGAGGATATTGG-3′, *Vcam1* R: 5′-ACAGCACCACCCTCTTGAAG-3′, *Icam1* F: 5′- GACGCAGAGGACCTTAACAG-3′, *Icam1* R: 5′-GTGGGCTTCACACTTCACAG-3′, *Gapdh* F: 5′-AAATGGTGAAGGTCGGTGTG-3′, *Gapdh* R: 5′-GAGTGGAGTCATACTGGAAC-3′, *Sdha* F: 5′-GGAGCTGCAGAATCTGATGC-3′, and *Sdha* R: 5′-ATATGAGAGGGTGTGCTTCC-3′.
The following primers were used for human cell samples: *CCN1* Forward (F): 5′-AAGGAGCTGGGATTCGATGC-3′, *CCN1* Reverse (R): 5′-GCACTCAGGGTTGTCATTGG-3′, *CCN2* F: 5′-TTCCAAGACCTGTGGGATGG -3′,

CCN2 R: 5′-AGCTCGGTATGTCTTCATGC-3′, *SELP* F: 5′-GCTGGAATGCTTGGCTTCTG-3′, *SELP* R: 5′-ACTTCCGGTCCAACTAATGC-3′, *VCAM1* F: 5′-GGAAGTCCCTGGAAACCAAG-3′, *VCAM1* R: 5′-TCACAGAGCCACCTTCTTGC-3′, *ICAM1* F: 5′-AAGGGCTGGAGCTGTTTGAG-3′, *ICAM1* R: 5′-GAAGGAGTCGTTGCCATAGG-3′, *GAPDH* F: 5′-CAACGACCACTTTGTCAAGC-3′, *GAPDH* R: 5′-TCTTCCTCTTGTGCTCTTGC-3′, *SDHA* F: 5′-TTGGTGGACAGAGCCTCAAG-3′, and *SDHA* R: 5′-TGCTCTTATGCGATGGATGG-3′.
All values were normalized to internal controls (*Gapdh or GAPDH* and *Sdha* or *SDHA* for mouse and human ECs, respectively, and *Gapdh* for mouse aortic arch samples), and relative expression values were calculated using the $2^{-\Delta\Delta Ct}$ method. All reactions were performed in duplicate for mouse ECs and in triplicate for mouse aortic arches and HAECs.

Western blot. Aortas were homogenized with a TissueLyser (Qiagen) and a pellet mixer (VWR) in RIPA buffer (1% NP-40, 0.5% sodium deoxycholate, 0.1% SDS in PBS) supplemented with protease and phosphatase inhibitors (Roche) (incubation on ice for 1 h with frequent vortexing). Insoluble material was removed by centrifugation at 10,000xg for 10 min at 4ºC, and protein concentration was determined using the BCA assay (Thermo). Lysates (9 µg protein) were mixed with loading buffer including 11 mg ml^{-1} 2-mercaptoethanol, incubated at 95ºC for 5 minutes, and resolved on 7% polyacrylamide gels. Proteins were then transferred to a PVDF membrane using standard methods for wet transfer. After blocking for 1 h at RT with 5% BSA or 5% milk in TBST, membranes were incubated overnight with primary antibodies at 4ºC, followed by several washes in TBST, incubation for 1 h at RT with secondary antibodies, and further washes. Primary antibodies targeted WWTR1 (TAZ) (1:1000, HPA007415, Sigma), phospho-WWTR1 (Ser89) (1:1000, 59971, Cell Signalling), and vinculin (1:1000, V4505, Sigma). Antigen–primary antibody complexes were detected with secondary antibodies (mouse anti-rabbit IgG-HRP [1:3000, sc-2357, Santa Cruz] and m-IgGκ BP-HRP [1:5000, sc-516102, Santa Cruz]) using immobilon forte western HRP substrate (Millipore) and an ImageQuant LAS 4000 mini device (GE Healthcare Lifesciences). When required, bound antibodies were stripped from PVDF membranes by incubation in stripping buffer (0.05 M Tris-HCL buffer pH 6.8, 1.6% SDS, and 6.2 mg ml^{-1} 2-mercaptoetanol in water) at 60ºC for 20 min. Protein size was determined with precision plus protein dual color standards (Bio-Rad). Images were acquired and analyzed with Image Studio Lite (Li-Cor), and protein levels were normalized using vinculin as an internal control.

Atomic force microscopy on decellularized aortas. Thoracic aortas were decellularized according to published protocols for *in vitro* applications (57). Mice were euthanized and perfused transcardially with 10 ml PBS, and aortas were harvested, cleaned of perivascular fat, and opened longitudinally along the outer curvature. Samples were incubated at 37ºC for 5 min in prewarmed decellularization buffer (PBS containing 0.5% (v/v) Triton X-100, and 20 mM NH_4OH). Samples were then washed with PBS, cut transversally into 3 pieces, and attached firmly to a plastic dish using surgical glue, with the luminal side facing up (73). After the glue had air-dried, samples were gently covered with PBS. To monitor the decellularization process, decellularized aortas were washed and fixed with 4% PFA in PBS overnight at 4ºC and stained *en face* as indicated previously. Atomic force microscopy experiments were performed with a Nanowizard III system (JPK Instruments) mounted on an inverted optical microscope

(AXIO Observer D1; Carl Zeiss). All experiments were carried out with samples immersed in PBS. To analyze decellularized thoracic aortas, PT.PS cantilevers (Novascan) with a nominal spring constant of 0.12 N/m and a spherical tip shape (R=5 µm) were used. The actual spring constant of the cantilever was determined by using the thermal noise method as implemented in the atomic force microscope software. Force-distance curves (FDC) were acquired to determine the Young's modulus of the subendothelial ECM of the thoracic aorta. The tip–sample distance was modulated by applying a triangular waveform characterized by a tip velocity of 5 µm/s. The maximum force exerted on these samples during a single FDC was 8 nN. The thoracic aorta samples were divided into 27 regions across 6 zones: 3 along the transverse axis (ventral, lateral, and dorsal), and 3 along the longitudinal axis (from the region closest to the aortic arch to the area closest to the diaphragm). A sequence of 125 FDCs was acquired in each of these regions (the 125 FDCs were distributed in an area of 100 x 100 μm^2), accomplishing a complete series of 3,375 FDCs for each thoracic aorta. To compare different samples, we used 27 values per aorta, each value being the mean of the Young's modulus values measured in each spatial region (Figure 5A). In *Lmna*$^{LCS/LCS}$*Tie2Cre* mice and *Lmna*$^{LCS/LCS}$ controls, 9 regions in the middle segment of the thoracic aorta were analyzed. To determine the contact point, we used a ratio of variances protocol (74). The Young's modulus of the thoracic aortas was obtained by fitting a section of the force-distance curve (the approach semi cycle of the whole FDC) with the semi-infinite Hertz model for a spherical tip (75) .

Ultrasound. Transthoracic ultrasound assessment was performed by an expert operator blinded to genotype using a high-frequency ultrasound system (Vevo 2100, Visualsonics Inc.) equipped with a 30-MHz linear MS400 probe. Two-dimensional and M-mode echography were performed at a frame rate > 230 frames/sec. Mice were lightly anesthetized with 0.5-2% isoflurane in oxygen, with the isoflurane delivery adjusted to maintain the heart rate at 450±50 bpm. Mice were placed in the supine position on a heating platform, and warmed ultrasound gel was used to maintain normothermia. A base apex electrocardiogram was continuously monitored. A pulse wave (PW) and color Doppler probe was placed over the aortic valve to assess aortic valve regurgitation, and over the ascending, descending, and abdominal aorta to assess aortic insufficiency and to measure mean flow velocities. PW Doppler in the abdominal aorta obtained the peak systolic (PSV) and end-diastolic (EDV) velocities and the velocity time integral (VTI). Images were analyzed off-line with Vevo 2100 Workstation software. Aortic insufficiency in the descending aorta was defined as the presence of retrograde flow during diastole in three consecutive heart beats. Mean flow velocity was calculated as the area under the curve in systole in the doppler mode graph. Aortic valve regurgitation was evaluated in the aortic valve region by color and PW Doppler. The pulsatility index (PI) and resistivity index (RI) were calculated as follows:

PI = (PSV - EDV)/mean velocity
RI = (PSV - EDV)/PSV.

Histology and quantification of atherosclerosis burden. Mouse thoracic aorta and aortic root samples (the latter including myocardial tissue) were fixed with 4% PFA in PBS, dehydrated to xylene, and embedded in paraffin. Tissues were cut in 4-5 µm sections with a microtome (Leica) and stained with Masson's trichrome or hematoxylin-eosin as indicated. Images were captured using an OPT Scanner 3001 (Bioptonics Microscopy) or an Axio Scan Z1 (ZEISS) and exported with ZEN 3.4 blue edition

(ZEISS). Collagen was quantified as the percentage of green area in Masson's thrichrome staining in the first 10 μm of the aorta starting from the lumen (luminal) and in the rest of the media (non-luminal). For each mouse, 1-2 sections were analyzed, and mean values were used for statistical analysis.

A trained researcher blinded to treatment conditions quantified atherosclerotic plaque area and the perimeter of the area affected by atherosclerosis in the aortic valves by manually drawing the area/perimeter on the images using the freehand selection and freehand line tools in ImageJ Fiji. For each mouse, 2-3 sections were analyzed, and mean values were used for statistical analysis. Oil red O (ORO) staining was done as previously described (76). Mouse aortas from fat-fed *Apoe*$^{-/-}$*Lmna*$^{G609G/G609G}$ mice were fixed with 4% PFA in PBS for at least 48 h, cleaned of fat, and stained with 0.2% ORO (O0625, Sigma). Thoracic aortas were opened longitudinally from the outer curvature and pinned flat with the luminal side up on a cork board with a dark background. Images were acquired with a digital camera (UC30, OLYMPUS) mounted on a stereomicroscope (SZX3, Olympus). A trained researcher blinded to treatment conditions quantified atherosclerotic area as the percentage ORO-stained aortic surface using SigmaScan Pro 5 (Systat Software Inc.). An expert pathologist blinded to treatment conditions evaluated 4-5 non-consecutive sections per mice starting from the aortic root, and scored atherosclerosis-related alterations (plaque build-up, medial lipid accumulation, and intimal thickening) in myocardial coronary arterioles and at the origin of coronary arteries in the aortic root. For myocardial arterioles, mice presenting ≥1 myocardial vessel >50-μm diameter affected by atherosclerosis were considered positive. Vessel diameter was determined by measuring the largest distance between outer edges of the media. For atheroma plaque evaluation at the origin of coronary arteries, mice presenting plaque in ≥1 section were considered positive.

SUPPLEMENTAL RESULTS

Fibroblast heterogeneity in wild-type and progeroid mouse aorta. We and others have shown important pathological alterations in the adventitial and medial layers of arteries in HGPS patients and progerin-expressing animal models (4). Although fibroblasts and VSMCs are key components of the adventitial and medial layers, respectively, little is known about the heterogeneity of these cell types and the contribution of particular fibroblast and VSMC subtypes to the vascular phenotype in progeria. Using our scRNA-seq data, we performed unsupervised reclustering of fibroblasts and VSMCs. This analysis identified 10 subclusters (FV0-FV9) that were present in differing proportions in *Lmna*$^{+/+}$ and *Lmna*$^{G609G/G609G}$ mice (Supplemental Figure S3, Supplemental Table 2), with no overt differences between biological replicates (Supplemental Figure S1C). We found fibroblast (FV0, FV2, FV5, FV6, and FV8) and VSMC (FV1, FV3, FV4, FV7) subclusters and a small cluster (FV9) enriched in chondrocyte-associated transcripts (*Acan, Sox9, Chad,* and *Col2a1*) which was more abundant in HGPS aortas (Supplemental Figure S3, B and C).

All fibroblast subpopulations expressed *Pdgfra* and *Col1a1* (Supplemental Figure S3C), and FV6 exhibited myofibroblast features (increased expression of the contraction-

related genes *Acta2, Tagln,* and *Myh11* and reduced expression of *Pdgfra*) (Supplemental Figure S4A, Supplemental Table 3). Gene ontology (GO) analysis of differentially expressed genes in fibroblast subclusters suggested functional specialization of FV0, FV2, FV5, and FV8 (Supplemental Figure S4B, Supplemental Table 4, 5). FV8 had a specific gene expression profile related to immune-system response processes and was highly abundant in HGPS aortas (Supplemental Figure S3B, 4B**)**. Shared and subcluster-specific alterations induced by progerin expression in fibroblasts are summarized in Supplemental Figure S4C and Supplemental Table 6. All HGPS aortic fibroblasts had elevated expression of the senescence marker C*dkn1a* (Supplemental Figure S4D), and some subclusters were enriched in genes related to fibrosis (FV0 and FV6), ECM organization (FV2 and FV5), and hypoxia (FV5) (Supplemental Figure S4, D and E, Supplemental Table 7). These findings suggest that distinct fibroblast subtypes play key roles in the pathological remodeling of the adventitial layer in the HGPS mouse aorta.

Dysfunctional VSMCs in the progeroid mouse aorta. Progerin gain-of-function and loss-of-function experiments have revealed a major role of VSMCs in vascular pathology and premature death in HGPS mouse models (10,17,49,51). Using the same approach as with fibroblasts, we found 4 VSMC subclusters (FV1, FV3, FV4, and FV7) (Supplemental Figure S3, Supplemental Table 2). FV3 and FV7 expressed high levels of contraction-related genes (*Acta2*, *Tagln,* and *Cnn1*) (Supplemental Figure S3C), strongly suggesting that these cells are highly contractile VSMCs. Whereas aortic FV1 and FV3 were present at similar proportions in both genotypes, highly contractile FV7 was more abundant in wild-type aorta, and FV4 was detected only in progeroid aorta (Supplemental Figure S3, A and B). Pseudotime trajectory analysis suggested that highly contractile FV3 was the most likely source of FV4 (Supplemental Figure S5A). We next identified gene expression changes in dysfunctional VSMCs FV4 relative to the rest of VSMCs and performed a GO analysis (Supplemental Table 8, 9). FV4 cells showed lower expression of contraction-related genes and genes associated with the cellular response to calcium, a primary trigger of muscle contraction, as well as a prominent downregulation of genes related to the ECM and elastic fiber organization (Supplemental Figure S5, B and C). In contrast, FV4 cells showed upregulation of genes related to endoplasmic reticulum (ER) stress, unfolded protein response, proteasomal catabolic processes, and apoptosis (Supplemental Figure S5D). GO analysis of genes differentially expressed in *Lmna*$^{+/+}$ and *Lmna*$^{G609G/G609G}$ mouse aorta revealed common and subcluster-specific alterations in VSMCs (Supplemental Figure S5E, Supplemental Table 6). VSMC subclusters FV1, FV3, and FV7 from *Lmna*$^{G609G/G609G}$ mouse aorta showed altered expression of genes associated with ECM organization, supramolecular fiber organization, wound healing, TGF-β receptor signaling, and protein folding (Supplemental Figure S5F, Supplemental Table 10). *Lmna*$^{G609G/G609G}$ FV1 was specifically enriched in genes related to ER-to-Golgi transport and the response to ER stress, again supporting the presence of ER stress in some VSMC populations in progeroid aortas (Supplemental Figure S5G, Supplemental Table 10). *Lmna*$^{G609G/G609G}$ FV1 and FV3 showed downregulation of genes associated with the cellular response to calcium (*Mylk, Asph*) (Supplemental Table 6). Collectively, these data strongly suggest that progerin expression induces a switch in aortic VSMCs from a fully contractile phenotype to a dedifferentiated and dysfunctional phenotype characterized by ECM remodeling, ER stress activation, and eventually apoptotic cell death. These phenotypic alterations in VSMCs may be major drivers of vascular dysfunctional impairment in *Lmna*$^{G609G/G609G}$ mice.

Increased immune cell content in the progeroid mouse aorta. Unsupervised subclustering of 6,240 *Ptprc* (CD45)-expressing leukocytes generated 15 immune cell subclusters (IC0-IC14) (Supplemental Figure S6, Supplemental Table 11) that were present in all biological replicates from both genotypes (Supplemental Figure S1D). Analysis of the expression of known markers identified macrophages, T-cells, natural killer T cells, B-cells, innate lymphoid cells, granulocytes, and type I conventional dendritic cells (Supplemental Figure S6C). Most aortic immune cell types, particularly T-cells, dendritic cells, and several macrophage subclusters, were more abundant in progeroid *Lmna*$^{G609G/G609G}$ mice than in controls (Figure 6B), indicating a progerin-induced immune response.

Because macrophages were the most prevalent leukocyte population in the aorta, we performed additional analyses of this immune cell type. We found highly diverse macrophage subtypes (Supplemental Figure S6, A and C), most of them being more abundant in HGPS aorta (Supplemental Figure S6B). IC2 macrophages expressed high levels of *Ccr2* (Supplemental Figure S7A, Supplemental Table 11), a marker previously associated with macrophages derived from recruited monocytes (77). This subcluster also expressed higher levels of genes associated with chemotaxis and cytokine-mediated signaling, including *Il1b*, *Lgals3, Ccl9,* and *Ccl6* (Supplemental Table 11) and was more abundant in *Lmna*$^{G609G/G609G}$ aortas than in controls (Supplemental Figure S6B), suggesting increased recruitment of monocytes from the bloodstream to the vessel wall of progeroid mice. Pseudotime trajectory analysis suggested that recruited IC2 macrophages were the source of other aortic macrophage subtypes (Supplemental Figure S7B). Additional macrophage subclusters more abundant in HGPS aorta included interferon-stimulated macrophages (IC6), proliferating macrophages (IC11), and macrophages expressing *Mmp12* and *Mmp13* (IC10), the latter recently identified as aortic intimal resident macrophages (Mac^{AIR}) (78,79) (Supplemental Figure S6B, Supplemental Figure S7A, Supplemental Table 11). IC3 and IC8 macrophages showed lower expression of canonical macrophage markers than other macrophage subtypes, together with higher expression than other immune cells of the VSMC genes *Acta2, Tagln,* and *Myh11* (Supplemental Figure S6C, Supplemental Figure S7A, Supplemental Table 11).

We categorized IC0 and IC4 subclusters as adventitial resident macrophages based on the expression of *Lyve1, F13a1, Mrc1,* and *Gas6* (80) (Supplemental Figure S7A). Although both IC0 and IC4 were present in similar proportions in HGPS and control aorta (Supplemental Figure S6B), their transcriptomes showed significant between-genotype differences (Supplemental Table 12). For example, chemotaxis-related and pro-inflammatory genes were prominently upregulated in IC0 and IC4 from progeroid aorta compared with control aorta (Supplemental Figure S7C, Supplemental Table 13). Some of these genes showed increased expression in both IC0 and IC4 (*Ccl8*, *Ccl12*), whereas others were specifically increased in the individual clusters (IC0 was enriched in *Lgals3, Il1b,* and *Cxcl2*; IC4 was enriched in *Ccl7* and *Ccl2*) (Supplemental Table 12).

Together, these results demonstrate a significantly altered immune-cell landscape in the *Lmna*$^{G609G/G609G}$ mouse aorta, including profound changes in the abundance, proliferation, and activation state of several macrophage subtypes that may contribute to the generation of a pro-inflammatory vascular environment.

SUPPLEMENTAL DISCUSSION

The distinct fibroblast subpopulations identified in our analysis presumably reside in the adventitial layer. Previous studies have highlighted the involvement of the adventitial layer in the response to arterial injury in non-HGPS models of vascular disease, reflecting the role of adventitial fibroblasts as important regulators of pathological fibrotic remodeling (81). Although the adventitia is also severely affected in HGPS animal models and patients (4), no previous study had explored the potential role of fibroblasts in adventitial structural remodeling in HGPS. Our current results identify progerin-dependent changes in both the proportions of fibroblast subtypes and their transcriptomic profile, consistent with cell senescence, fibrosis development, wound repair, the response to hypoxia, and immune-system processes. We also detected previously unknown activation of chemokine signaling in resident adventitial macrophages. These findings indicate that activation of pro-inflammatory gene expression and pro-fibrotic signaling contributes to the pathological remodeling of the adventitia in HGPS, as well as to the maintenance of chronic vascular inflammation, a key process in the initiation, progression, and complications of atherosclerosis and other vascular diseases. Tissue remodeling requires both ECM deposition and degradation through a complex process involving the concerted action of fibroblasts and macrophages, which likely engage in functional interactions that alter adventitial structure and composition in HGPS. Although future studies will be needed to validate the regulatory potential of adventitial fibroblasts and macrophages in HGPS, our study identifies the targeting of adventitial components as a promising and as-yet unexplored therapeutic strategy for progeria.

Other important players in HGPS-associated vascular malfunction are medial VSMCs, which are essential for the maintenance of vascular tone and compliance in healthy vessels. VSMCs in *Lmna*$^{G609G/G609G}$ mice displayed defects in ECM organization and elastic fiber assembly, together with upregulation of genes related to ER stress and the associated unfolded protein response, in good agreement with previous results from our group and others (10,17,18,49,51). Arterial accumulation of contractility-impaired VSMCs is a well-known feature of atherosclerosis in the general population, where ‘contractile’ VSMCs respond to subendothelial lipid accumulation and plaque build-up by switching towards a ‘synthetic’ phenotype (82). A contractile-to-synthetic VSMC switch is also suggested by our scRNA-seq results from atherosclerosis-free progeroid *Lmna*$^{G609G/G609G}$ mice, in line with previous *in vitro* studies of VSMCs with ectopic progerin expression (83) and with a recent scRNA-seq study that entirely focused on describing transcriptomic changes in aortic VSMCs from the same HGPS mouse model (84). This phenotypic transition could explain the decreased arterial contractility previously reported in the same HGPS mouse model (50,85) and suggests that progerin-induced VSMC alterations may be a cause rather than a consequence of accelerated atherogenesis. Indeed, specific elimination of progerin in VSMCs and cardiomyocytes in the atherosclerosis-free progeroid *HGPSrev* mouse model is sufficient to normalize the vascular phenotype and significantly prolong life expectancy (51).

The identification of highly heterogeneous macrophage populations in mouse aorta provides rich information about alterations in HGPS mice, revealing an abundance of recruited, activated, and proliferating macrophages in the progeroid aorta. *Ccr2*$^{+}$ recruited macrophages, which are present in the atherosclerotic aorta (86), were highly

abundant in atherosclerosis−free HGPS aortas, suggesting that arterial wall inflammation in HGPS is exacerbated independently of atherosclerotic plaque build−up, possibly in response to arterial damage, proinflammatory signaling, or EC activation. HGPS aortas also had an elevated proportion of Mac^{AIR} macrophages, which are essential regulators of thrombin activity and clear fibrin deposits from regions of disturbed blood flow such as the aortic arch, where they locate at birth (78). Although Mac^{AIR} usually self-maintain, recruited monocytes can differentiate into Mac^{AIR} in the inflamed arterial wall (79). Trajectory analysis and enriched expression of the recruitment-associated gene *Ccr2* in Mac^{AIR} suggest that this process might be taking place in the progeroid aorta, accounting for the increased proportion of Mac^{AIR} cells. HGPS mouse aorta was also enriched in macrophage-like cells expressing stromal markers. Given the observed tendency of HGPS VSMCs to dedifferentiate, we propose that these macrophage subsets may arise, at least in part, from VSMC transdifferentiation, as reported in the context of atherosclerosis (87,88). Further studies are warranted to confirm VSMC-to-macrophage transdifferentiation in atherosclerosis-free HGPS aortas and to determine its potential contribution to progerin-induced vascular disease.

SUPPLEMENTAL REFERENCES 58-88

Includes all the references that are cited only in this Supplement. References 1-57 can be found in the main text.

58. Dobin A, et al. STAR: ultrafast universal RNA-seq aligner. *Bioinformatics*. 2013;29(1):15-21. doi:10.1093/bioinformatics/bts635
59. Lun ATL, et al. EmptyDrops: distinguishing cells from empty droplets in droplet-based single-cell RNA sequencing data. *Genome Biol*. 2019;20(1):63. doi:10.1186/s13059-019-1662-y
60. McCarthy DJ, et al. Scater: pre-processing, quality control, normalization and visualization of single-cell RNA-seq data in R. Hofacker I, ed. *Bioinformatics*. 2017;33(8):1179-1186. doi:10.1093/bioinformatics/btw777
61. Hao Y, et al. Integrated analysis of multimodal single-cell data. *Cell*. 2021;184(13):3573-3587.e29. doi:10.1016/j.cell.2021.04.048
62. Wickham H. *Ggplot2*. *Springer New York*; 2009. doi:10.1007/978-0-387-98141-3
63. Gu Z, Eils R, Schlesner M. Complex heatmaps reveal patterns and correlations in multidimensional genomic data. *Bioinformatics*. 2016;32(18):2847-2849. doi:10.1093/bioinformatics/btw313
64. Finak G, et al. MAST: a flexible statistical framework for assessing transcriptional changes and characterizing heterogeneity in single-cell RNA sequencing data. *Genome Biol*. 2015;16(1):278. doi:10.1186/s13059-015-0844-5
65. Huang DW, et al. Systematic and integrative analysis of large gene lists using DAVID bioinformatics resources. *Nat Protoc*. 2009;4(1):44-57. doi:10.1038/nprot.2008.211
66. Sherman BT, et al. DAVID: a web server for functional enrichment analysis and functional annotation of gene lists (2021 update). *Nucleic Acids Res*. 2022;50(W1):W216-W221. doi:10.1093/nar/gkac194
67. Street K, et al. Slingshot: Cell lineage and pseudotime inference for single-cell transcriptomics. *BMC Genomics*. 2018;19(1). doi:10.1186/s12864-018-4772-0
68. Efremova M, et al. CellPhoneDB: inferring cell–cell communication from combined expression of multi-subunit ligand–receptor complexes. *Nat Protoc*. 2020;15(4):1484-1506. doi:10.1038/s41596-020-0292-x
69. Kalucka J, et al. Single-Cell Transcriptome Atlas of Murine Endothelial Cells. *Cell*. 2020;180(4):764-779.e20. doi:10.1016/j.cell.2020.01.015
70. Subramanian A, et al. Gene set enrichment analysis: A knowledge-based approach for interpreting genome-wide expression profiles. *Proc Natl Acad Sci*. 2005;102(43):15545-15550. doi:10.1073/pnas.0506580102

71. Larkins CE, et al. Arl13b regulates ciliogenesis and the dynamic localization of Shh signaling proteins. *Mol Biol Cell*. 2011;22(23):4694-4703. doi:10.1091/mbc.E10-12-099

72. Schindelin J, et al. Fiji: an open-source platform for biological-image analysis. *Nat Methods*. 2012;9(7):676-682. doi:10.1038/nmeth.2019

73. Bae YH, et al. Measuring the stiffness of ex vivo mouse aortas using atomic force microscopy. *J Vis Exp*. 2016;(116):54630. doi:10.3791/54630

74. Gavara N. Combined strategies for optimal detection of the contact point in AFM force-indentation curves obtained on thin samples and adherent cells. *Sci Rep*. 2016;6(1):21267. doi:10.1038/srep21267

75. Garcia PD, Garcia R. Determination of the Elastic Moduli of a Single Cell Cultured on a Rigid Support by Force Microscopy. *Biophys J*. 2018;114(12):2923-2932. doi:10.1016/j.bpj.2018.05.012

76. Andrés-Manzano MJ, et al. Oil Red O and hematoxylin and eosin staining for quantification of atherosclerosis burden in mouse aorta and aortic root. *Methods Mol Biol*. 2015;1339:85-99. doi:10.1007/978-1-4939-2929-0_5

77. Boniakowski AE, et al. Murine macrophage chemokine receptor CCR2 plays a crucial role in macrophage recruitment and regulated inflammation in wound healing. *Eur J Immunol*. 2018;48(9):1445-1455. doi:10.1002/eji.201747400

78. Hernandez GE, et al. Aortic intimal resident macrophages are essential for maintenance of the non-thrombogenic intravascular state. *Nat Cardiovasc Res*. 2022;1(1):67-84. doi:10.1038/s44161-021-00006-4

79. Williams JW, et al. Limited proliferation capacity of aortic intima resident macrophages requires monocyte recruitment for atherosclerotic plaque progression. *Nat Immunol*. 2020;21(10):1194-1204. doi:10.1038/s41590-020-0768-4

80. Weinberger T, et al. Ontogeny of arterial macrophages defines their functions in homeostasis and inflammation. *Nat Commun*. 2020;11(1):4549. doi:10.1038/s41467-020-18287-x

81. Tinajero MG, Gotlieb AI. Recent Developments in Vascular Adventitial Pathobiology. *Am J Pathol*. 2020;190(3):520-534. doi:10.1016/j.ajpath.2019.10.021

82. Sorokin V, et al. Role of Vascular Smooth Muscle Cell Plasticity and Interactions in Vessel Wall Inflammation. *Front Immunol*. 2020;11. doi:10.3389/fimmu.2020.599415

83. Coll-Bonfill N, et al. Progerin induces a phenotypic switch in vascular smooth muscle cells and triggers replication stress and an aging-associated secretory signature. *GeroScience*. 2023;45(2):965-982. doi:10.1007/s11357-022-00694-1

84. Murtada SI, et al. Biomechanical and transcriptional evidence that smooth muscle cell death drives an osteochondrogenic phenotype and severe proximal vascular

disease in progeria. *Biomech Model Mechanobiol*. Published online May 7, 2023. doi:10.1007/s10237-023-01722-5

85. von Kleeck R, et al. Decreased vascular smooth muscle contractility in Hutchinson–Gilford Progeria Syndrome linked to defective smooth muscle myosin heavy chain expression. *Sci Rep*. 2021;11(1):10625. doi:10.1038/s41598-021-90119-4

86. Cochain C, et al. Single-Cell RNA-Seq Reveals the Transcriptional Landscape and Heterogeneity of Aortic Macrophages in Murine Atherosclerosis. *Circ Res*. 2018;122(12):1661-1674. doi:10.1161/CIRCRESAHA.117.312509

87. Feil S, et al. Transdifferentiation of Vascular Smooth Muscle Cells to Macrophage-Like Cells During Atherogenesis. *Circ Res*. 2014;115(7):662-667. doi:10.1161/CIRCRESAHA.115.304634

88. Li Y, et al. Smooth muscle-derived macrophage-like cells contribute to multiple cell lineages in the atherosclerotic plaque. *Cell Discov*. 2021;7(1):111. doi:10.1038/s41421-021-00328-4

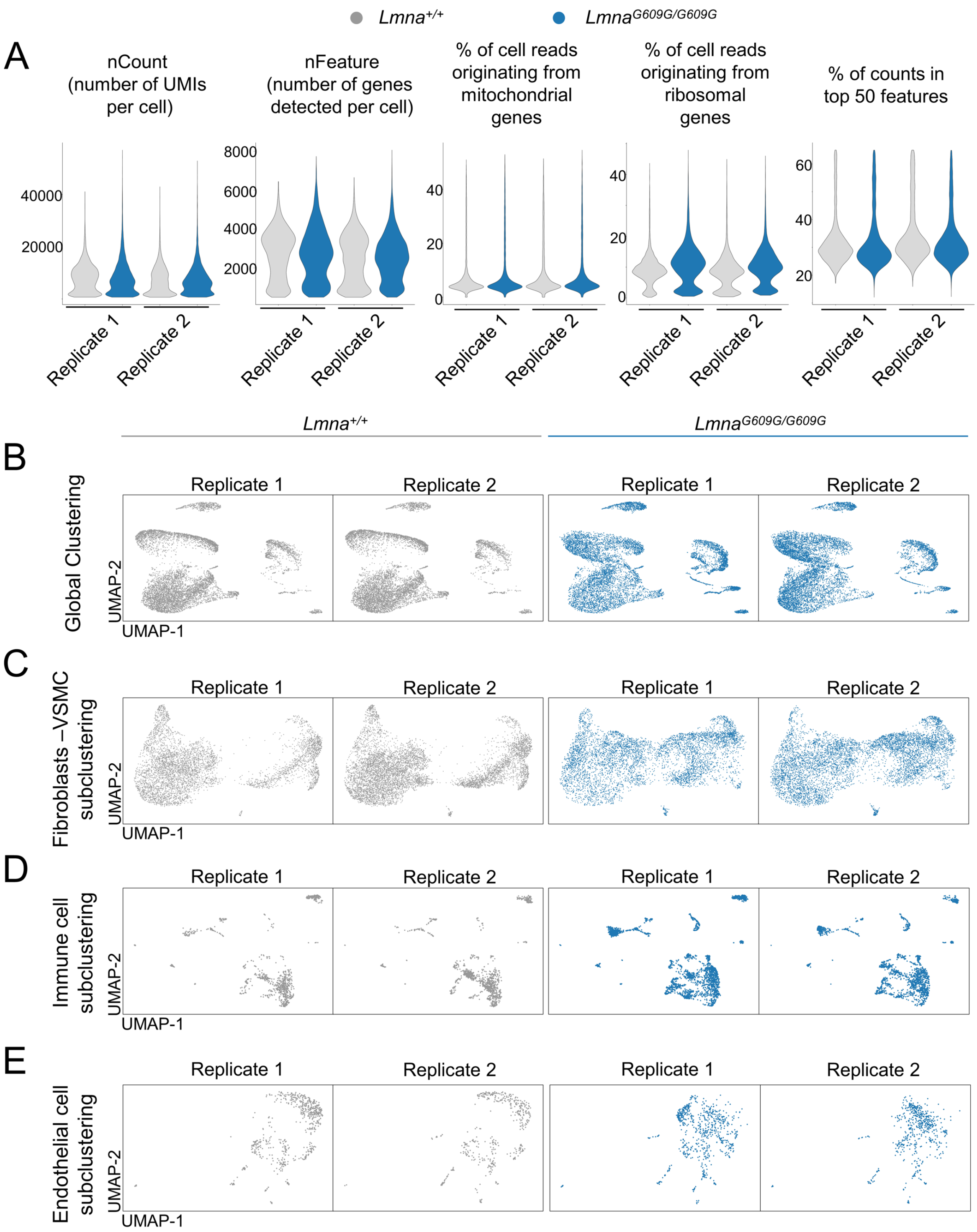

Supplemental Figure S1. Quality control parameters and replicates of single-cell RNA-seq analysis. A. Quality control parameters of scRNA-seq data from all samples. **B-E**. UMAP-based representations of scRNA-seq data for all samples derived from global clustering (B) and reclustering analyses of fibroblasts-VSMCs (C), immune cells (D), and endothelial cells (E). QC, quality control; scRNA-seq, single cell RNA sequencing; UMAP, uniform manifold approximation and projection; UMI, Unique Molecular Identifier; VSMCs, vascular smooth muscle cells.

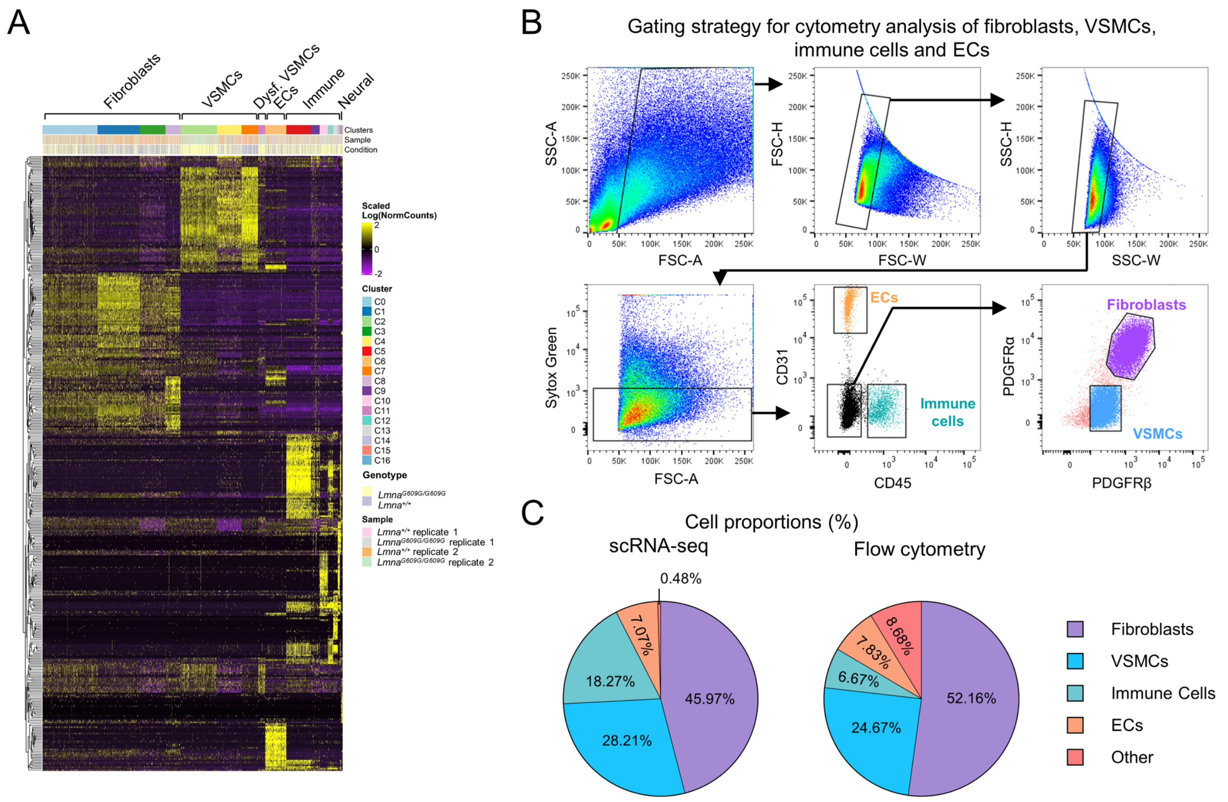

Supplemental Figure S2. Top markers and proportions of aortic cell types identified by single-cell RNA-seq and flow cytometry. A. Heat map displaying the top 30 markers for each cell cluster. **B.** Gating strategy for flow cytometry analysis of fibroblasts, VSMCs, immune cells, and ECs from mouse aorta. **C.** Pie diagrams showing proportions of the main aortic cell types identified by scRNA-seq data analysis and flow cytometry. Dysf., dysfunctional; ECs, endothelial cells; scRNA-seq, single cell RNA sequencing; VSMCs, vascular smooth muscle cells.

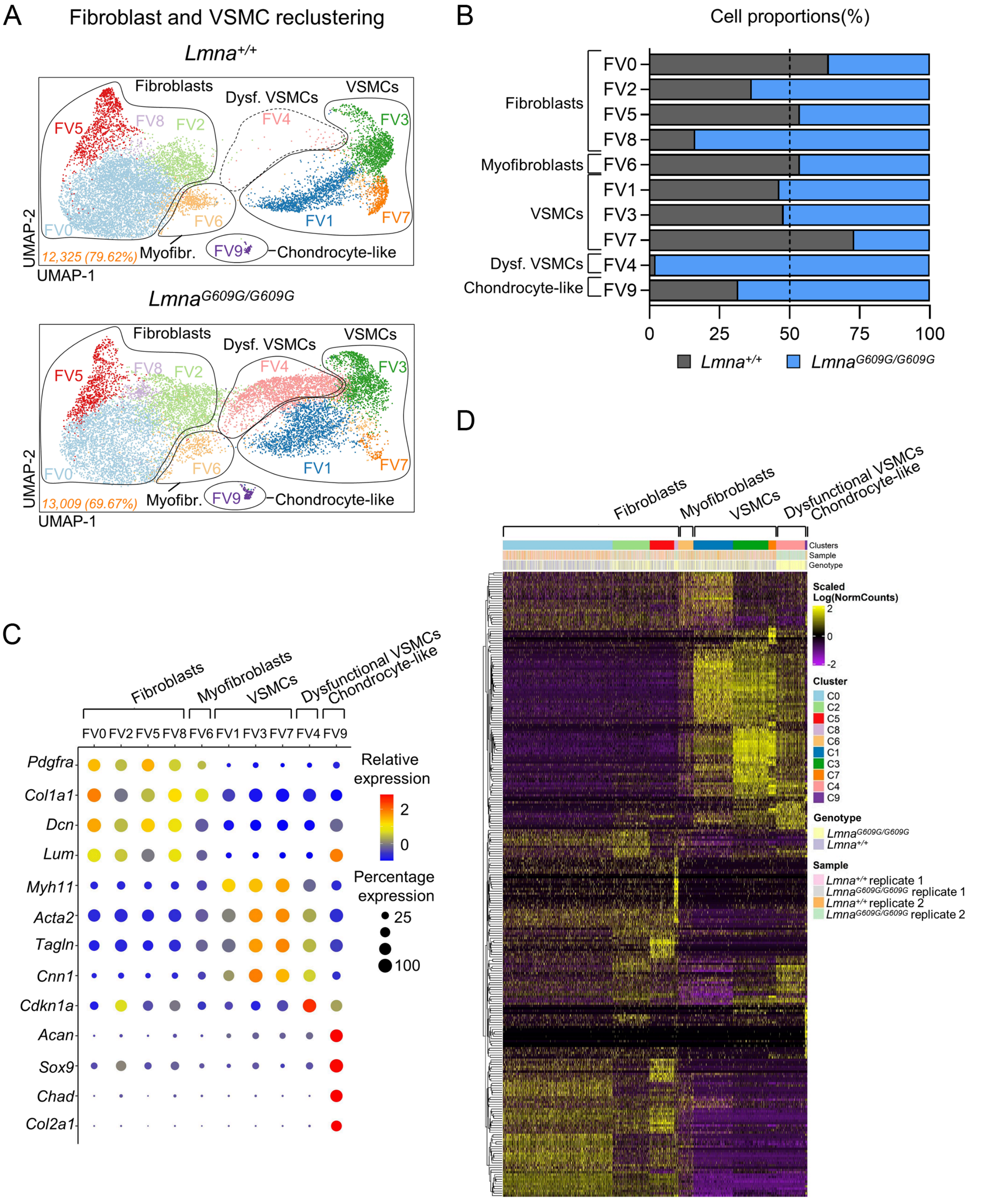

Supplemental Figure S3. Fibroblast and vascular smooth muscle cell heterogeneity in the aortas of *Lmna*$^{+/+}$ and *Lmna*$^{G609G/G609G}$ mice. A. UMAP plot of reclustered fibroblasts and VSMCs. Absolute numbers and percentages of sequenced cells are indicated for each genotype (bottom left corner)**. B.** Relative abundance of each subcluster in each genotype. The dotted line indicates 50% proportion (i.e., same number of sequenced cells in both genotypes for each cluster). **C.** Relative levels and percentage of expression of fibroblast, VSMC, and chondrocyte markers in each subcluster. **D.** Heat map displaying the top 30 markers for each subcluster of fibroblasts and VSMCs after reclustering. Dysf., dysfunctional; ECs, endothelial cells; scRNA-seq, single cell RNA sequencing; VSMCs, vascular smooth muscle cells.

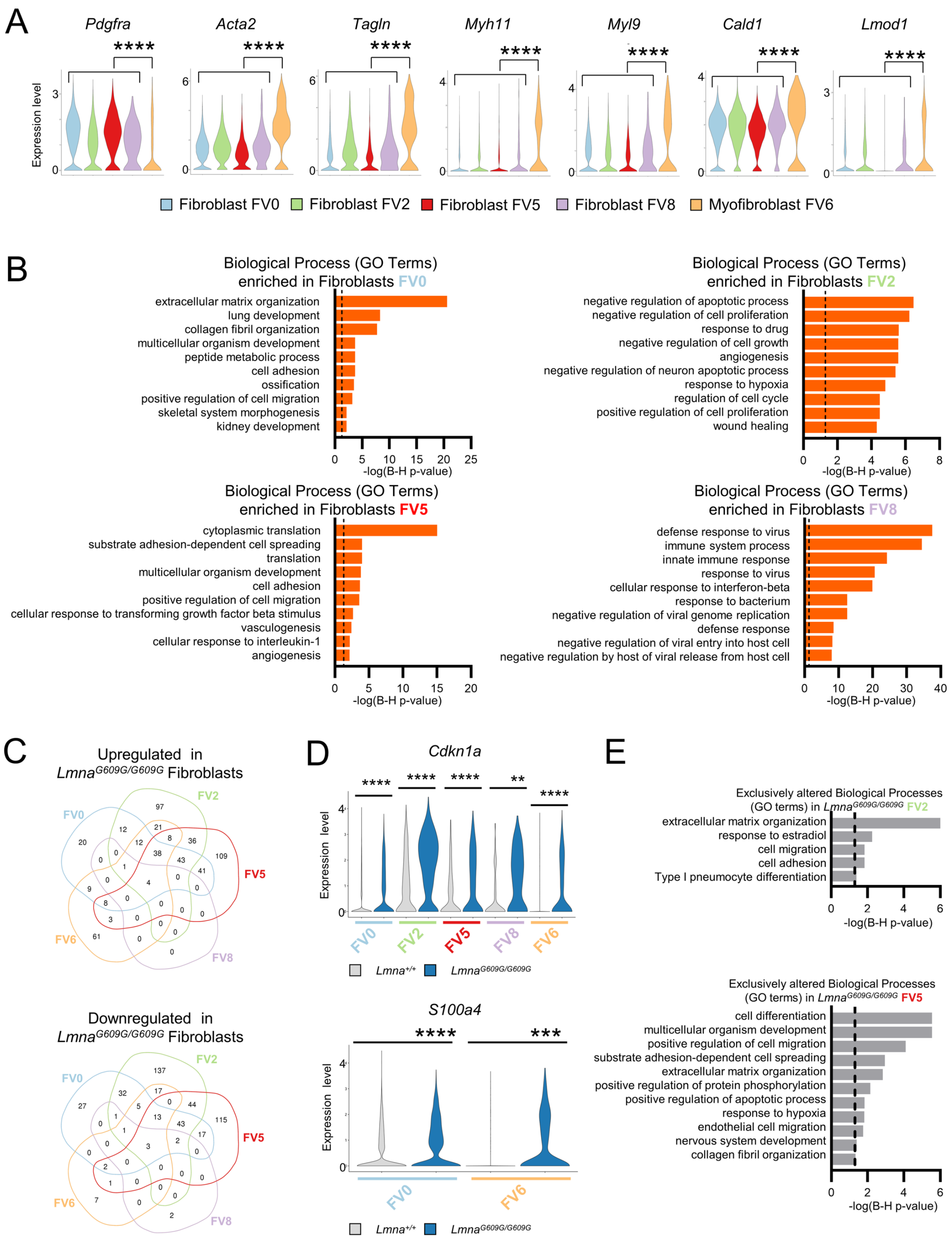

Supplemental Figure S4. Fibroblast subclusters in the aorta of *Lmna*$^{+/+}$ and *Lmna*$^{G609G/G609G}$ mice. A. Expression of *Pdgfra* and contraction-related genes in fibroblast subclusters. **B.** Top 10 enriched GO biological processes for each fibroblast subcluster. Dashed lines indicate Benjamini-Hochberg P=0.05. **C.** Venn diagrams showing the number of common and subcluster-specific gene expression changes in fibroblasts from progeroid mice relative to controls. **D.** Expression of the senescence marker *Cdkn1a* (P21) and the fibrosis-related gene *S100a4* (FSP1) in fibroblast subclusters from control and progeroid mice. **E.** GO categories specifically altered in progeroid FV2 and FV5 fibroblast subclusters. The dashed lines indicate Benjamini-Hochberg P=0.05. Statistical analysis was performed using MAST test for A and D.

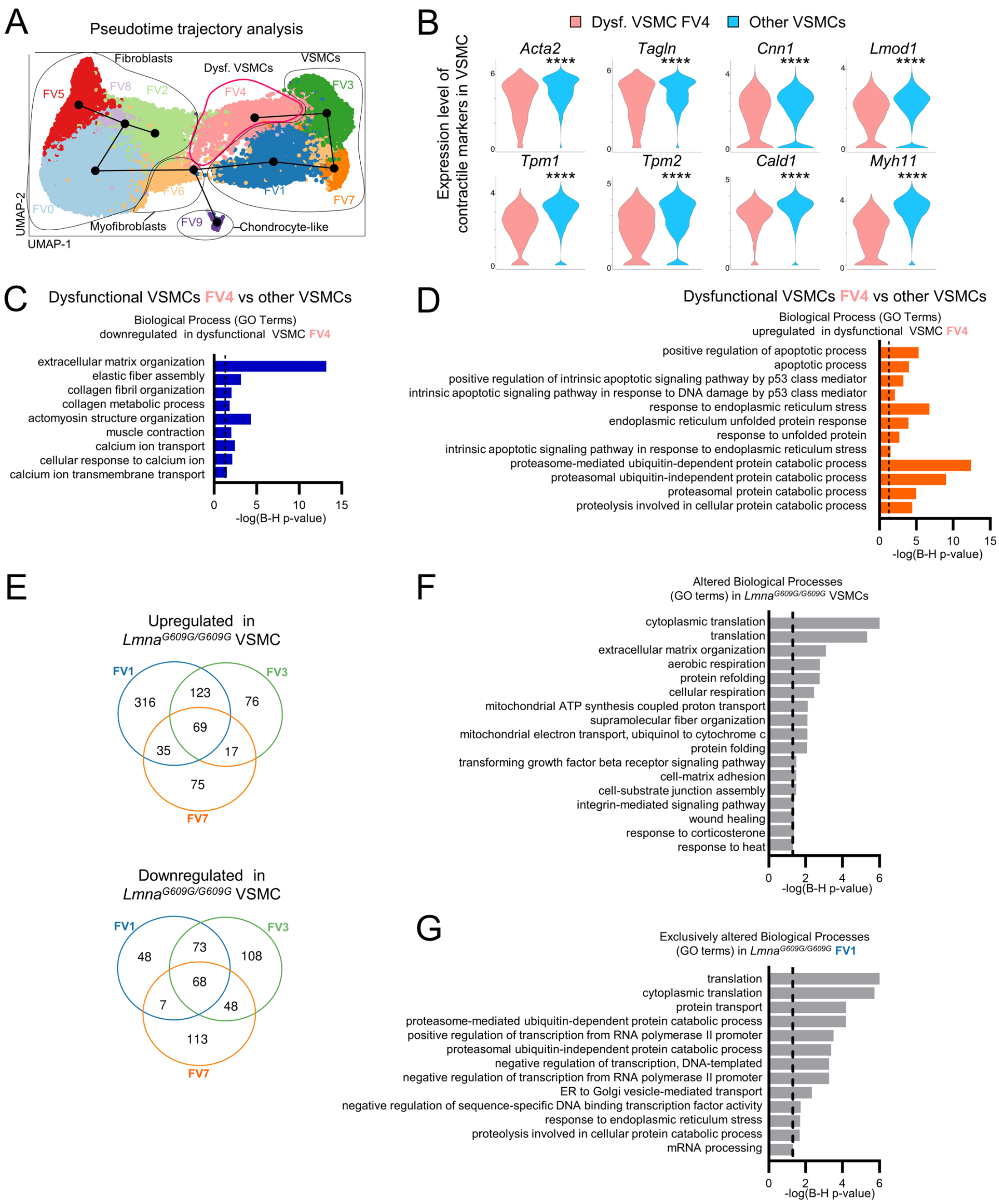

Supplemental Figure S5. VSMC subclusters in the aorta of *Lmna*$^{+/+}$ and *Lmna*$^{G609G/G609G}$ mice. A. Pseudotime trajectory analysis of reclustered fibroblasts and VSMCs. **B.** Expression of genes encoding cytoskeletal contractile proteins in VSMC clusters and showing downregulation in dysfunctional VSMCs (FV4) relative to functional VSMCs. **C, D.** Selected GO biological processes related to downregulated (C) and upregulated (D) genes in dysfunctional VSMCs (FV4) relative to the rest of VSMCs. The dashed line indicates Benjamini-Hochberg P=0.05. **E.** Venn diagrams showing the number of common and subcluster-specific gene expression changes in VSMC subclusters from progeroid mice relative to controls. **F, G.** GO categories altered in progeroid VSMCs (F, common to all VSMC subclusters; G, specifically increased abundance in FV1 VSMCs). The dashed lines indicate Benjamini-Hochberg P=0.05. Statistical analysis was performed by MAST test for B. ATP, adenosine triphosphate; Dysf., dysfunctional; ER, endoplasmic reticulum; GO, gene ontology; VSMCs, vascular smooth muscle cells.

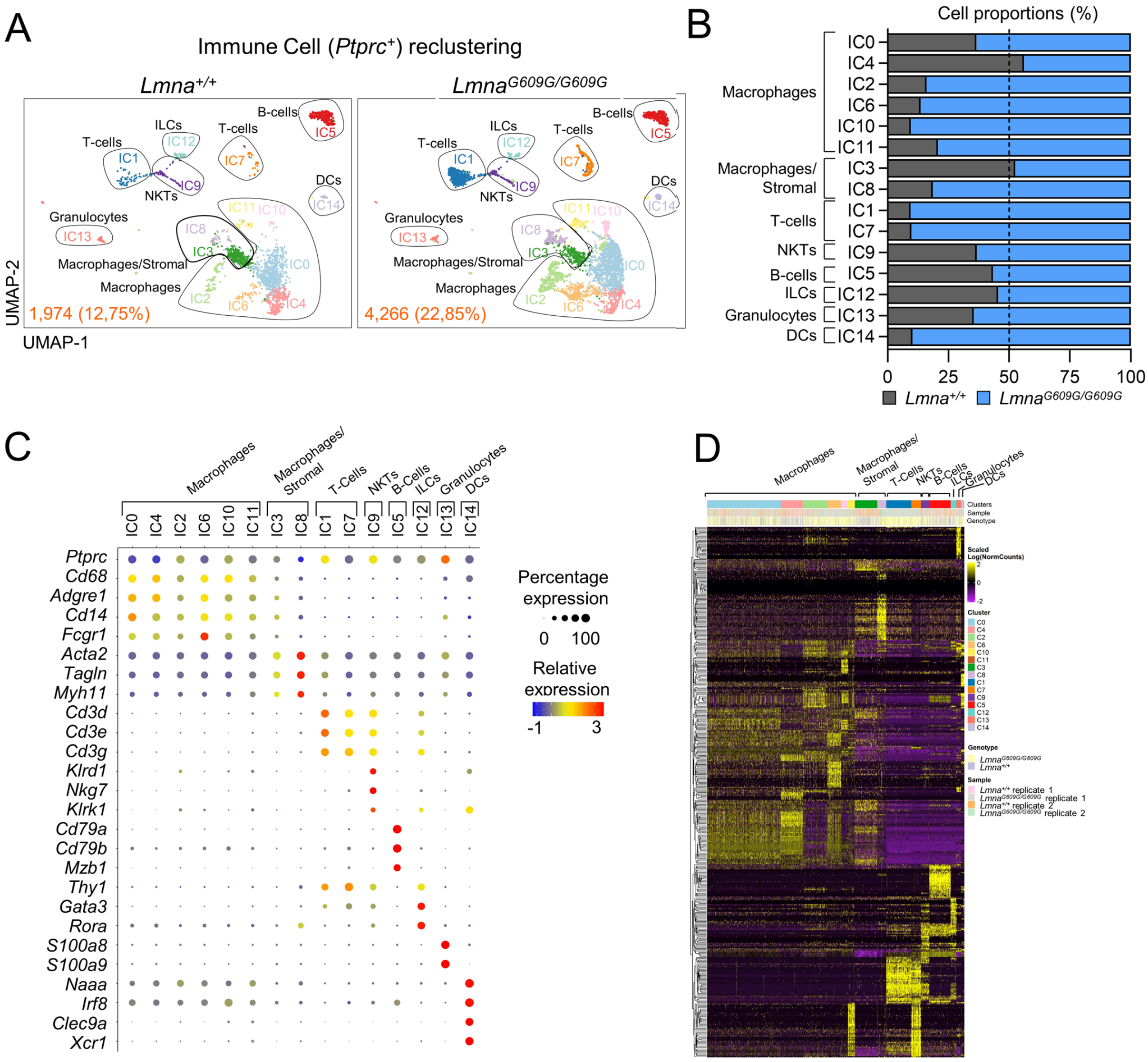

Supplemental Figure S6. Immune cell heterogeneity in the aortas of *Lmna*$^{+/+}$ and *Lmna*$^{G609G/G609G}$ mice. A. UMAP plot of reclustered immune cells, showing identified leukocyte populations. Absolute numbers and percentages of sequenced cells are indicated for each genotype (bottom left corner). **B.** Relative abundance of immune cell subclusters in control and progeroid mice. The dotted line indicates 50% proportion (i.e., same number of sequenced cells in both genotypes for each cluster). **C.** Relative levels and percentage of expression of known markers of immune cell types for each subcluster. **D.** Heat map displaying the top 30 markers for each cluster from reclustered immune cells.

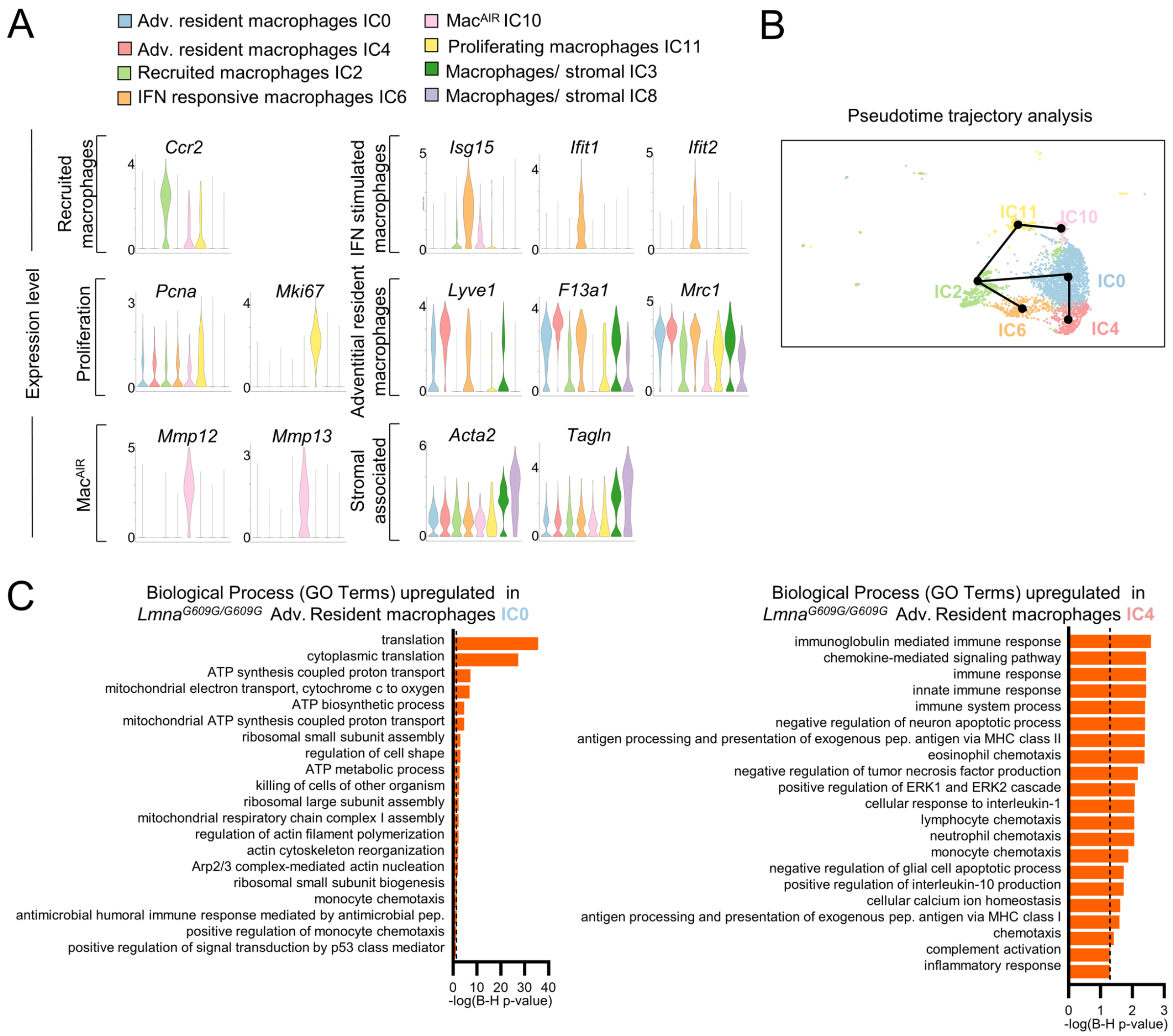

Supplemental Figure S7. Characterization of macrophages in the aortas of *Lmna*$^{+/+}$ and *Lmna*$^{G609G/G609G}$ mice. A. Expression of genes associated with recruited macrophages, response to interferon, proliferation, Mac^{AIR}, adventitial resident macrophages, and stromal cells in macrophage subclusters. **B.** Pseudotime trajectory analysis in *Lmna*$^{G609G/G609G}$ macrophages. **C.** GO categories associated with upregulated genes in adventitial resident macrophages (IC0 and IC4) from *Lmna*$^{G609G/G609G}$ mice relative to controls. The dashed lines indicate Benjamini-Hochberg P=0.05. Adv, adventitial; ATP, adenosine triphosphate; GO, gene ontology; IFN, interferon; MacAIR, aorta intima resident macrophages; MHC, major histocompatibility complex; pep., peptide.

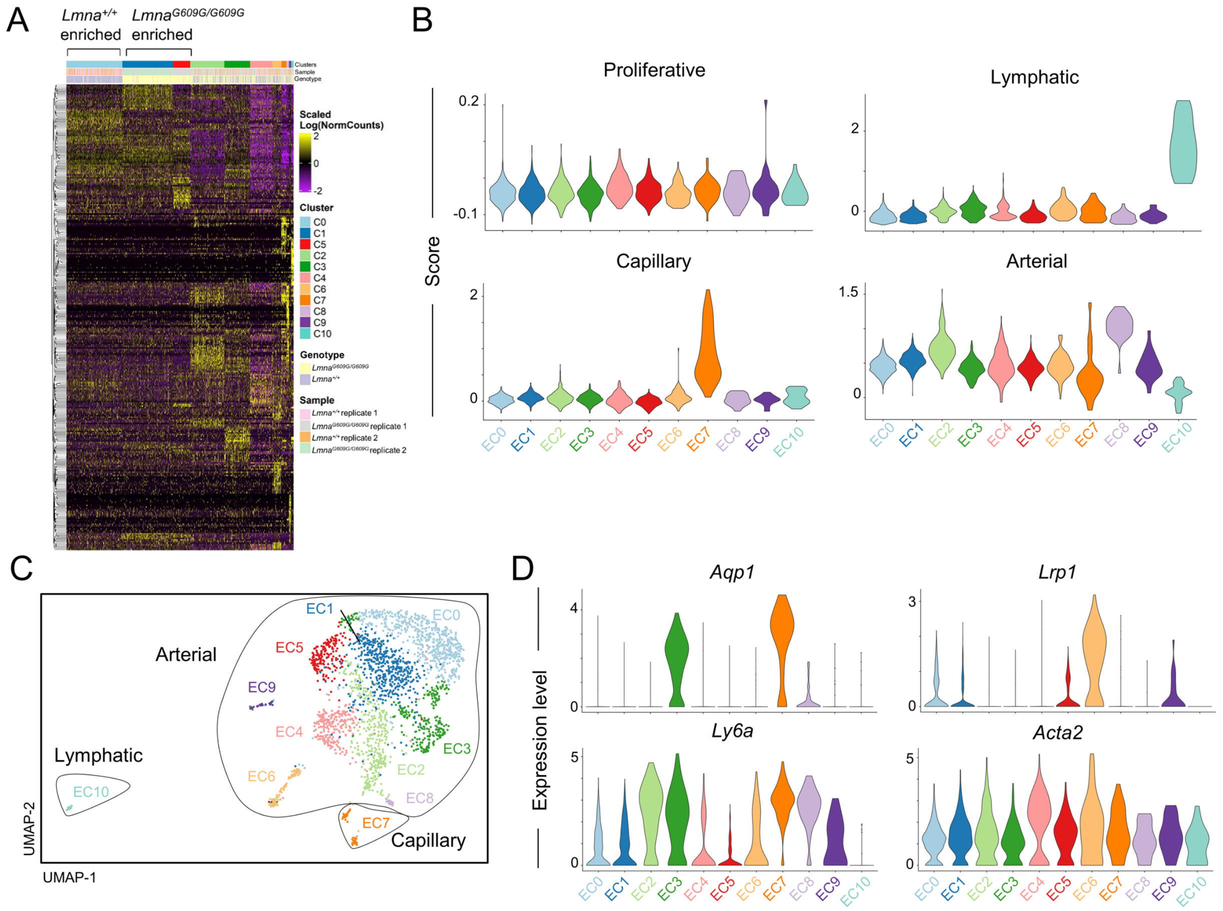

Supplemental Figure S8. Endothelial cell profiling in the aortas of *Lmna*$^{+/+}$ and *Lmna*$^{G609G/G609G}$ mice. A. Heat map displaying the top 30 markers for each subcluster from reclustered ECs. **B.** Gene expression scores associated with proliferative, capillary, lymphatic, and arterial ECs for each subcluster from reclustered ECs. **C.** UMAP plot of reclustered ECs showing distinct EC subpopulations identified as capillary, lymphatic, or arterial ECs. **D.** Expression of markers used to identify EC subpolulations EC2, EC3, EC4 and EC6.

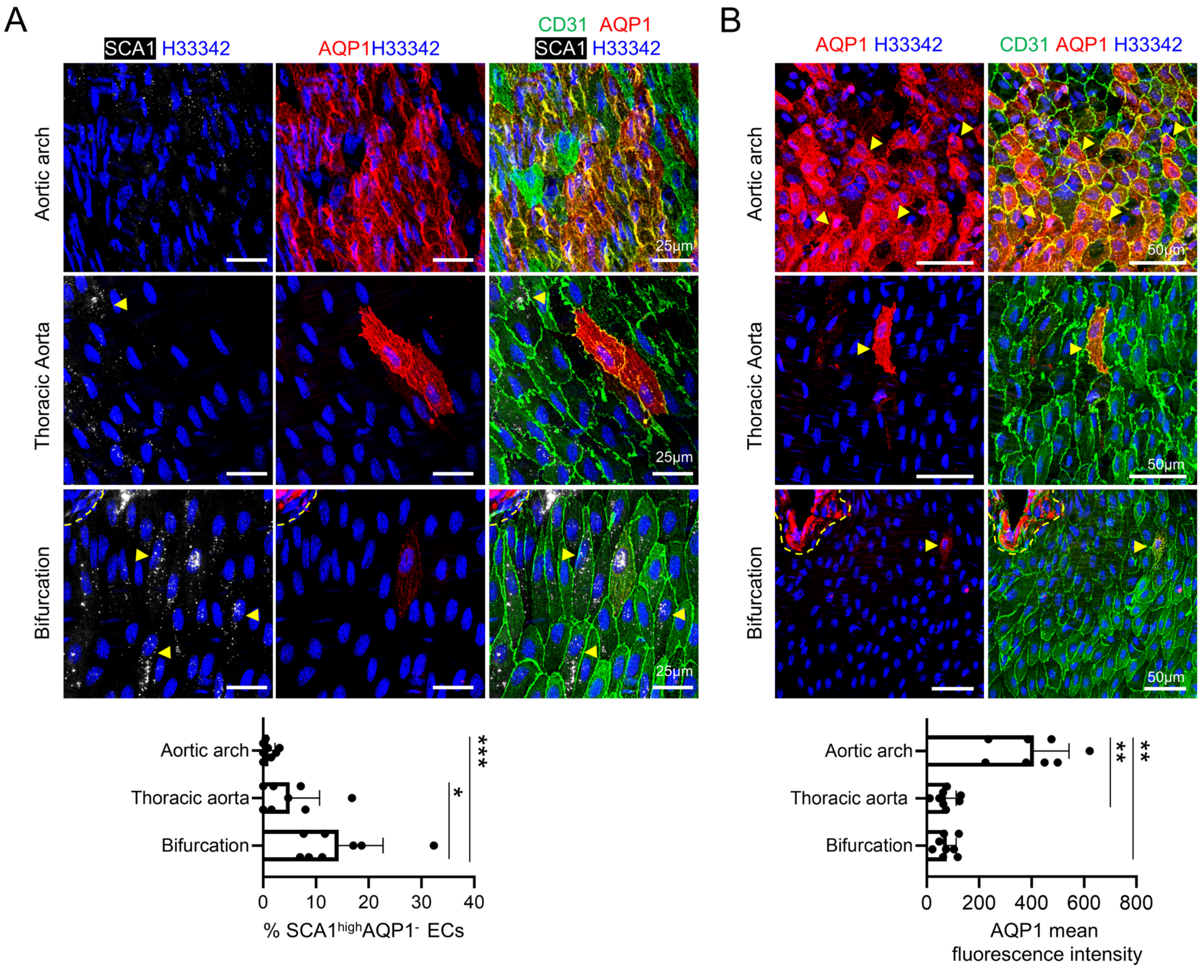

Supplemental Figure S9. Localization of endothelial subclusters EC2 and EC3 in the aorta of *Lmna*$^{+/+}$ mice. A. Representative *en face* immunofluorescence images of aortic arch, thoracic aorta areas without bifurcations, and thoracic aorta areas close to bifurcations. Representative images show ECs (CD31, green), SCA1 (encoded by *Ly6a*) (white), AQP1 (red), and nuclei (Hoechst 33342, blue). Yellow arrowheads indicate examples of SCA1^{high}AQP1^{-} ECs. Graph shows quantification of EC2 distribution as the percentage of SCA1^{high}AQP1^{-} ECs (n=8). Mean values for each individual were determined by averaging the values from 2-5 different fields. **B.** Representative *en face* immunofluorescence images of aortic arch, thoracic aorta without bifurcations, and thoracic aorta area close to bifurcations. Images show ECs (CD31, green), AQP1 (red), and nuclei (Hoechst 33342, blue). Yellow arrowheads indicate examples of AQP1^{+} ECs. Graph shows quantification of EC3 distribution as the AQP1 mean fluorescence intensity (excluding the area within bifurcations) (n=8). Mean values for each individual mouse were determined by averaging the values from 1-9 different fields per region (aortic arch, 2-4; thoracic aorta, 3-9; bifurcations, 1-5). Yellow dashed lines indicate bifurcations. Data are shown as mean+SD. Statistical analysis was performed by Kruskal-Wallis tests (A) and 1-way ANOVA (B). Scale bars, 25 µm (A), 50 µm (B). ECs, endothelial cells.

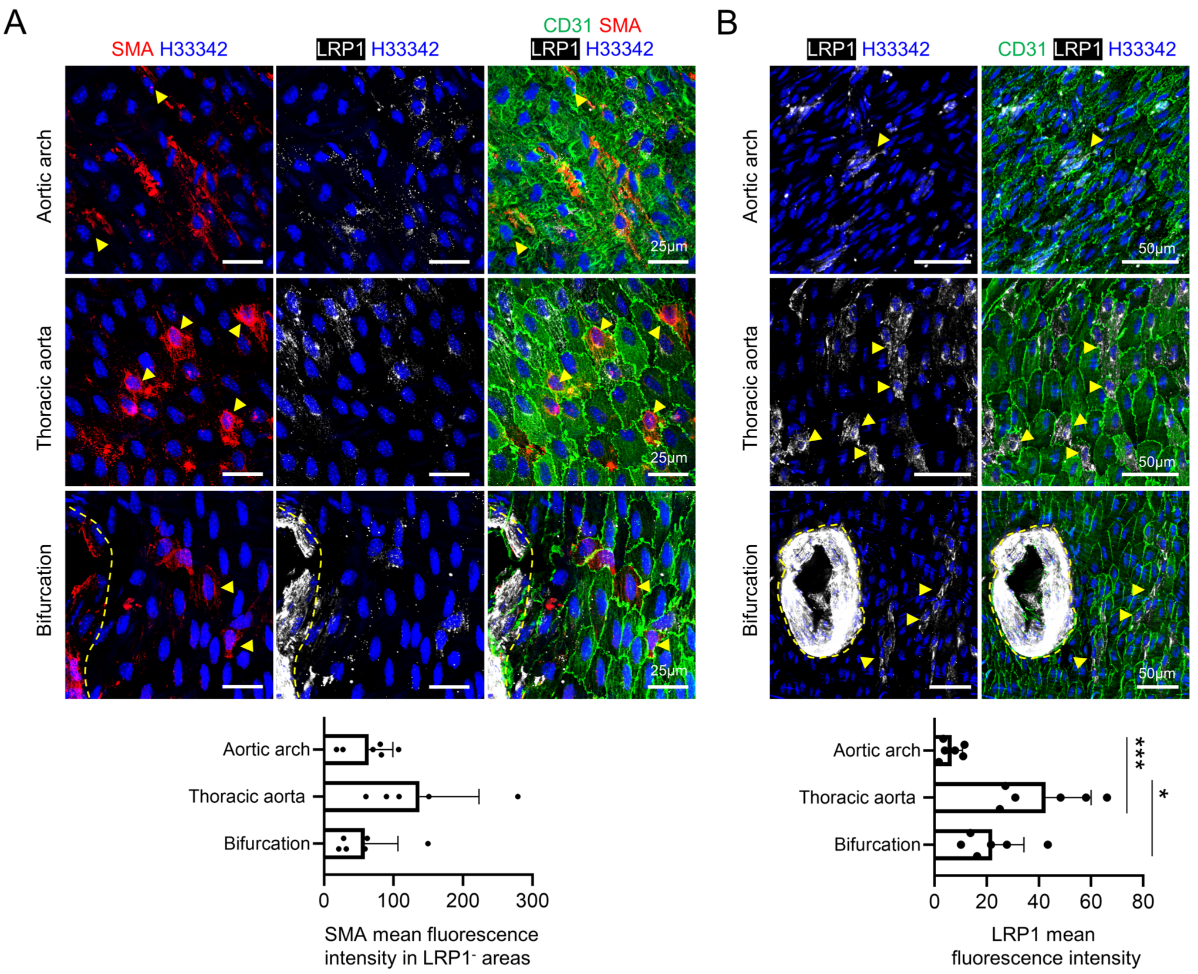

Supplemental Figure S10. Localization of endothelial subclusters EC4 and EC6 in the aorta of *Lmna*$^{+/+}$ mice. A. Representative e*n face* immunofluorescence images of aortic arch, thoracic aorta areas without bifurcations, and thoracic aorta areas close to bifurcations. Images show ECs (CD31, green), LRP1 (white), SMA (smooth muscle actin, encoded by *Acta2*) (red), and nuclei (Hoechst 33342, blue). Yellow arrowheads indicate examples of SMA^{high}LRP1^{-} ECs. Graph shows quantification of EC4 distribution as SMA mean fluorescence intensity in LRP1^{-} areas (excluding the area within bifurcations) (n=5-6). Mean values for each individual mouse were determined by averaging the values from 1-3 different fields. **B.** Representative e*n face* immunofluorescence images of aortic arch, thoracic aorta without bifurcations, and thoracic aorta area close to bifurcations. Images show ECs (CD31, green), LRP1 (white), and nuclei (Hoechst 33342, blue). Yellow arrowheads indicate examples of LRP1^{+} ECs. Graph shows quantification of EC6 distribution as the LRP1 mean fluorescence intensity (excluding the area within bifurcations and areas with immune cells expressing high levels of LRP1) (n=6). Mean values for each individual were determined by averaging the values from 1-9 different fields per region (aortic arch, 2-5; thoracic aorta, 1-9; bifurcations, 1-2). Yellow dashed lines indicate bifurcations. Data are shown as mean+SD. Statistical analysis was performed by 1-way ANOVA (A) and Kruskal-Wallis test (B). Scale bars, 25 µm (A), 50 µm (B). ECs, endothelial cells.

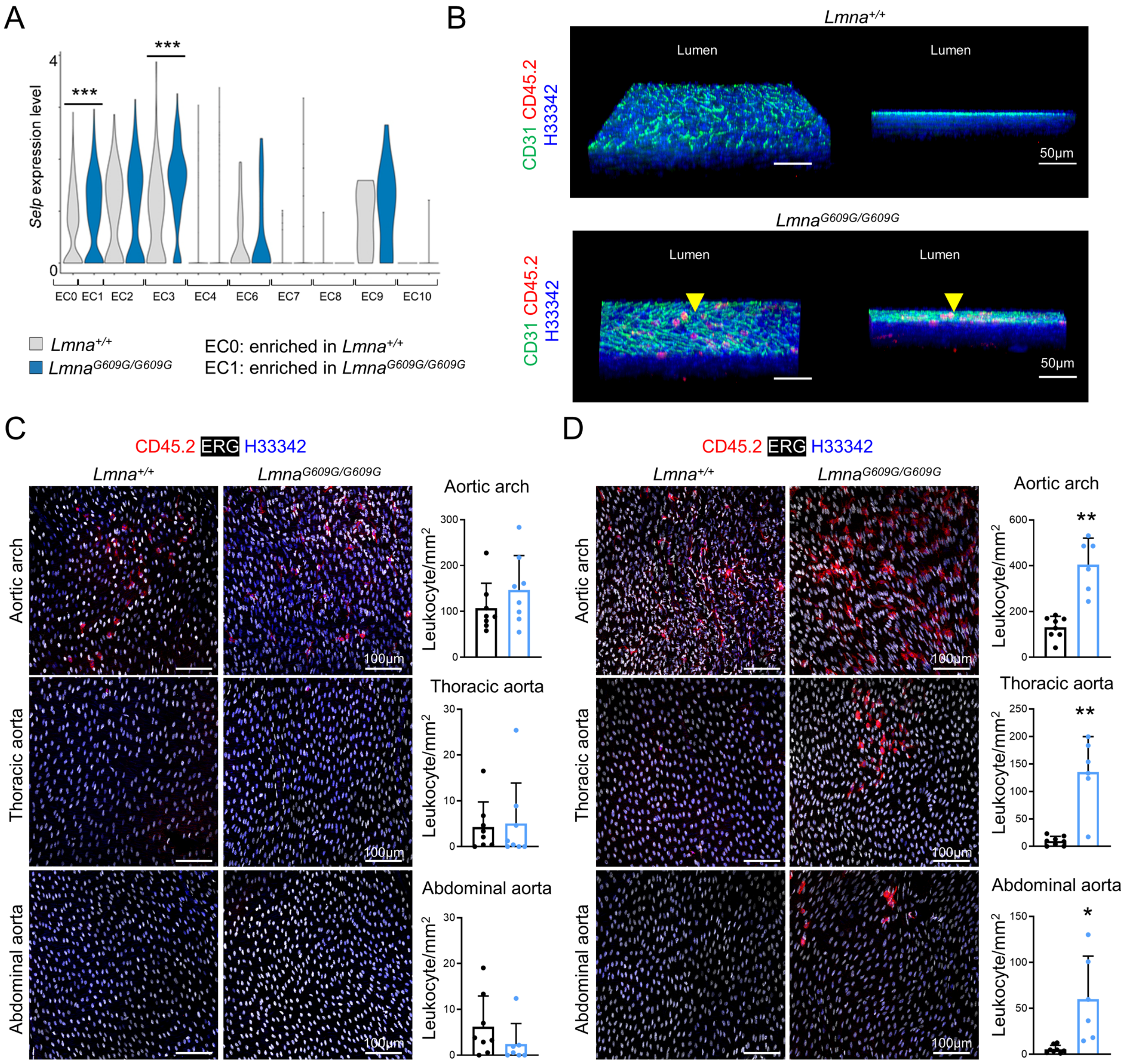

Supplemental Figure S11. Temporal and regional characterization of intimal leukocyte accumulation in aortas of *Lmna*$^{+/+}$ and *Lmna*$^{G609G/G609G}$ mice. A. *Selp* expression in aortic endothelial subclusters from control and progeroid mice (results from scRNA-seq analysis). **B.** Three-dimensional reconstruction of *en face* immunofluorescence staining of thoracic aortas showing ECs (CD31, green), leukocytes (CD45.2, red), and nuclei (Hoechst 33342, blue). Yellow arrowheads show CD45.2^{+} cells located at the level of luminal ECs in *Lmna*$^{G609G/G609G}$ mice. **C, D**. Representative *en face* immunofluorescence images of aortic arch, thoracic aorta, and abdominal aorta from 8-week-old (C, n=8) and 14-week-old (D, n=6-8) *Lmna*$^{+/+}$ and *Lmna*$^{G609G/G609G}$ mice. Representative images show EC nuclei (ERG, white), leukocytes (CD45.2, red), and nuclei (Hoechst 33342, blue). Graphs show the quantification of intimal leukocytes in each aortic region. Mean values for each mouse were determined by averaging the number of leukocytes present in 9 different fields from 2 regions of the aortic arch, 9 different fields from 3 regions of the thoracic aorta, and 4-7 different fields from 2 regions of the abdominal aorta. Data are shown as mean+SD. Statistical analysis was performed by MAST test (A), Mann-Whitney test (C), and unpaired 2-tailed *t*-test (D). Scale bar, 50 µm. ECs, endothelial cells.

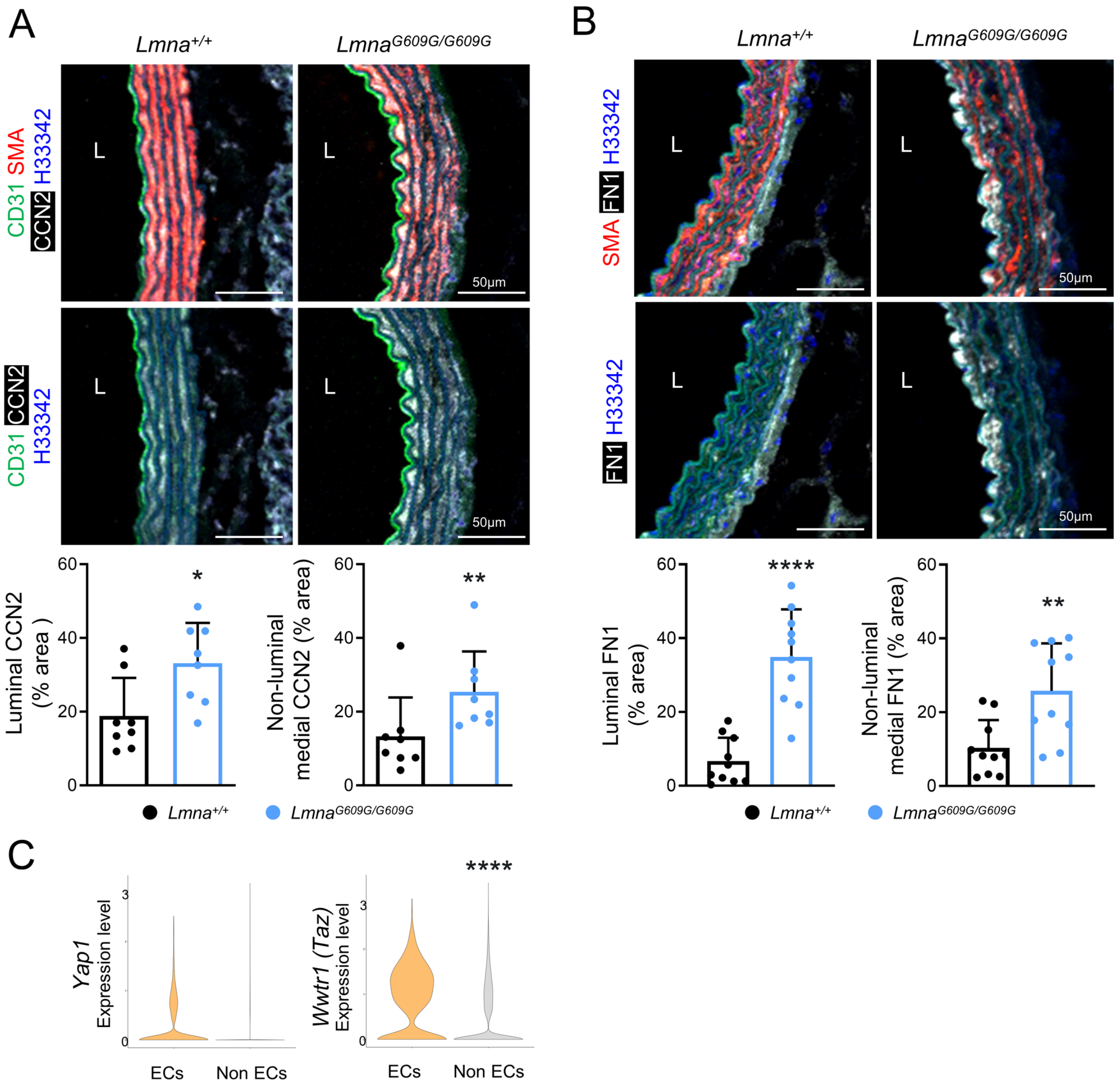

Supplemental Figure S12. Expression of YAP/TAZ targets in aortas of *Lmna*$^{+/+}$ and *Lmna*$^{G609G/G609G}$ mice. A, B. Representative immunofluorescence images of thoracic aorta cross-sections, showing smooth muscle actin (SMA, red), nuclei (Hoechst 33342, blue), CD31 (green) (A), and either cartilage tissue growth factor (CCN2, encoded by *Ctgf*) (A) or fibronectin (FN1) (B) (white). Graphs show the percentage of CCN2^{+} and FN1^{+} area in the luminal part of the aorta (first 10 μm from the lumen) and in the remaining medial aorta (non-luminal) (A, n=8; B, n=10). Mean values for each mouse were determined by averaging 2-4 aortic cross-sections. Autofluorescence from elastin layers is observed in the green and blue channels. Data are shown as mean+SD. **C.** *Yap1* (encoding YAP) and *Wwtr1* (encoding TAZ) expression in aortic ECs and non-endothelial cell types (non-ECs) (scRNA-seq analysis). Statistical analysis was performed using Mann-Whitney test (B), unpaired 2-tailed *t* -test (C), and MAST test (D). Scale bar, 50 μm. ECs, endothelial cells. L, lumen.

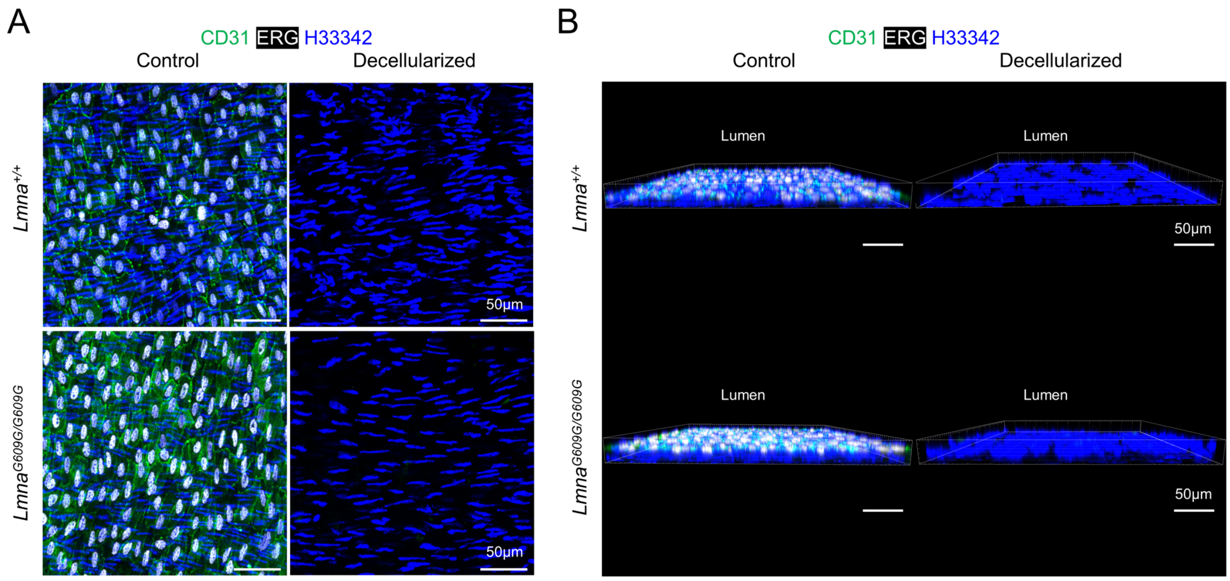

Supplemental Figure S13. Efficient removal of luminal ECs after chemical decellularization of aortas from *Lmna*$^{+/+}$ and *Lmna*$^{G609G/G609G}$ mice. A. Representative *en face* immunofluorescence images of control and decellularized thoracic aortas used for atomic force microscopy, showing ECs (CD31, green), EC nuclei (ERG, white), and nuclei (Hoechst 33342, blue). **B.** Three-dimensional reconstruction of *en face* immunofluorescence staining. Scale bar, 50 μm. ECs, endothelial cells.

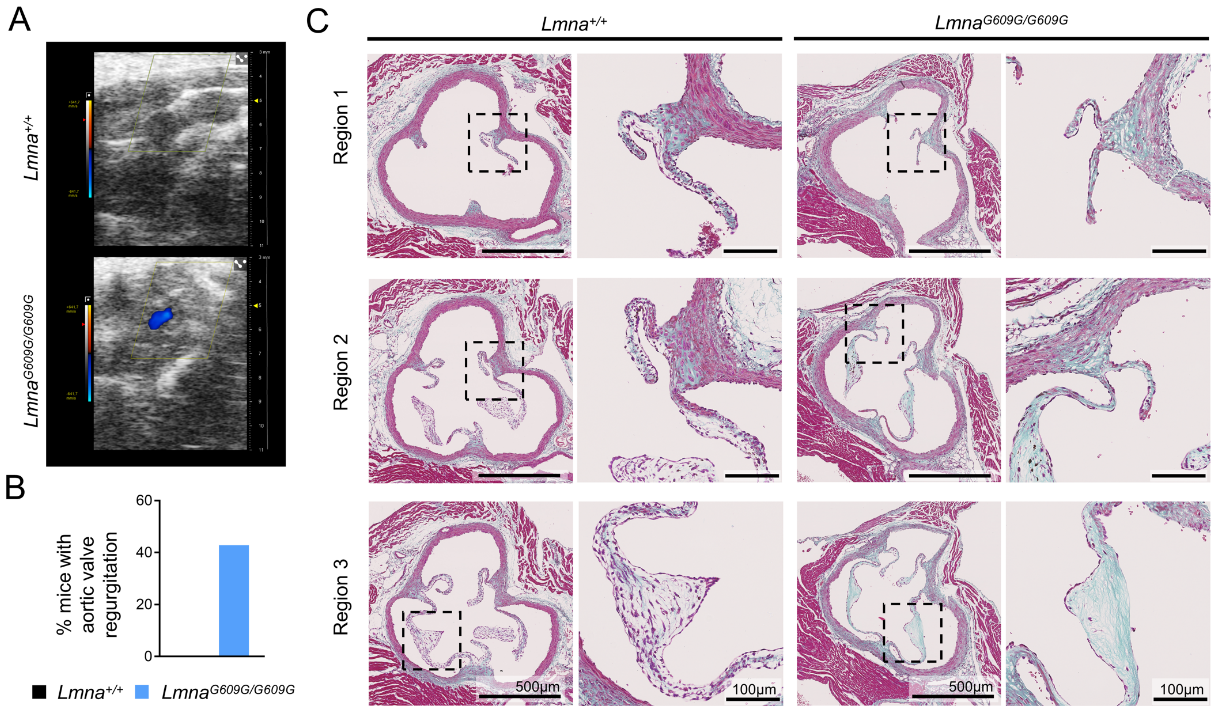

Supplemental Figure S14. Aortic valve alterations in *Lmna*$^{G609G/G609G}$ mice. A. Color Doppler ultrasound assessment of the aortic valve. Retrograde blood flow in diastole is shown in blue. **B.** Percentage of mice with aortic valve regurgitation: 0 out of 6 *Lmna*$^{+/+}$ mice; 3 out of 7 *Lmna*$^{G609G/G609G}$ mice. **C.** Representative images of Masson's trichrome staining in three different regions of the aortic valves (n=11-12). Scale bars, 500 μm (low magnification images), 100 μm (high magnification images).

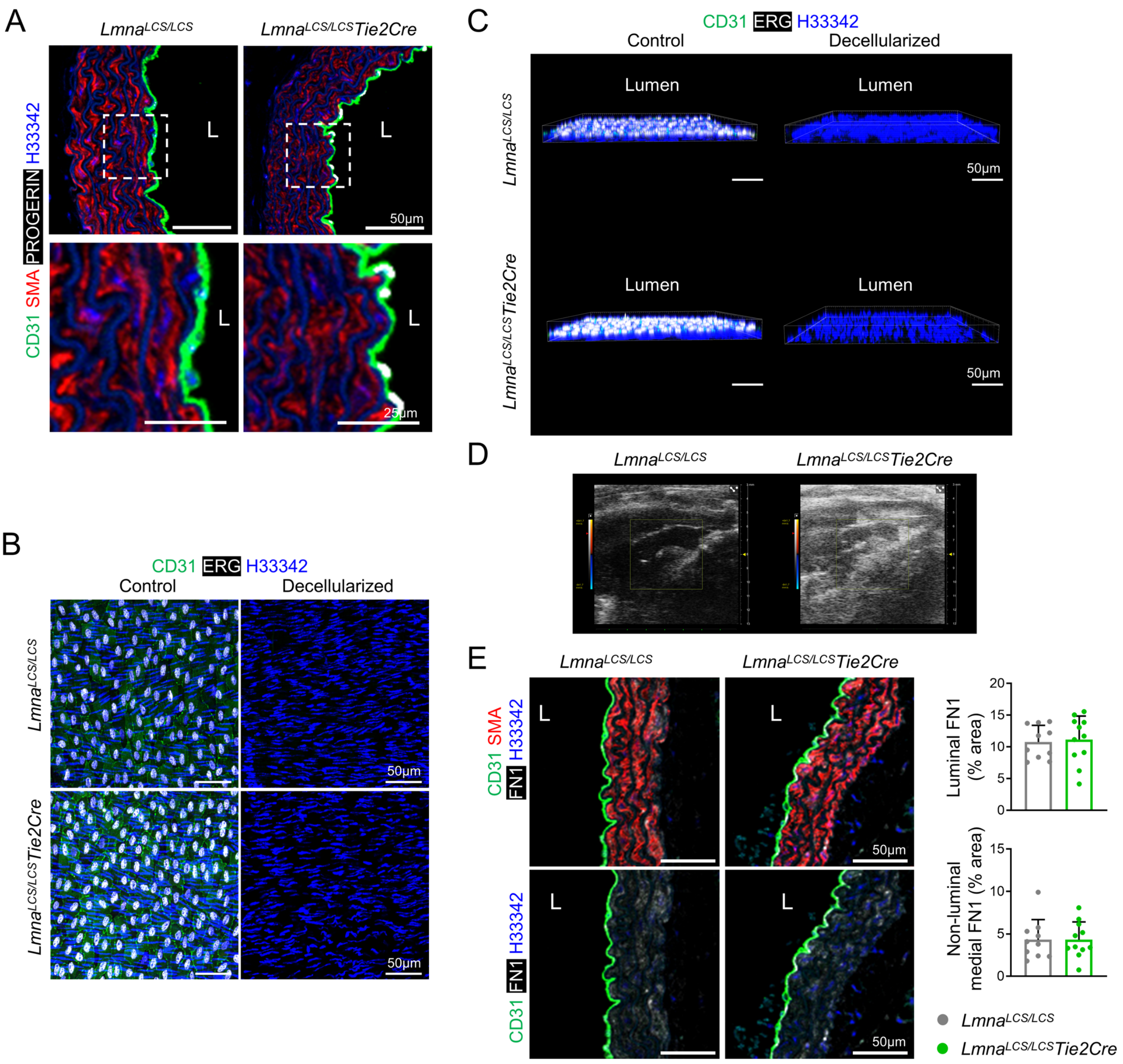

Supplemental Figure S15. Characterization of *Lmna$^{LCS/LCS}$* and *Lmna$^{LCS/LCS}$Tie2Cre* mice. **A.** Representative immunofluorescence images of aortic arch cross-sections, showing smooth muscle actin (SMA, red), nuclei (Hoechst 33342, blue), CD31 (green), and progerin (white). Boxed areas shown at higher magnification. **B.** Representative *en face* immunofluorescence images of control and decellularized thoracic aortas, showing ECs (CD31, green), EC nuclei (ERG, white), and nuclei (Hoechst 33342, blue). **C.** Representative three-dimensional reconstruction of *en face* immunofluorescence staining shown in B. **D.** Representative images of color Doppler ultrasound assessment of the aortic valve. **E.** Representative immunofluorescence images of thoracic aorta cross-sections, showing smooth muscle actin (SMA, red), nuclei (Hoechst 33342, blue), CD31 (green) and fibronectin (FN1) (white). Graphs show the percentage of FN1^{+} area in the luminal part of the aorta (first 10 µm from the lumen) and in the remaining medial aorta (non-luminal) (n=10-11). Data are shown as mean+SD. Statistical analysis was performed by unpaired 2-tailed *t*-test (E). Scale bar, 50 µm. L, lumen.

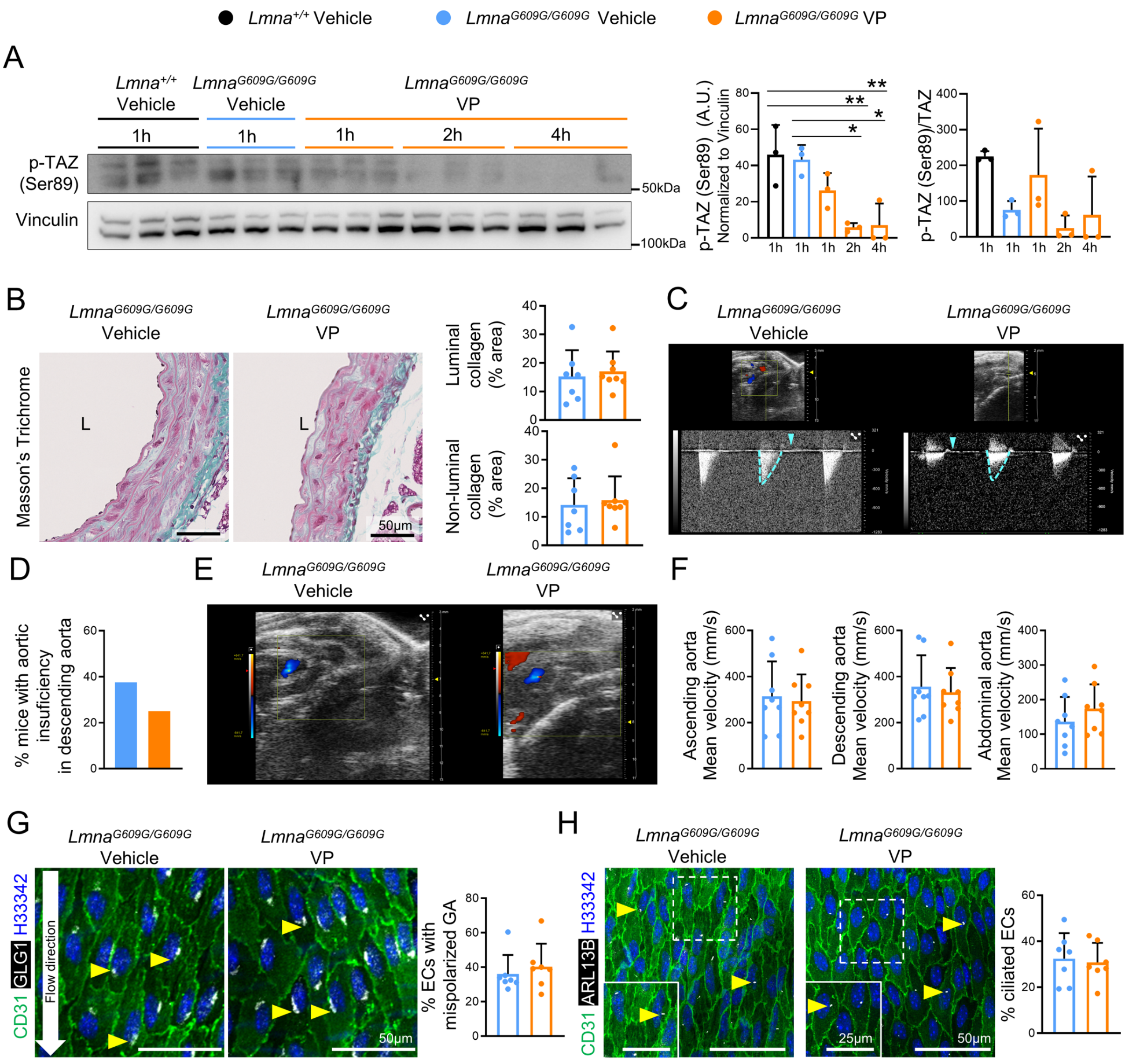

Supplemental Figure S16. YAP/TAZ inhibition with verteporfin does not reduce collagen accumulation or flow related alterations in the aorta of *Lmna*$^{G609G/G609G}$ mice. A. Western blot analysis of thoracic aorta lysates, showing phosphorylated (inactive) TAZ [p-TAZ(Ser89)] and vinculin (loading control). Graphs show the quantification of p-TAZ(Ser89) expression normalized to vinculin and the ratio of p-TAZ(Ser89)/total TAZ (total TAZ shown in Figure 7A) (n=3). **B.** Masson's trichrome staining of thoracic aorta cross-sections. Graphs show the percentage of collagen-containing area in the luminal region of the aorta (first 10 µm from the lumen) and in the remaining medial aorta (non-luminal) (n=7-8). **C.** Top: Representative ultrasound images of the descending aorta. Bottom: Pulse wave Doppler mode graphs show mean velocity in systole indicated with blue dashed lines (below baseline); retrograde flow in diastole is detected as a plateau above baseline (light blue arrowhead). **D.** Percentage of mice with aortic insufficiency (retrograde flow) in the descending aorta: 3 out of 8 *Lmna*$^{G609G/G609G}$ mice; 2 out of 8 *Lmna*$^{G609G/G609G}$ VP mice. **E.** Color Doppler ultrasound assessment of the aortic valve. Retrograde blood flow in diastole is represented in blue. **F.** Blood flow mean velocity values in the ascending, descending, and abdominal aorta (n=8). **G.** Representative *en face* immunofluorescence images of thoracic aortas, showing ECs (CD31, green), Golgi apparatus (GLG1, white), and nuclei (Hoechst 33342, blue). The graph shows the percentage of ECs with mispolarized Golgi apparatus (downstream orientation relative to nuclei, arrowheads) (n=7). Mean values for each mouse were determined by averaging the

percentages from 3 fields. **H.** Representative *en face* immunofluorescence images of thoracic aortas, showing ECs (CD31, green), primary cilia (ARL13B, white), and nuclei (Hoechst 33342, blue). The graph shows the percentage of ciliated ECs (arrowheads) (n=7). Mean values for each mouse were determined by averaging the percentages from 3 fields (n=7). Boxed areas are shown at higher magnification. Statistical analysis was performed using 1-way ANOVA (A), 2-tailed *t*-test (B, luminal collagen; E, ascending and abdominal aorta; H), Mann-Whitney test (B, non-luminal collagen; G), and 2-sided Fisher exact test (D). Scale bars, 50 μm and 25 μm (insets in H).; ECs, endothelial cells; GA, Golgi apparatus; L, lumen; VP, verteporfin.

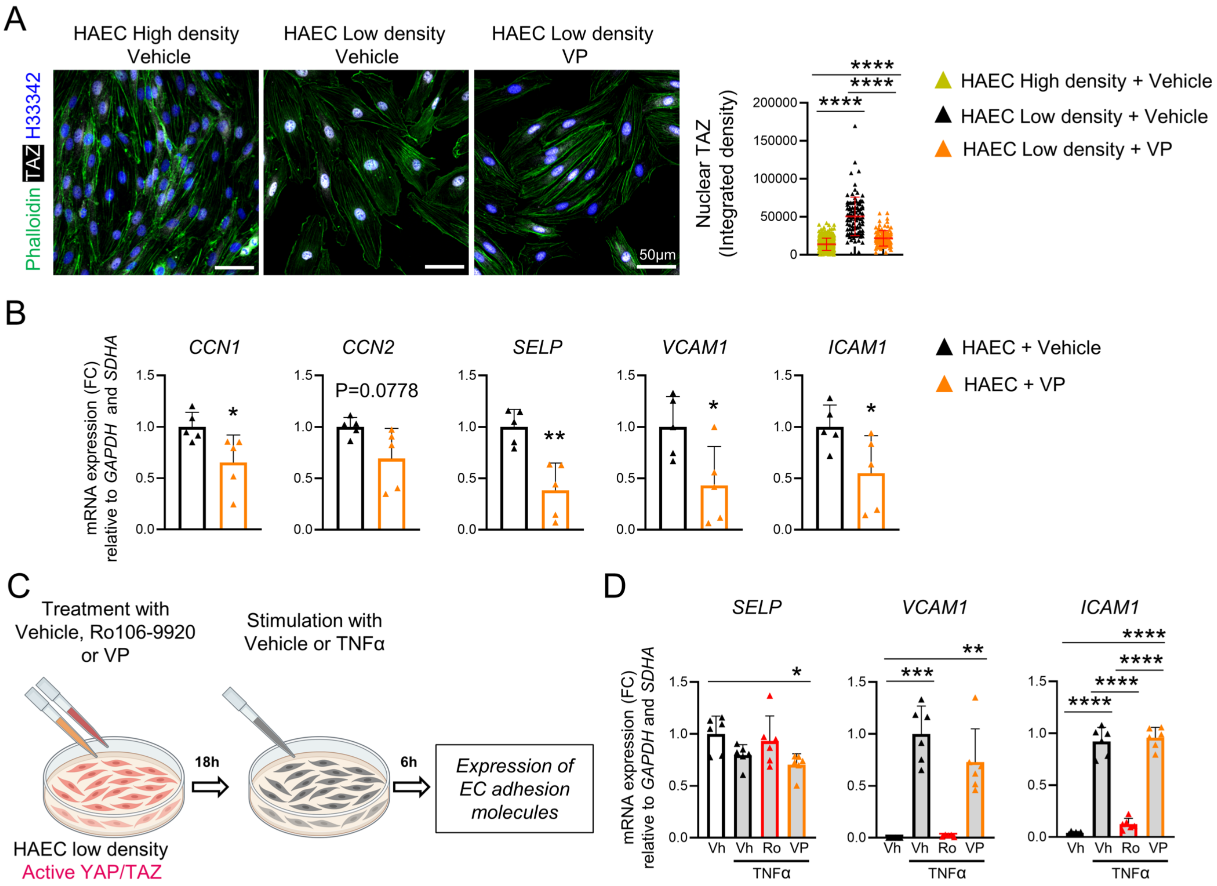

Supplemental Figure S17. YAP/TAZ inhibition with verteporfin reduces the expression of endothelial adhesion molecules in HAEC cultures. A. Representative immunofluorescence images of HAECs cultured at high density (inactive YAP/TAZ) and low density (active YAP/TAZ), treated with vehicle or verteporfin for 24 h. Representative images showing phalloidin (green), TAZ (white), and nuclei (Hoechst 33342, blue). The graph shows the quantification of TAZ fluorescence intensity in the nucleus of individual cells (n=97-423 cells) obtained from 6 different fields. **B.** Expression of canonical YAP/TAZ target genes (*CCN1* and *CCN2*) and endothelial adhesion molecules (*SELP*, *VCAM1*, and *ICAM1*) determined by RT-qPCR in control (vehicle) and verteporfin-treated HAECs cultured at low density (n=5 from two independent experiments). **C.** Experimental design. HAECs cultured at low density (active YAP/TAZ) were treated with either vehicle, 2 µM Ro106-9920, or 1 µM verteporfin. After 18 h, cells were stimulated with 5 ng ml^{-1} TNFα in the presence of vehicle, Ro106-9920, or verteporfin for 6 h, and RNA was collected for RT-qPCR assays. **D.** Expression of endothelial adhesion molecules in HAECs cultured at low density after treatment determined by RT-qPCR (n=5-6 from two independent experiments). Data in A are shown as mean±SD, and in B and D as mean+SD. Statistical analysis was performed by Kruskal-Wallis tests ([A and D (*SELP*, *VCAM1*)], unpaired 2-tailed *t*-test (B), and 1-way ANOVA (D, *ICAM1*). Outliers assessed by ROUT test in D were excluded for analysis. Scale bar, 50 µm. VP, verteporfin.

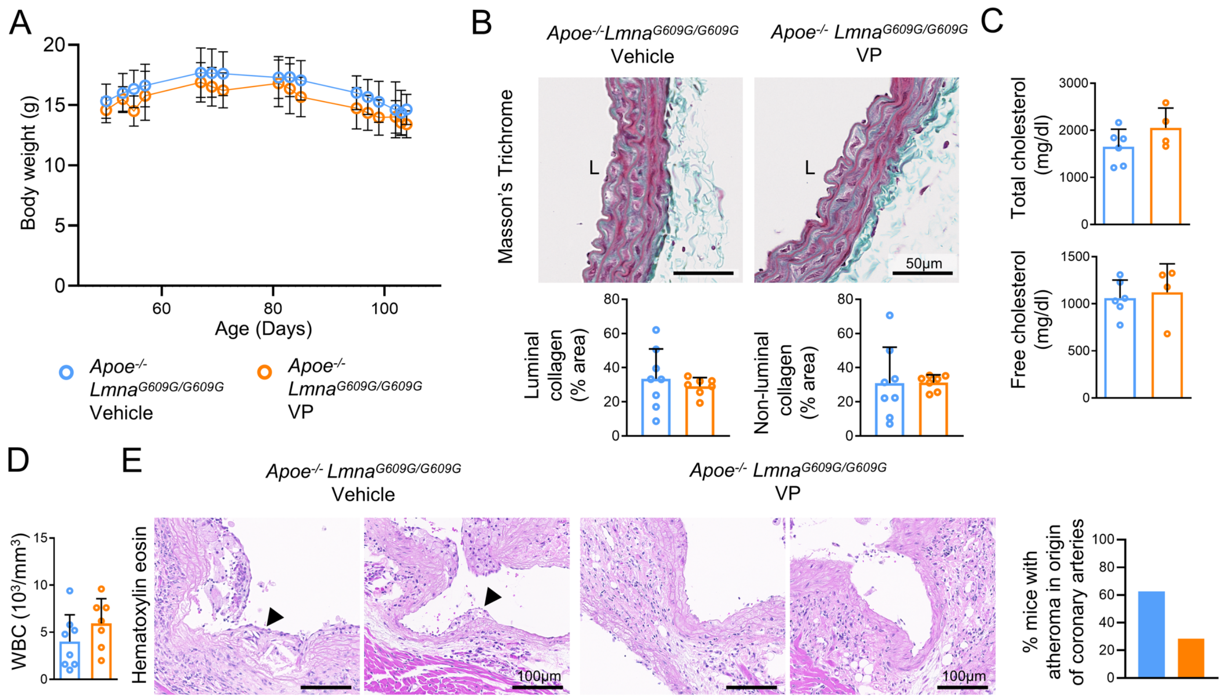

Supplemental Figure S18. Verteporfin treatment does not alter body weight, aortic collagen accumulation, serum cholesterol levels, and circulating white blood cell counts in *Apoe*$^{-/-}$*Lmna*$^{G609G/G609G}$ mice. *Apoe*$^{-/-}$*Lmna*$^{G609G/G609G}$ mice fed a high-fat diet were treated with verteporfin or vehicle. **A.** Body weight evolution of vehicle- and verteporfin-treated *Apoe*$^{-/-}$ *Lmna*$^{G609G/G609G}$ mice. **B.** Representative images of Masson's trichrome staining of thoracic aorta. Graphs show the percentage of area containing collagen in the luminal region of the aorta (first 10 µm from the lumen) and in the remaining medial aorta (non-luminal) (n=7-8). **C.** Serum concentration of total and free cholesterol (n=4-6). **D.** Circulating white blood cell counts (n=7-8). **E.** Representative images of hematoxylin-eosin staining of the origin of coronary arteries, close to the aortic root. Black arrowheads show examples of plaques, and the graph shows the percentage of mice with plaques in this region: 5 out of 8 *Apoe*$^{-/-}$*Lmna*$^{G609G/G609G}$ Vehicle mice; 2 out of 7 *Apoe*$^{-/-}$*Lmna*$^{G609G/G609G}$ VP mice) Data are shown as mean+SD. Statistical analysis was done using the mixed effects model with the Geisser–Greenhouse correction (A), unpaired 2-tailed *t*-test (B, C and D), and 2-sided Fisher exact test (E). Scale bar, 50 µm (B), 100 µm (E). HDL, high-density lipoprotein; L, lumen; LDL, low-density lipoprotein; VP, verteporfin; WBC, white blood cell.

LEGENDS FOR SUPPLEMENTAL TABLES 1–18

Supplemental Table 1. Differentially expressed genes in each cluster compared with the rest of cells from global clustering.

Supplemental Table 2. Differentially expressed genes in each fibroblast and VSMC cluster compared with the rest of cells from fibroblast-VSMC reclustering.

Supplemental Table 3. Gene expression analysis in FV6 compared with the rest of fibroblast clusters (FV0, FV2, FV5 and FV8) from fibroblast-VSMC reclustering.

Supplemental Table 4. Gene expression analysis in FV0, FV2, FV5 or FV8 compared with the rest of fibroblasts from fibroblast-VSMC reclustering.

Supplemental Table 5. Gene Ontology (GO) biological processes enriched in fibroblasts from FV0, FV2, FV5 and FV8.

Supplemental Table 6. Gene expression analysis in each fibroblast and VSMC cluster from HGPS mice compared with wild-type controls from fibroblast-VSMC reclustering.

Supplemental Table 7. GO Biological processes enriched in HGPS fibroblasts FV2 and FV5 compared with wild-type controls. Genes specifically altered in HGPS FV2 and FV5 compared with the rest of fibroblast clusters were used for GO analysis.

Supplemental Table 8. Gene expression analysis in FV4 compared with the rest of VSMCs (FV1, FV3, FV7) from fibroblast-VSMC reclustering.

Supplemental Table 9. GO Biological processes upregulated and downregulated in FV4 compared with the rest of VSMCs.

Supplemental Table 10. GO Biological processes enriched in all HGPS VSMCs (FV1, FV3, FV7) and specifically in HGPS FV1 compared with wild-type controls. GO analysis was performed with genes altered in all HGPS VSMC clusters or specifically in HGPS FV1 compared with the rest of VSMC clusters.

Supplemental Table 11. Differentially expressed genes in each immune cell cluster compared with the rest of cells from immune cell clustering.

Supplemental Table 12. Gene expression analysis in each immune cell cluster from HGPS mice compared with wild-type controls from immune cell reclustering.

Supplemental Table 13. GO Biological processes enriched in HGPS adventitial resident macrophage clusters IC0 and IC4 compared with wild-type controls.
.

Supplemental Table 14. Differentially expressed genes in each EC cluster compared with the rest of cells from EC reclustering.

Supplemental Table 15. Gene expression analysis in HGPS-enriched EC1 compared to wild-type-enriched EC0, and in HGPS EC2-EC10 compared to wild-type controls from EC reclustering.

Supplemental Table 16. Ingenuity Pathway Analysis to predict canonical pathway alterations in HGPS-enriched EC1 compared to wild-type-enriched EC0. Positive and negative z-scores indicate activation and inhibition of the pathway in EC1, respectively.

Supplemental Table 17. Ingenuity Pathway Analysis to predict the activation state of upstream regulators in HGPS-enriched EC1 compared to wild-type-enriched EC0. Positive and negative z-scores indicate activation and inhibition of the upstream regulator in EC1, respectively.

Supplemental Table 18. Gene sets used to generate scores for the identification of proliferating cells and lymphatic, capillary, and arterial ECs.
