# Supplementary material for: Endothelial YAP/TAZ activation promotes atherosclerosis in a mouse model of Hutchinson-Gilford progeria syndrome
Source: J Clin Invest. 2024 Oct 1;134(22):e173448. doi: 10.1172/JCI173448 (PMC11563688; doi:10.1172/JCI173448)

Full unedited blot for Figure 3E

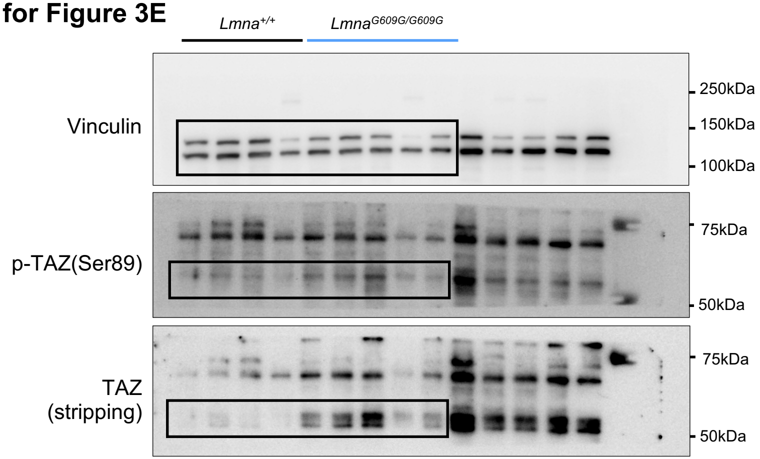

Full unedited blot for Figure 7A

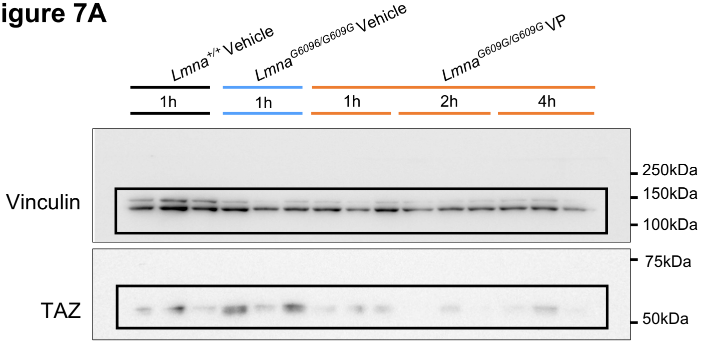

Full unedited blot for Supplemental Figure 16A

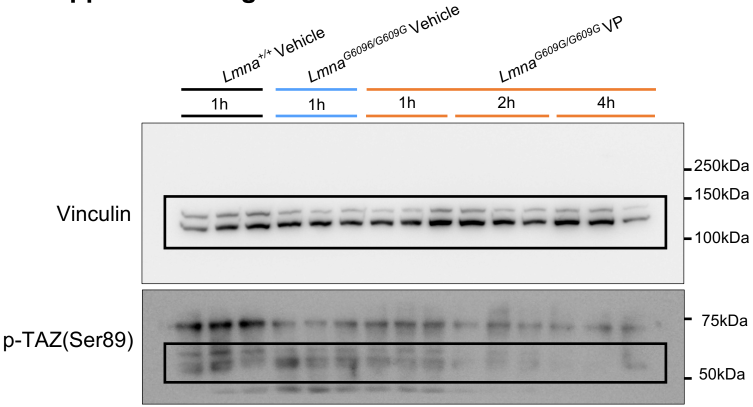

Supplement: Unedited blot and gel images [file jci-134-173448-s010.pdf]
